# Symptoms and Severe Acute Respiratory Syndrome Coronavirus 2 (SARS-CoV-2) Positivity in the General Population in the United Kingdom

**DOI:** 10.1093/cid/ciab945

**Published:** 2021-11-08

**Authors:** Karina Doris Vihta, Koen B Pouwels, Tim E A Peto, Emma Pritchard, David W Eyre, Thomas House, Owen Gethings, Ruth Studley, Emma Rourke, Duncan Cook, Ian Diamond, Derrick Crook, Philippa C Matthews, Nicole Stoesser, Ann Sarah Walker, COVID-19 Infection Survey, Emma Rourke, Ruth Studley, Tina Thomas, Daniel Ayoubkhani, Russell Black, Antonio Felton, Megan Crees, Joel Jones, Lina Lloyd, Esther Sunderland, Ann Sarah Walker, Derrick Crook, Philippa C Matthews, Tim Peto, Emma Pritchard, Nicole Stoesser, Karina Doris Vihta, Jia Wei, Alison Howarth, George Doherty, James Kavanagh, Kevin K Chau, Sarah Cameron, Phoebe Tamblin-Hopper, Magda Wolna, Rachael Brown, Stephanie B Hatch, Daniel Ebner, Lucas Martins Ferreira, Thomas Christott, Brian D Marsden, Wanwisa Dejnirattisai, Juthathip Mongkolsapaya, Sarah Hoosdally, Richard Cornall, David I Stuart, E Yvonne Jones, Gavin Screaton, Koen Pouwels, David W Eyre, Katrina Lythgoe, David Bonsall, Tanya Golubchik, Helen Fryer, John Bell, John Newton, Julie Robotham, Paul Birrell, Helena Jordan, Tim Sheppard, Graham Athey, Dan Moody, Leigh Curry, Pamela Brereton, Jodie Hay, Harper Van Steenhouse, Anna Godsmark, George Morris, Bobby Mallick, Phil Eeles, Stuart Cox, Kevin Paddon, Tim James, Sarah Cameron, Phoebe Tamblin-Hopper, Magda Wolna, Rachael Brown, Jessica Lee

**Affiliations:** Nuffield Department of Medicine, University of Oxford, Oxford, United Kingdom; The National Institute for Health Research Health Protection Research Unit in Healthcare Associated Infections and Antimicrobial Resistance at the University of Oxford, Oxford, United Kingdom; Department of Engineering, University of Oxford, Oxford, United Kingdom; The National Institute for Health Research Health Protection Research Unit in Healthcare Associated Infections and Antimicrobial Resistance at the University of Oxford, Oxford, United Kingdom; Health Economics Research Centre, Nuffield Department of Population Health, University of Oxford, Oxford, United Kingdom; Nuffield Department of Medicine, University of Oxford, Oxford, United Kingdom; The National Institute for Health Research Health Protection Research Unit in Healthcare Associated Infections and Antimicrobial Resistance at the University of Oxford, Oxford, United Kingdom; The National Institute for Health Research Oxford Biomedical Research Centre, University of Oxford, Oxford, United Kingdom; Department of Infectious Diseases and Microbiology, Oxford University Hospitals NHS Foundation Trust, John Radcliffe Hospital, Oxford, United Kingdom; Nuffield Department of Medicine, University of Oxford, Oxford, United Kingdom; The National Institute for Health Research Health Protection Research Unit in Healthcare Associated Infections and Antimicrobial Resistance at the University of Oxford, Oxford, United Kingdom; Nuffield Department of Medicine, University of Oxford, Oxford, United Kingdom; The National Institute for Health Research Health Protection Research Unit in Healthcare Associated Infections and Antimicrobial Resistance at the University of Oxford, Oxford, United Kingdom; Department of Infectious Diseases and Microbiology, Oxford University Hospitals NHS Foundation Trust, John Radcliffe Hospital, Oxford, United Kingdom; Big Data Institute, Nuffield Department of Population Health, University of Oxford, Oxford, United Kingdom; Department of Mathematics, University of Manchester, Manchester, United Kingdom; IBM Research, Hartree Centre, Sci-Tech Daresbury, United Kingdom; Office for National Statistics, Newport, United Kingdom; Office for National Statistics, Newport, United Kingdom; Office for National Statistics, Newport, United Kingdom; Office for National Statistics, Newport, United Kingdom; Office for National Statistics, Newport, United Kingdom; Nuffield Department of Medicine, University of Oxford, Oxford, United Kingdom; The National Institute for Health Research Health Protection Research Unit in Healthcare Associated Infections and Antimicrobial Resistance at the University of Oxford, Oxford, United Kingdom; The National Institute for Health Research Oxford Biomedical Research Centre, University of Oxford, Oxford, United Kingdom; Department of Infectious Diseases and Microbiology, Oxford University Hospitals NHS Foundation Trust, John Radcliffe Hospital, Oxford, United Kingdom; Nuffield Department of Medicine, University of Oxford, Oxford, United Kingdom; Department of Infectious Diseases and Microbiology, Oxford University Hospitals NHS Foundation Trust, John Radcliffe Hospital, Oxford, United Kingdom; Nuffield Department of Medicine, University of Oxford, Oxford, United Kingdom; The National Institute for Health Research Health Protection Research Unit in Healthcare Associated Infections and Antimicrobial Resistance at the University of Oxford, Oxford, United Kingdom; The National Institute for Health Research Oxford Biomedical Research Centre, University of Oxford, Oxford, United Kingdom; Department of Infectious Diseases and Microbiology, Oxford University Hospitals NHS Foundation Trust, John Radcliffe Hospital, Oxford, United Kingdom; Nuffield Department of Medicine, University of Oxford, Oxford, United Kingdom; The National Institute for Health Research Health Protection Research Unit in Healthcare Associated Infections and Antimicrobial Resistance at the University of Oxford, Oxford, United Kingdom; Department of Infectious Diseases and Microbiology, Oxford University Hospitals NHS Foundation Trust, John Radcliffe Hospital, Oxford, United Kingdom

**Keywords:** SARS-CoV-2, community, symptoms, testing

## Abstract

**Background:**

“Classic” symptoms (cough, fever, loss of taste/smell) prompt severe acute respiratory syndrome coronavirus 2 (SARS-CoV-2) polymerase chain reaction (PCR) testing in the United Kingdom. Studies have assessed the ability of different symptoms to identify infection, but few have compared symptoms over time (reflecting variants) and by vaccination status.

**Methods:**

Using the COVID-19 Infection Survey, sampling households across the United Kingdom, we compared symptoms in PCR-positives vs PCR-negatives, evaluating sensitivity of combinations of 12 symptoms (percentage symptomatic PCR-positives reporting specific symptoms) and tests per case (TPC) (PCR-positives or PCR-negatives reporting specific symptoms/ PCR-positives reporting specific symptoms).

**Results:**

Between April 2020 and August 2021, 27 869 SARS-CoV-2 PCR-positive episodes occurred in 27 692 participants (median 42 years), of whom 13 427 (48%) self-reported symptoms (“symptomatic PCR-positives”). The comparator comprised 3 806 692 test-negative visits (457 215 participants); 130 612 (3%) self-reported symptoms (“symptomatic PCR-negatives”). Symptom reporting in PCR-positives varied by age, sex, and ethnicity, and over time, reflecting changes in prevalence of viral variants, incidental changes (eg, seasonal pathogens (with sore throat increasing in PCR-positives and PCR-negatives from April 2021), schools reopening) and vaccination rollout. After May 2021 when Delta emerged, headache and fever substantially increased in PCR-positives, but not PCR-negatives. Sensitivity of symptom-based detection increased from 74% using “classic” symptoms, to 81% adding fatigue/weakness, and 90% including all 8 additional symptoms. However, this increased TPC from 4.6 to 5.3 to 8.7.

**Conclusions:**

Expanded symptom combinations may provide modest benefits for sensitivity of PCR-based case detection, but this will vary between settings and over time, and increases tests/case. Large-scale changes to targeted PCR-testing approaches require careful evaluation given substantial resource and infrastructure implications.

## Teaser

Within the COVID-19 Infection Survey, recruiting representative households across the UK general population, SARS-CoV-2-associated symptoms varied by viral variant, vaccination status and demographics. However, differences are modest and do not currently warrant large-scale changes to targeted testing approaches.

Symptomatic severe acute respiratory syndrome coronavirus 2 (SARS-CoV-2) infection is associated with higher viral loads [1], and higher viral loads with infectivity and transmission [2], although infections that remain asymptomatic [3, 4] have lower individual consequences. Resource constraints prevent universal testing, so testing strategies are usually targeted to the most predictive symptoms and/or contacts of known positives. Currently, 4 “classic” symptoms trigger polymerase chain reaction (PCR)-based community testing in the United Kingdom, loss/change of smell/taste, fever, and/or a new, continuous cough. In the United States, the Centers for Disease Control and Prevention (CDC) advises testing for any of fever or chills, cough, shortness of breath/difficulty breathing, new loss of taste/smell, fatigue, muscle or body aches, headache, sore throat, congestion/runny nose, nausea/vomiting, or diarrhea.

As testing policies depend on symptoms, understanding their predictive value in the context of seasonality, changing prevalence of different variants [1], and vaccination [5] is essential. Most studies to date have restricted to those hospitalized or seeking healthcare, who do not represent most infections [6]. Three recent UK community-based studies suggested that sensitivity could be increased by 10–20% by extending the “classic” symptoms. REACT [7] recommended adding headache, muscle aches, chills, and appetite loss, depending on age. ZOE [8] included different symptoms depending on age, sex, body mass index (BMI) and working in healthcare. VirusWatch [9] added feeling feverish, headache, muscle aches, loss of appetite or chills, but at a cost of increasing numbers eligible for testing 2- to 3-fold and tests per (symptomatic PCR-positive) case (TPC) to 7-fold. However, these studies were mainly before widespread vaccination, while Alpha dominated. Although ZOE found no evidence that symptoms varied between wild type and Alpha [10], with Delta, ZOE identified headache, sore throat, and runny nose/sneezing as nonclassic symptoms most commonly occurring in fully, partially, and unvaccinated PCR-positives [11].

We therefore used a large representative community-based UK survey to investigate symptoms over time in PCR-positives and PCR-negatives, also evaluating the impact of age, ethnicity, cycle threshold (Ct) value, vaccination status, and PCR gene profile (a proxy for variant).

## METHODS

The Office for National Statistics (ONS) COVID-19 Infection Survey [12] (ISRCTN21086382, https://www.ndm.ox.ac.uk/covid-19/covid-19-infection-survey/protocol-andinformation-sheets) continuously randomly selects private households from address lists and previous surveys. Having obtained verbal agreement, each household is visited by a study worker, and written informed consent obtained for individuals aged ≥2 years (from parents/carers for those 2–15 years, those 10–15 years providing written assent). At the first visit, participants may consent for optional follow-up visits every week for the next month, then monthly thereafter. At each visit, participants provide a nose and throat self-swab and answer questions about behaviors, work, vaccination uptake and 12 specific symptoms in the last 7 days (https://www.ndm.ox.ac.uk/covid-19/covid-19-infection-survey/case-record-forms): loss of taste, loss of smell, fever, cough, headache, tiredness/weakness (denoted fatigue/weakness), muscle ache (denoted muscle ache/myalgia), abdominal pain, diarrhea, nausea or vomiting, shortness of breath and sore throat; plus a general question about any symptoms participants considered coronavirus disease 2019 (COVID-19)-related (combined with specific symptoms as any evidence of symptoms).

Swabs are tested at national laboratories using the Thermo-Fisher TaqPath PCR assay (3 targets: ORF1ab, nucleocapsid [N], and spike protein [S]). If N and/or ORF1ab genes are detected, samples are called positive; the S-gene can accompany other genes, but does not count as positive alone.

We included the first positive study test in each PCR-positive “episode”, defining reinfections (arbitrarily) as occurring ≥120 days after an index positive with a preceding negative test, or after 4 consecutive negative tests [5]. Each positive episode was characterized by its minimum cycle threshold (Ct) value (reflecting maximum viral load) and by viral variant as wild-type/Delta if the S-gene was ever detected (by definition, with N/ORF1ab/both), as Alpha-compatible if positive at least once for ORF1ab+N, otherwise “other” (N-only/ORF1ab-only) (Supplementary Figure 1). Symptom presence included reports at any visit (PCR-positive/PCR-negative/failed) within [0, +35] days of the first PCR-positive.

The comparator was visits with negative PCR tests, excluding visits with symptoms related to ongoing COVID-19 (and long COVID), with high probability of undetected COVID-19, and where symptoms were likely associated with recent vaccination (Supplementary Methods, Supplementary Figure 2).

### Statistical Analyses

Primary analyses restricted to positive episodes vs negative visits with evidence of any symptoms (“symptomatic PCR-positives” vs “symptomatic PCR-negatives”), because this population is targeted for testing. We considered all symptomatic PCR-positives, and subgroups defined by Ct, viral variant, vaccination status, age, and calendar time (reflecting background incidental symptoms) (Supplementary Methods).

Initially, hierarchical clustering with complete linkage (Jaccard distance) assessed congruence of self-reported symptoms internally. To investigate reporting any symptom, and each symptom in symptomatic PCR-positives/PCR-negatives, we fitted generalized additive models (binomial distribution with complementary log-log link, mgcv [v.1.8-31] package), adjusting simultaneously for calendar time (smoothing spline), age (smoothing spline), sex, ethnicity (White vs non-White). We tested whether effects varied in PCR-positives vs PCR-negatives using interaction tests. In PCR-positives, separate models also adjusted for Ct value (smoothing spline) and viral variant, or vaccination status (Supplementary Methods). We fitted logistic regression with PRC-positivity as the outcome and the 12 symptoms as explanatory variables (Supplementary Methods).

We considered the 4 classic symptoms as “baseline” and assessed the impact of adding each of the other 8 symptoms, of every combination of 1–8 symptoms, and any of the individual 12 symptoms, on sensitivity for detecting symptomatic PCR-positives using standard metrics (Supplementary Methods, epiR [v.1.0-15], pROC [v.1.16.2] packages). We calculated tests per case (TPC) as 1/positive predictive value (PPV) (PCR-positives or PCR-negatives reporting specific symptoms/symptomatic PCR-positives reporting specific symptoms) and the inflation factor as PCR-positives or PCR-negatives reporting specific symptom/PCR-positives or PCR-negatives reporting classic symptoms. We compared symptoms reported at first vs subsequent visits within PCR-positive episodes (both absent, both present, absent then present, present then absent) and associated Ct distributions.

## RESULTS

Between 26 April 2020 and 7 August 2021, the study generated 5 130 318 PCR results from 484 317 participants; 34 494 (0.67%) were SARS-CoV-2-positive. In total, 27 869 PCR-positive episodes occurred (27 692 participants, median age 42 years [IQR 22–58]); self-reported symptoms were present in 13 427 (48%) (“symptomatic PCR-positives”). The comparator comprised 3 806 692 negative visits (457 215 participants, median age 52 years [IQR 32–66]); self-reported symptoms were present in 130 612 (3%) (“symptomatic PCR-negatives”) (exclusions in Supplementary Tables 1 and 2; characteristics and representativeness in Supplementary Tables 3–6).

### Specific Symptoms Are Associated With SARS-CoV-2 and Variants

**Figure 1.**
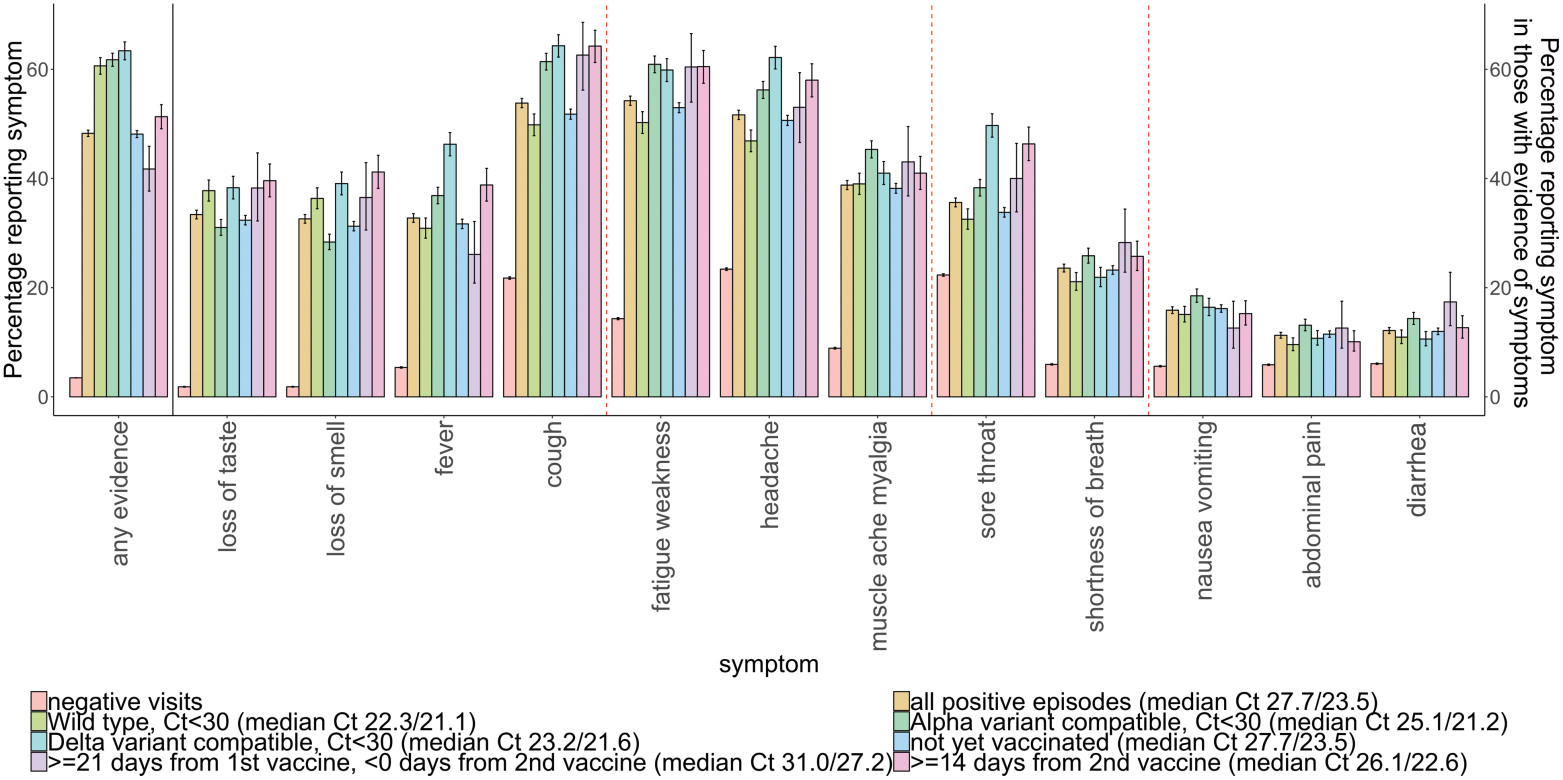
Percentage of individuals with and without SARS-CoV-2 infection self-reporting symptoms, presented as a proportion of all those reporting symptoms. Wild-type defined as S-gene positive before 17 November 2020; Alpha-compatible defined as S-gene negative from 17 November 2020 through 17 May 2021, Delta-compatible defined as S-gene positive from 17 May 2021. Post-vaccination positives split into not yet vaccinated, those 21 days after 1st vaccination and before 2nd vaccination, and 14 days or more after 2nd vaccination. Denominators will vary; for example, the absolute number of PCR-positives is much smaller 14 days or more post 2nd vaccination than unvaccinated. The 2 median values are median Ct in all and symptomatic PCR-positives in each group. See Figure 6 for associations between Ct and symptoms. Red dashed lines indicate symptom clusters based on hierarchical clustering (Supplementary Figure 3). See Figures 2–6 for adjusted analyses as unadjusted summaries do not control for confounding. Abbreviations: Ct, cycle threshold; PCR, polymerase chain reaction; SARS-CoV-2, severe acute respiratory syndrome coronavirus 2.

Fatigue/weakness, cough, and headache were the most frequently reported symptoms in PCR-positives (54%, 54%, 52% of symptomatic PCR-positives; Figure 1). However, headache and cough were also frequent among PCR-negatives, as was sore throat (23%, 22%, 22%, respectively; Figure 1). Loss of taste/smell were the most specific symptoms for SARS-CoV-2 positivity (reported in 33%/33% PCR-positives, vs 2%/2% PCR-negatives). In PCR-positives, loss of taste or smell were commonly co-reported, as were gastrointestinal symptoms, and headache/myalgia/fatigue. Symptom co-reporting in symptomatic PCR-positives was broadly similar regardless of Ct, variant, vaccination status or age (Supplementary Figure 3). Symptom clustering was broadly similar in PCR-negatives, except cough and sore throat were more commonly co-reported.

In symptomatic PCR-positives, symptomatology varied by viral variant (Figure 1; unadjusted). A smaller percentage of symptomatic PCR-positives reported loss of taste/smell for Alpha-compatible (31%/28%) than wild-type (38%/36%) or Delta-compatible (38%/39%) infection (*P* < .0001). Fever/headache/sore throat had the largest difference between symptoms reported for Alpha-compatible (37%/56%/38%) and Delta-compatible (46%/62%/50%) infection (*P* < .0001). Cough and fatigue/weakness had the largest differences between wild-type (50%/50%) vs Alpha-compatible (61%/61%) or Delta-compatible (64%/60%) infection (*P* < .0001). In general, specific symptoms were reported slightly more in symptomatic PCR-positives ≥14 days from 2nd vaccination versus those unvaccinated or ≥21 days from 1st vaccination (Figure 1).

### Symptomatology Over Calendar Time

**Figure 2.**
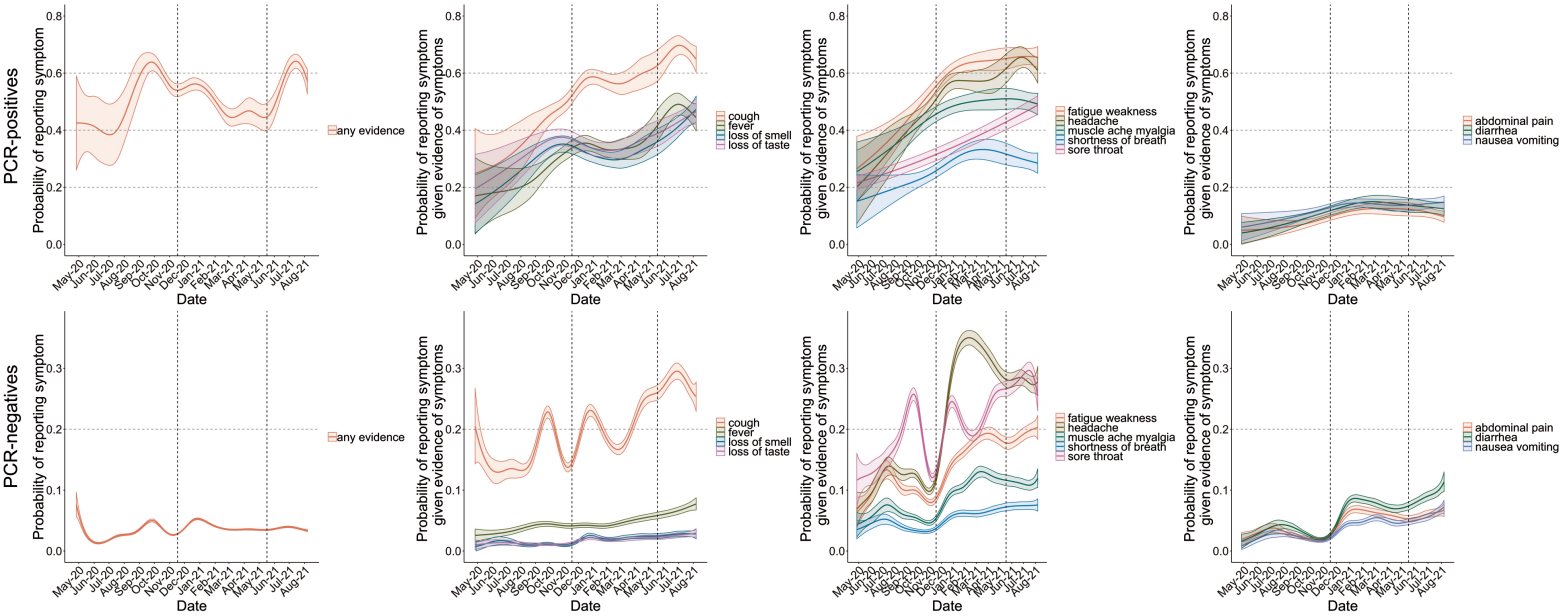
By calendar time, probability of reporting any evidence of symptoms (1st column), and specific classic symptoms (2nd column), systemic and respiratory symptoms (3rd column) and gastrointestinal symptoms (4th column) in those with evidence of symptoms, in SARS-CoV-2 PCR-positives (top row) and PCR-negatives (bottom row). Models adjusted for age, sex, ethnicity (presented at the reference category age 45, male, White). Top and bottom rows have different scales for the *y*-axis. Dashed lines at 17 November 2020 and 17 May 2021 indicate the emergence of Alpha and Delta, respectively, see Supplementary Figure 1. Abbreviations: PCR, polymerase chain reaction; SARS-CoV-2, severe acute respiratory syndrome coronavirus 2.

Adjusting for age, sex, and ethnicity, the probability of reporting any symptoms among PCR-positives was reasonably stable after August 2020 given changes in incidence and sample size (Figure 2, top panels, Supplementary Table 4), with fluctuations likely reflecting school return in September 2020 and March 2021, plus the emergence of Alpha and Delta in November 2020 and May 2021. Smaller fluctuations in reporting any symptoms in PCR-negatives (Figure 2, bottom panels) mirrored those in PCR-positives. The percentage of symptomatic PCR-positives reporting each specific symptom generally increased over time, consistent with increasing awareness. Reporting of most specific symptoms except loss of taste/smell temporarily peaked in January 2021, consistent with the peak in Alpha, then remained approximately constant through May 2021, before increasing again, markedly so for headache, cough, and fever after Delta became dominant. Increases in cough and sore throat in symptomatic PCR-negatives were consistent with spread of other respiratory viruses, particularly after school return in September 2020, in January 2021 when transmission events may have been linked to Christmas, and from early April 2021 when schools had reopened [13]. The winter months saw particular increases in gastrointestinal symptoms, fatigue/weakness, myalgia, and headache in symptomatic PCR-negatives, consistent with the presence of other seasonal pathogens.

### Symptomatology by Age, Sex, and Ethnicity

**Figure 3.**
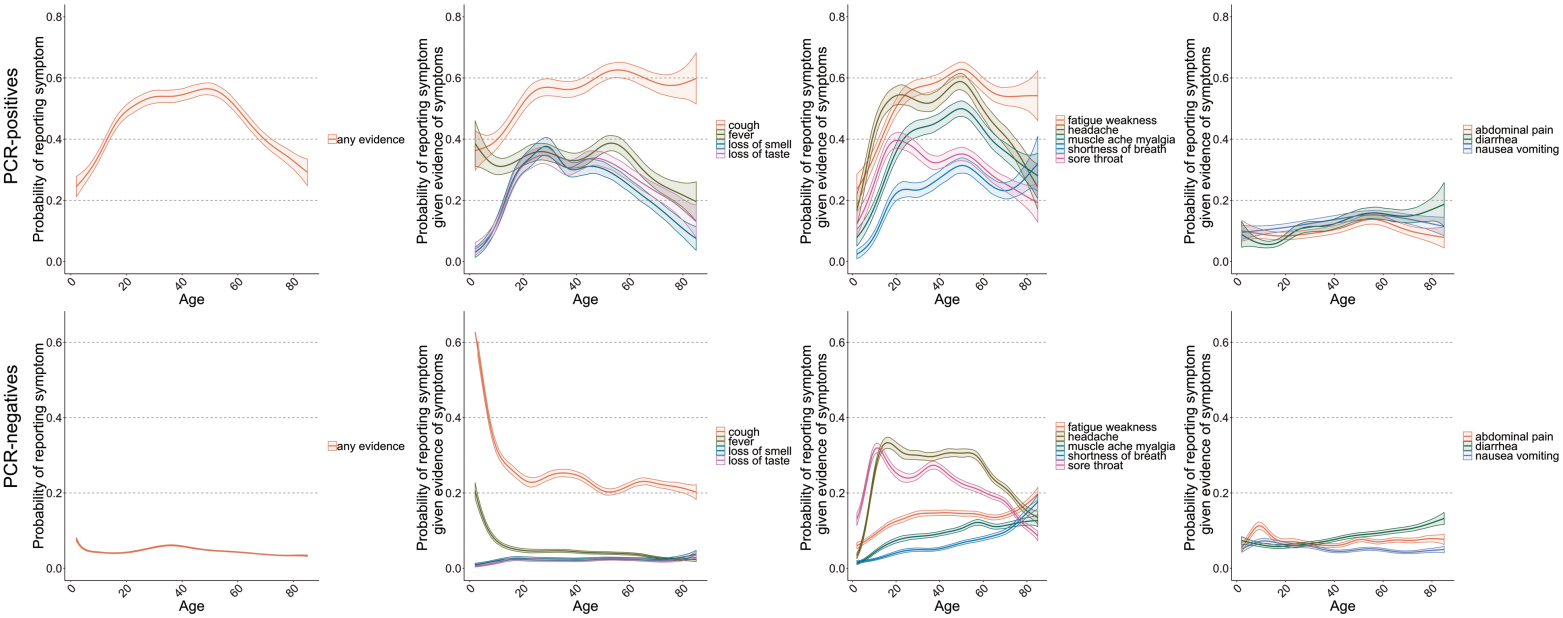
By age, probability of reporting any evidence of symptoms (1st column), specific classic symptoms (2nd column), systemic and respiratory symptoms (3rd column), and gastrointestinal symptoms (4th column) in those with evidence of symptoms, in positives (top row) and PCR-negatives (bottom row). Models adjusted for calendar date, sex, ethnicity (reference category 1 January 2021, male, White). Top and bottom rows have different scales for the *y*-axis. Abbreviation: PCR, polymerase chain reaction.

All symptoms showed marked variation across age in both PCR-positives and PCR-negatives, typically being reported less by children and elderly adults (Figure 3). Loss of taste/smell were most frequently reported in symptomatic PCR-positives in those aged ~20 years, decreasing gradually with age, and were rare in symptomatic PCR-negatives, consistent with their high specificity for SARS-CoV-2. Sore throat and headache were most frequently reported in late adolescence, irrespective of SARS-CoV-2 positivity. Cough was common and reported similarly in both symptomatic PCR-positives and PCR-negatives to ~10 years. However, above 20 years the proportion reporting cough in symptomatic PCR-positives was more than double that in PCR-negatives, and increased to ~60 years. Similar patterns were observed for fatigue/weakness, shortness of breath, and diarrhea.

**Figure 4.**
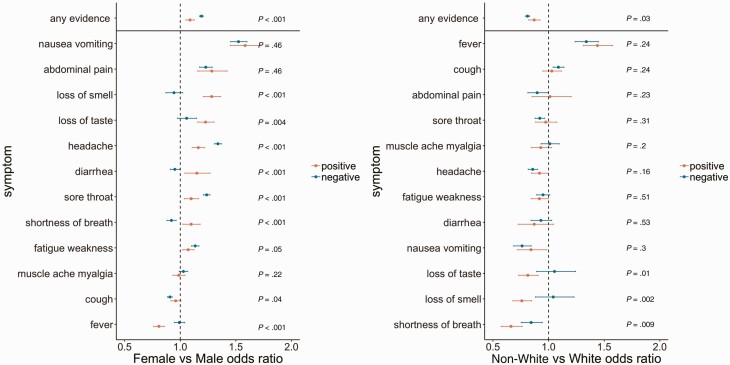
Odds ratios (95% CI) by sex and ethnicity of reporting any evidence of symptoms, as well as each of 12 specific symptoms in those with evidence of symptoms, comparing SARS-CoV-2 PCR-positives (*red*) and PCR-negatives (*turquoise*). Data are shown by sex (female vs male, left), and ethnicity (non-White vs White, right). *P*-values are heterogeneity tests between the effects of sex and ethnicity on reporting symptoms in PCR-positives vs PCR-negatives. or Models adjusted for calendar date (Figure 2), age (Figure 3), sex, and ethnicity. Where 95% CI cross 1, there is no evidence that sex/ethnicity affects the odds of reporting that symptom given evidence of symptoms in PCR-positives/PCR-negatives. Where there is evidence of heterogeneity, there is a different effect of sex/ethnicity on reporting the symptom in PCR-positives vs PCR-negatives. Abbreviations: CI, confidence interval; PCR, polymerase chain reaction.

Adjusting for calendar time, age, and ethnicity, women were more likely than men to report most symptoms (Figure 4). Increased reporting in women was significantly greater in symptomatic PCR-positives than PCR-negatives for loss of smell and taste, diarrhea, and shortness of breath, and significantly smaller for headache and sore throat (all heterogeneity *P* < .01). In symptomatic PCR-positives, females were significantly less likely to report fever than males, whereas in PCR-negatives there was no evidence of difference in reporting fever between males and females (heterogeneity *P* < .001).

After adjusting for calendar time, age, and sex, non-White ethnic groups were more likely to report fever than White ethnic groups and less likely to report headache, nausea/vomiting, and shortness of breath in both symptomatic PCR-positives and PCR-negatives (Figure 4). In symptomatic PCR-positives, those from non-White ethnic groups were significantly less likely to report loss of taste/smell and shortness of breath than White ethnic groups, whereas in PCR-negatives there was no evidence of differences for loss of taste/smell, and much smaller differences for shortness of breath (heterogeneity *P* < .01).

### Symptoms by Vaccination Status

**Figure 5.**
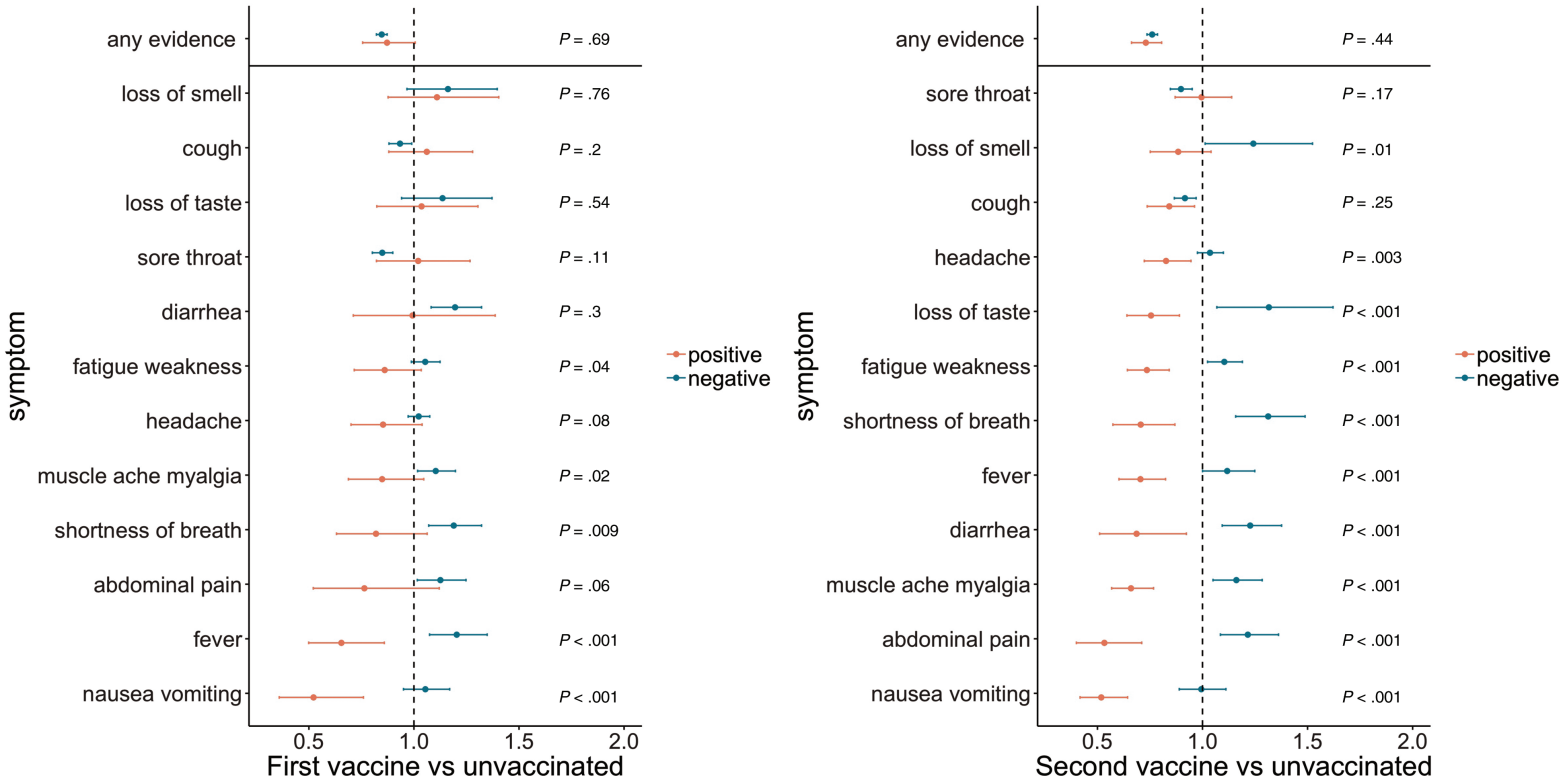
Odds ratios (95% CI) by vaccination status of reporting any evidence of symptoms, as well as each of 12 specific symptoms in those with evidence of symptoms, comparing SARS-CoV-2 PCR-positives (*red*) and PCR-negatives (*turquoise*). Data are shown by vaccination status (≥21 days from 1st vaccine and before 2nd vaccine vs pre-vaccination, left, and ≥14 days from 2nd vs pre-vaccination, right). p-values are heterogeneity tests between the effects of vaccination on reporting symptoms in PCR-positives vs PCR-negatives. Note: models adjusted for calendar date (Figure 2), age (Figure 3), sex (Figure 4) and ethnicity (Figure 5). Where 95% CI cross 1, there is no evidence that vaccination status affects the odds of reporting that symptom given evidence of symptoms in PCR-positives/PCR-negatives. Where there is evidence of heterogeneity, there is a different effect of vaccination status on reporting the symptom in PCR-positives vs PCR-negatives. Abbreviations: CI, confidence interval; PCR, polymerase chain reaction; SARS-CoV-2, severe acute respiratory syndrome coronavirus 2.

In adjusted analyses, any symptoms were reported less frequently in those single or double vaccinated vs unvaccinated, similarly in PCR-positives and PCR-negatives (Figure 5, heterogeneity *P* > .44). In PCR-positives, 10 of 12 symptoms were less frequently reported in those double vaccinated than unvaccinated (Figure 5); but 7 of these were more frequently reported in double vaccinated than unvaccinated PCR-negatives.

### Symptoms by Ct Value in PCR-Positives

**Figure 6.**
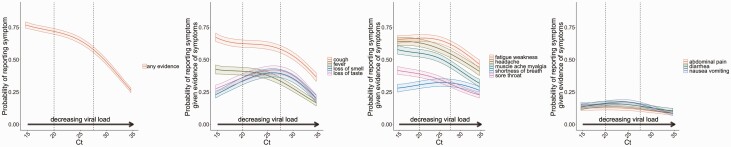
By Ct values, probability of self-reported symptoms in individuals with PCR-confirmed SARS-CoV-2 infection. First column: any symptoms; 2nd, 3rd, 4th columns: each of the 12 specific symptoms in those who reported any symptom(s). Models adjusted for calendar date (Figure 2), age (Figure 3), sex, and ethnicity (Figure 4) (reference category 1 January 2021, 45, male, White). See Supplementary Figure 4 for models also adjusting for S-gene positivity pattern with similar results. Abbreviations: Ct, cycle threshold; PCR, polymerase chain reaction; SARS-CoV-2, severe acute respiratory syndrome coronavirus 2.

At low Ct values (≤20; high viral load), the most commonly reported symptoms were cough, fatigue/weakness, headache, and muscle ache/myalgia, occurring in >50% of symptomatic PCR-positives (Figure 6; adjusted for viral variant in Supplementary Figure 4). Above Ct >27.5 (low viral load), all symptoms declined in prevalence with a trajectory that tracked Ct; between Ct 20 and 27.5, most symptoms showed little variation. Interestingly, reported loss of taste/smell increased substantially from ~30% to ~45% between Ct 15 and 27.5, with smaller increases for shortness of breath. These symptoms may occur later in infection, hence the apparently inverse relationship between viral load and symptom prevalence.

### Symptom Combinations Predicting Symptomatic PCR-Positives

Over the whole study, PPV of any symptoms for identifying PCR-positives was 9%. Among symptomatic PCR-positives, 74% reported any of the 4 classic symptoms, requiring 4.6 TPC. Including any of the 12 elicited symptoms maximized sensitivity (90% of symptomatic PCR-positives reported at least 1 specific symptom, remainder only reporting generic other “COVID-19-related” symptoms, see Methods). However, recommending PCR tests on this basis would increase TPC to 8.7 and number of tests 2.3-fold compared with 4 classic symptoms only (Supplementary Table 7, Supplementary Results).

For a fixed number of 1–8 symptoms, choosing whether to maximize sensitivity or area under the receiver operating characteristic (AUROC) curve, or to minimize TPC or inflation factor, led to different optimal combinations (Supplementary Table 7). However, these frequently included 1 or more of the classic 4 symptoms. Sensitivity was generally higher for combinations including fatigue/weakness and/or headache, but these resulted in higher TPC, particularly for headache. Including gastrointestinal symptoms had the lowest TPC but also lowest sensitivity. In those double vaccinated, sore throat had similar effects on sensitivity and TPC as headache, and in children diarrhea had greater benefits for sensitivity (Supplementary Table 7).

**Figure 7.**
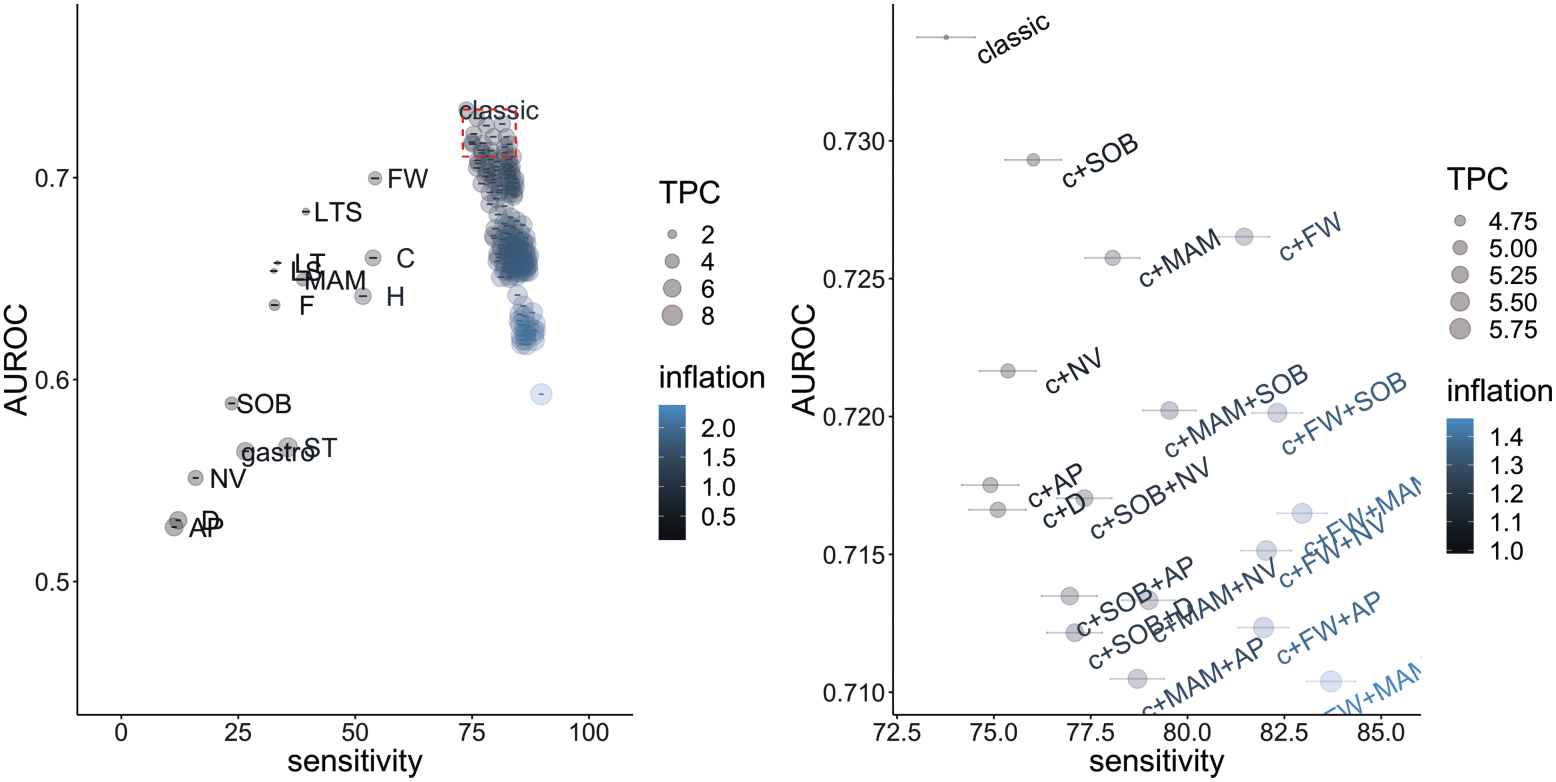
Performance of individual symptoms, as well as the classic 4 symptoms (cough, fever, loss of taste/smell), classic plus all possible combinations of 1/2/3/4 symptoms, and any of the 12 named symptoms, in predicting SARS-CoV-2 positivity in those with evidence of symptoms in terms of sensitivity and overall accuracy (AUROC). For exact values, see Supplementary Table 8. Right-hand panel is an expanded version of the top right corner of the left panel (red box, AUROC >90th quantile, sensitivity >sensitivity of combination of classic 4 symptoms). Inflation (relative numbers reporting these symptoms compared to classic symptoms) and TPC are also included in the visualization. TPC = 1/positive predictive value. By definition, as the number of symptoms increases, sensitivity also increases. Abbreviations: AP, abdominal pain; c, classic; C, cough; D, diarrhea; F, fever; FW, weakness/tiredness; H, headache; LS, loss of smell; LT, loss of taste; LTS, loss of taste or smell; AM, muscle ache/myalgia; NV, nausea/vomiting; SARS-CoV-2, severe acute respiratory syndrome coronavirus 2; SOB, shortness of breath; ST, sore throat; TPC, tests per case.

Balancing different performance metrics, adding fatigue/weakness to the classic 4 symptoms improved sensitivity from 74% to 81%, although dropping AUROC by <0.01 (0.734 to 0.727) (Figure 7). However, TPC increased from 4.6 to 5.3, and 1.3 times more people would need testing. This combination generally performed well across subgroups (Supplementary Table 8). Adding other symptoms to the classic 4 symptoms generally led to lower AUROCs and, at best, similar sensitivity (Supplementary Table 8, Figure 7), excepting children/adolescents in whom adding headache achieved highest sensitivities when considering adding only one extra symptom, and also highest AUROC for those aged under 10 years (Supplementary Figures 5–9). In a confirmatory logistic regression, associations with positivity were strongest for reporting loss of smell or taste, and then fever, fatigue weakness, cough, muscle ache myalgia, headache, and shortness of breath (Supplementary Figure 10).

### Symptomatology at Different Stages of Infection

Considering symptoms over time in PCR-positive episodes with ≥2 visits within 35 days (Supplementary Table 9), the most common symptoms presenting after the index positive were fatigue/weakness (8%), headache (7%), cough (6%), loss of taste (6%), loss of smell (5%), or muscle ache/myalgia (5%) (Supplementary Table 10). For most symptoms, Ct values were highest in those never reporting the symptom, lowest in those reporting it initially and subsequently, and intermediate where symptoms were reported at either the initial or subsequent visits only (Supplementary Figure 11). The main contrast was loss of taste and loss of smell, where Ct values were lowest in those reporting loss of taste or smell at subsequent visits only (*P* < .0001).

## DISCUSSION

Here, we investigated the performance of symptom-based approaches to PCR testing for SARS-CoV-2 using a large community-based UK survey. Notably, reporting of any symptoms in SARS-CoV-2 infections varied substantially over calendar time (40–70%), reflecting changing dominance of specific variants (Alpha, Delta), positivity rates (higher viral burden symptomatic infections being identified more frequently when incidence is increasing [5]), and background incidental changes (eg, public awareness of SARS-CoV-2-associated symptoms, seasonal pathogens, schools reopening). Symptom reporting in PCR-positives vs PCR-negatives varied by age, sex, ethnicity, and vaccination status. This variability highlights the importance of considering local context when developing symptom-based screening strategies. Broadly, in our setting, of the 12 symptoms evaluated, the 4 classic symptoms gave close to optimal symptom-based screening performance given limited testing capacity. Where additional testing capacity is available, adding fatigue/weakness improved sensitivity most (+7%) whilst inflating TPC by only 15%. Lateral flow antigen tests (LTFs) add a further dimension to testing strategies, not evaluated here. However, LFT sensitivity for detecting symptomatic cases is lower than PCR [14], reflected in the current guidance for LFT use for asymptomatic cases only.

Although the CDC approach of using a broad range of symptoms to prompt testing maximizes sensitivity of case detection, it has substantially higher TPC (8.7 vs 4.6 for classic symptoms) and total tests (2.3-fold) with associated costs and capacity requirements. The UK approach, focusing on 4 classic symptoms, has lower sensitivity (74% vs 90%) but higher accuracy to detect symptomatic infection overall (AUROC 0.734 vs 0.593). Increased sensitivity from adding symptoms to the 4 classic symptoms typically reduced overall accuracy and/or increased TPC and tests needed, highlighting the importance of evaluating several test metrics. Advantages from including additional symptoms were limited, but those that best improved sensitivity across multiple subgroups included fatigue/weakness or muscle ache/myalgia, or, in children/adolescents, headache. The REACT study [7] evaluating symptom constellations during the Alpha wave (December 2020/January 2021) suggested adding headache, muscle aches, chills, and appetite loss to the classic symptoms. Our survey did not specifically elicit chills or appetite loss; however, we found headache had poorer specificity, being commonly reported in PCR-negatives, particularly adults, leading to substantially increased TPC. To optimize sensitivity, REACT proposed different symptom combinations for adults and children, requiring careful public health messaging. ZOE [8] suggested an algorithm also including working in healthcare; although this could theoretically be programmed into an online test system, such complexity risks gaming the system if individuals cannot otherwise access tests.

The main limitation is that the survey collected only 12 specific symptoms, plus 1 generic question, to minimize participant burden. We therefore could not evaluate some symptoms more recently proposed for inclusion, such as coryza [15, 16]. Parents/caregivers reported symptoms for children; symptom reporting may be affected by other cultural differences we could not adjust for, and by public awareness (eg, increased reporting of loss of taste/smell once this became recognized). Power was limited within some subgroups, for example, children and specific non-White ethnic groups. The survey does not include those in care homes or hospitalized with severe disease who may have different symptom profiles. Although the survey randomly and continuously selects households from address lists, and survey participants broadly reflect the wider population (Supplementary Tables 3–6), they are slightly older and more likely to report White ethnicity. Testing was predominantly monthly; although individuals were followed longitudinally, we had limited resolution to assess the short-term evolution of symptoms during infection. Virus Watch showed that fever and loss of taste/smell occurred later in the disease course [9], with similar findings for fever in ZOE [17]. However, they were all still chosen in the top performing symptoms for screening, suggesting this may have limited impact.

The main study strengths are its size and population representativeness, particularly capturing mild infections in the community. We took a stringent approach to defining our “PCR-negative” comparator to limit possible contamination from undetected infections/ongoing COVID-19. We report over periods that include different dominant viral variants. Rather than optimizing individual criteria, we compared predictive performance taking into account trade-offs between overall accuracy, sensitivity and TPC over different background prevalences, reflecting practical concerns regarding testing capacity.

Overall, given performance trade-offs, we did not find any major shift away from the importance of the classic 4 symptoms in PCR-positives, despite changes associated with the emergence of Delta and vaccine rollout. Given their concurrent changes in PCR-negatives, recent reports of associations with sore throat may reflect background increases in other respiratory infections/hay fever, potentially even with SARS-CoV-2 isolated incidentally given that one-third of cases are plausibly asymptomatic [4]. Currently, we therefore have limited evidence for expanding the case definition beyond the classic 4 symptoms where universal PCR testing is not practical/affordable. However, this requires ongoing monitoring as other respiratory viruses increasingly circulate following lifting of restrictions with vaccine rollout [18–21], potentially altering the specificity of symptoms in determining SARS-CoV-2 vs other community-acquired infections.

## Supplementary Data

Supplementary materials are available at *Clinical Infectious Diseases* online. Consisting of data provided by the authors to benefit the reader, the posted materials are not copyedited and are the sole responsibility of the authors, so questions or comments should be addressed to the corresponding author.

ciab945_suppl_Supplementary_Materials

## Notes

***Acknowledgments.*** The authors are grateful for the support of all COVID-19 Infection Survey participants. Office for National Statistics: Sir Ian Diamond, Emma Rourke, Ruth Studley, Tina Thomas. Office for National Statistics COVID-19 Infection Survey Analysis and Operations teams, in particular: Daniel Ayoubkhani, Russell Black, Antonio Felton, Megan Crees, Joel Jones, Lina Lloyd, Esther Sunderland. University of Oxford, Nuffield Department of Medicine: Ann Sarah Walker, Derrick Crook, Philippa C Matthews, Tim Peto, Emma Pritchard, Nicole Stoesser, Karina-Doris Vihta, Jia Wei, Alison Howarth, George Doherty, James Kavanagh, Kevin K Chau, Sarah Cameron, Phoebe Tamblin-Hopper, Magda Wolna, Rachael Brown, Stephanie B Hatch, Daniel Ebner, Lucas Martins Ferreira, Thomas Christott, Brian D Marsden, Wanwisa Dejnirattisai, Juthathip Mongkolsapaya, Sarah Hoosdally, Richard Cornall, David I Stuart, E Yvonne Jones, Gavin Screaton. University of Oxford, Nuffield Department of Population Health: Koen Pouwels. University of Oxford, Big Data Institute: David W Eyre, Katrina Lythgoe, David Bonsall, Tanya Golubchik, Helen Fryer. University of Oxford, Radcliffe Department of Medicine: John Bell. University of Manchester: Thomas House. Public Health England: John Newton, Julie Robotham, Paul Birrell. IQVIA: Helena Jordan, Tim Sheppard, Graham Athey, Dan Moody, Leigh Curry, Pamela Brereton. Glasgow Lighthouse Laboratory: Jodie Hay, Harper VanSteenhouse. National Biocentre: Anna Godsmark, George Morris, Bobby Mallick, Phil Eeles. Oxford University Hospitals NHS Foundation Trust: Stuart Cox, Kevin Paddon, Tim James, Sarah Cameron, Phoebe Tamblin-Hopper, Magda Wolna, Rachael Brown. Department of Health: Jessica Lee.

***Contributors.*** This specific analysis was designed by A. S. W., K. B. P., P. C. M., N. S., D. W. E., T. H., D. C., T. E. A. P., and K.-D. V. In addition, K.-D. V. conducted the statistical analysis of the survey data. K.-D. V., N. S., P. C. M., and A. S. W. drafted the manuscript. All authors contributed to interpretation of the study results, and revised and approved the manuscript for intellectual content. K.-D. V. is the guarantor and accepts full responsibility for the work and conduct of the study, had access to the data, and controlled the decision to publish. The corresponding author (K.-D. V.) attests that all listed authors meet authorship criteria and that no others meeting the criteria have been omitted.

***Ethical approval.*** The study received ethical approval from the South Central Berkshire B Research Ethics Committee (20/SC/0195).

***Data sharing.*** Data are still being collected for the COVID-19 Infection Survey. De-identified study data are available for access by accredited researchers in the ONS Secure Research Service (SRS) for accredited research purposes under part 5, chapter 5 of the Digital Economy Act 2017. For further information about accreditation, contact Research.Support@ons.gov.uk or visit the SRS website.

***Transparency.*** The lead authors affirm that the manuscript is an honest, accurate, and transparent account of the study design being reported, no important aspects of the study have been omitted, and any discrepancies from the study as originally planned (and, if relevant, registered) have been explained. Dissemination to participants and related patient and public communities: Results of individual tests were communicated to the participants. Overall study results were disseminated through the preprint of the study. Findings were disseminated in lay language in the national and local press.

***Financial support.*** This study is funded by the Department of Health and Social Care with in-kind support from the Welsh Government, the Department of Health on behalf of the Northern Ireland Government and the Scottish Government. K.-D. V., K. B. P., A. S. W., T. E. A. P., N. S., and D. E. are supported by the National Institute for Health Research Health Protection Research Unit (NIHR HPRU) in Healthcare Associated Infections and Antimicrobial Resistance at the University of Oxford in partnership with Public Health England (PHE) (grant number NIHR200915). A. S. W. and T. E. A. P. are also supported by the NIHR Oxford Biomedical Research Centre. K. B. P. is also supported by the Huo Family Foundation. A. S. W. is also supported by core support from the Medical Research Council UK to the MRC Clinical Trials Unit (grant number MC_UU_12023/22) and is an NIHR Senior Investigator. P. C. M. is funded by Wellcome (intermediate fellowship, grant number 110110/Z/15/Z) and holds an NIHR Oxford BRC Senior Fellowship award. D. W. E. is supported by a Robertson Fellowship and an NIHR Oxford BRC Senior Fellowship. N. S. is an Oxford Martin Fellow and an NIHR Oxford BRC Senior Fellow. The views expressed are those of the authors and not necessarily those of the National Health Service, NIHR, Department of Health, or PHE. The funder/sponsor did not have any role in the design and conduct of the study; collection, management, analysis, and interpretation of the data; preparation, review, or approval of the manuscript; and decision to submit the manuscript for publication. All authors had full access to all data analysis outputs (reports and tables) and take responsibility for their integrity and accuracy.

For the purpose of Open Access, the author has applied a CC BY public copyright license to any Author Accepted Manuscript version arising from this submission.

***Potential conflicts of interest.*** D. W. E. declares lecture fees from Gilead outside the submitted work. All other authors report no potential conflicts. All authors have submitted the ICMJE Form for Disclosure of Potential Conflicts of Interest. Conflicts that the editors consider relevant to the content of the manuscript have been disclosed.

## References

[1] Walker AS , Pritchard E, House T, et al Ct threshold values, a proxy for viral load in community SARS-CoV-2 cases, demonstrate wide variation across populations and over time. Elife 2021; 10:1–18. 10.7554/eLife.64683 PMC8282332 34250907

[2] Lee LYW , Rozmanowski S, Pang M, et al Severe acute respiratory syndrome coronavirus 2 (SARS-CoV-2) infectivity by viral load, s gene variants and demographic factors, and the utility of lateral flow devices to prevent transmission. Clin Infect Dis 2021:1–9. Available at: https://academic.oup.com/cid/advance-article/doi/10.1093/cid/ciab421/6273394 10.1093/cid/ciab421 PMC8136027 33972994

[3] Syangtan G , Bista S, Dawadi P, et al Asymptomatic SARS-CoV-2 carriers: a systematic review and meta-analysis. Front Public Heal 2021; 8:1–10. 10.3389/fpubh.2020.587374 PMC7855302 33553089

[4] Sah P , Fitzpatrick MC, Zimmer CF, Abdollahi E, Juden-kelly L. Asymptomatic SARS-CoV-2 infection: a systematic review and meta-analysis. Proc Natl Acad Sci USA 2021; 118:1–12. 10.1073/pnas.2109229118 PMC8403749 34376550

[5] Pritchard E , Matthews PC, Stoesser N, et al Impact of vaccination on new SARS-CoV-2 infections in the United Kingdom. Nat Med 2021. doi:10.1038/s41591-021-01410-w. PMC8363500 34108716

[6] Chan AT , Brownstein JS. Putting the public back in public health—surveying symptoms of Covid-19. N Engl J Med 2020; 383:e45. 32501663 10.1056/NEJMp2016259

[7] Elliott J , Whitaker M, Bodinier B, et al Predictive symptoms for COVID-19 in the community: REACT-1 study of over 1 million people. PLoS Med 2021; 18:1–14. doi:10.1371/journal.pmed.1003777 10.1371/journal.pmed.1003777 PMC8478234 34582457

[8] Canas LS , Sudre CH, Capdevila Pujol J, et al Early detection of COVID-19 in the UK using self-reported symptoms: a large-scale, prospective, epidemiological surveillance study. Lancet Digit Heal 2021; 7500:1–12. 10.1016/S2589-7500(21)00131-X PMC8321433 34334333

[9] Fragaszy E , Shrotri M, Geismar C, et al Symptom profiles and accuracy of clinical definitions for COVID-19 in the community. Results of the virus watch community cohort. medRxiv 2021. Available at: http://medrxiv.org/content/early/2021/05/18/2021.05.14.21257229.abstract. 10.12688/wellcomeopenres.17479.1 PMC10514573 37745779

[10] Graham MS , Sudre CH, May A, et al Changes in symptomatology, reinfection, and transmissibility associated with the SARS-CoV-2 variant B.1.1.7: an ecological study. Lancet Public Heal 2021; 6:e335–45. 10.1016/S2468-2667(21)00055-4 PMC8041365 33857453

[11] ZOE Covid Study. What are the new top 5 COVID symptoms? 2021. Available at: https://covid.joinzoe.com/post/new-top-5-covid-symptoms. Accessed 9 August 2021.

[12] Pouwels KB , House T, Pritchard E, et al Community prevalence of SARS-CoV-2 in England from April to November 2020: results from the ONS coronavirus infection survey. Lancet Public Heal 2021; 6:e30–8. 10.1016/S2468-2667(20)30282-6 PMC7786000 33308423

[13] Lumley SF , Richens N, Lees E, et al Changes in paediatric respiratory infections at a UK teaching hospital 2016–2021; impact of the SARS-CoV-2 pandemic. J Infect. 2021; 1–8. doi:10.1016/j.jinf.2021.10.022 10.1016/j.jinf.2021.10.022 PMC8591975 34757137

[14] Petersen I , Crozier A, Buchan I, Mina MJ, Bartlett JW. Recalibrating SARS-CoV-2 antigen rapid lateral flow test relative sensitivity from validation studies to absolute sensitivity for detecting individuals with live virus. medRxiv 2021. Available at: http://medrxiv.org/content/early/2021/03/24/2021.03.19.21253922.abstract. 10.2147/CLEP.S311977 PMC8527245 34703318

[15] Crozier A , Dunning J, Rajan S, Semple MG, Buchan IE. Could expanding the covid-19 case definition improve the UK’s pandemic response? BMJ 2021; 374:17–20. 10.1136/bmj.n1625 34193527

[16] Sohal A. Open letter to Chris Whitty and Susan Hopkins: change covid-19 case definition in line with WHO to save lives. BMJ 2021; 372:2021. 10.1136/bmj.n283 33514502

[17] Antonelli M , Capdevila J, Chaudhari A, et al Optimal symptom combinations to aid COVID-19 case identification: analysis from a community-based, prospective, observational cohort. J Infect 2021; 82:384–90. 33592254 10.1016/j.jinf.2021.02.015 PMC7881291

[18] Huang QS , Wood T, Jelley L, et al Impact of the COVID-19 nonpharmaceutical interventions on influenza and other respiratory viral infections in New Zealand. Nat Commun 2021; 12:1–7. 33579926 10.1038/s41467-021-21157-9 PMC7881137

[19] Britton PN , Hu N, Saravanos G, et al COVID-19 public health measures and respiratory syncytial virus. Lancet Child Adolesc Heal 2020; 4:e42–3. 10.1016/S2352-4642(20)30307-2 PMC7500894 32956616

[20] Sherman AC , Babiker A, Sieben AJ, et al The effect of severe acute respiratory syndrome coronavirus 2 (SARS-CoV-2) mitigation strategies on seasonal respiratory viruses: a tale of 2 large metropolitan centers in the United States. Clin Infect Dis 2021; 72:E154–7. 33161424 10.1093/cid/ciaa1704 PMC7717225

[21] Park S , Michelow IC, Choe YJ. Shifting patterns of respiratory virus activity following social distancing measures for coronavirus disease 2019 in South Korea. J Infect Dis 2021:1–7. Available at: https://pubmed.ncbi.nlm.nih.gov/34009376/10.1093/infdis/jiab231 PMC8135809 34009376

