# Supplementary material for: Symptoms and Severe Acute Respiratory Syndrome Coronavirus 2 (SARS-CoV-2) Positivity in the General Population in the United Kingdom
Source: Clin Infect Dis. 2021 Nov 8;75(1):e329–37. doi: 10.1093/cid/ciab945 (PMC8767848; doi:10.1093/cid/ciab945)
Supplement: ciab945_suppl_Supplementary_Materials [file ciab945_suppl_supplementary_materials.docx]

# Supplementary Material

# Supplementary Methods

The presence of three SARS-CoV-2 genes (ORF1ab, N, S) was identified using real-time polymerase chain reaction (RT-PCR) with the TaqPath RT-PCR COVID-19 kit (Thermo Fisher Scientific, Waltham, MA, USA), analysed using UgenTec Fast Finder 3.300.5 (TaqMan 2019-nCoV assay kit V2 UK NHS ABI 7500 v2.1; UgenTec, Hasselt, Belgium).

### Choice of negative visits in the comparator group

As a comparator group, we initially included all visits where PCR tests were negative, and then excluded visits where symptoms could plausibly be related to ongoing effects of COVID-19 or long COVID, where there was a high pre-test probability that the participant actually had a new COVID-19 infection that had not been detected in the survey, or where symptoms were likely driven by recent vaccination. Specifically, we excluded all negative visits (numbers in **Table S1**):

1. **From -90 days before** the first S-antibody positive blood test in the study prior to vaccination, where such antibody results are likely to represent previous undetected infection (these results were available only in a random subset of the population);
2. **From -35 days before** the first swab positive onwards from individuals who ever tested PCR positive in the study or positive on either PCR or LFD in the linked English testing programme (to avoid ongoing long COVID symptoms,[1] and COVID-related symptoms occurring shortly before the positive test);
3. **From -35 days before** any self-reported positive swab test result onwards (for the same reason; reflecting the fact that individuals may have obtained tests elsewhere)
4. From a small number of individuals who reported either loss of taste or loss of smell at their first study visit and had no national testing programme result within [-21,+21] days (all before 1 July 2020), given the high specificity of this symptom for COVID-19 infection, the fact that it would have been impossible for these individuals to get an external test at the time and the potential for subsequent symptoms to represent long COVID;
5. Where participants reported self-isolating OR contact with **definite** positives in the preceding 28 days (since these individuals have much higher risk of SARS-CoV-2 infection which may not have been detected) and the **previous and the next visit** (because of higher risk of unidentified positivity, and because they may have been contact traced through the national training programme they may be more likely to report symptoms through recall bias, regardless of status);
6. Occurring within [-7,+14 days] of either first or second vaccination date[2], to avoid the inclusion of common symptoms caused by vaccination in the test-negative comparator group and to reflect the possibility of small inaccuracies in reported date of vaccination for some participants.

Time windows were arbitrary but aligned with other analyses or windows for considering symptoms associated with PCR-positive episodes.

### Choice of timeframe to include symptoms in the PCR-positive group

Tests are conducted in the survey independently of symptoms, and therefore infection episodes may be identified either early (pre-symptomatic) or late (post-symptomatic). Symptom questions relate to the previous 7 days, so to ensure that subsequently reported symptoms in pre-symptomatic cases were counted we included all symptoms reported at any visit up to 35 days after the index positive test, reflecting the monthly visit schedule. 29% of PCR-positive episodes had only one visit in the first 35 days after the index positive, with a further 50% having only two visits during this time (**Table S9**). Post-symptomatic PCR-positives (never reporting symptoms due to late study test compared with infection dates) were excluded, as analysis only included symptomatic PCR-positives.

### Subgroups

In order to assess the impact of various changes over the course of the epidemic, we considered symptoms overall in all PCR-positives, and in specific subgroups, as follows:

- 1. S-gene present before 17/Nov/2020 (wild-type) vs S-gene absent from 17/Nov/2020 to 17/May/2020 (Alpha-compatible) vs S-gene present from 17/May/2020 onwards (Delta-compatible) (**Fig.S1**); all restricted to Ct<30 to increase the chance that the survey positive test was closer to the start of the infection
  2. Ct<30 or ≥30 as a proxy for higher viral load (where symptoms may be more completely ascertained if the infection is identified close to onset) vs lower viral load
  3. Up to 0 days before 1^st^ vaccination date or unvaccinated, from 21 days post 1^st^ vaccination date to 13 days post 2^nd^ vaccination date inclusive, from 14 days post 2nd vaccination date onwards. PCR-positives 0-20 days after first vaccination were excluded as symptoms may be due to side-effects.
  4. Age groups 2-5, 6-10, 11-15, 16-44, 45-64, 65+ years
  5. All PCR-positives split between before 1/Sep/2020, 1/Sep/2020-17/Nov/2020, 17/Nov/2020-1/Mar/2021, 1/Mar/2021-17/May/2021, 17/May/2021-17/Jul/2021 on the basis of background incidental symptoms in PCR-negatives (different background rates could be driven either by epidemic dynamics and/or other infections being more prevalent during certain periods)

### Cycle threshold (Ct) values

Each positive test has a Ct value for each positive gene, leading to 1-3 individual Ct values per positive result. As the Spearman correlation between Ct values for each pair of genes (when present together) was very high (>0.98), we first took the arithmetic mean of all Ct values for detected genes for each positive as the single Ct value per positive test. We then took the minimum of these Ct values across all positive tests in an episode as the Ct value for each positive episode.

### Generalised additive models

In regression models for reporting any evidence of symptoms and specific symptoms in those with evidence of symptoms in PCR-positives and PCR-negatives, we truncated age at 85y and Ct at the 5^th^ and 95^th^ percentiles in order to avoid undue influence of outliers. Age was modelled as smoothing spline. Due to small numbers (**Table S3**) we were only able to investigate differences by self-reported ethnicity as white vs non-white.

bam(cbind(n_withsymptom, n_withoutsymptom) ~

s(study_day, bs="bs", k=15, by=Sars_COV_2_positivity) +

s(age_at_visit, bs="bs", k=15, by=Sars_COV_2_positivity) +

sex:Sars_COV_2_positivity + ethnicity_wo:Sars_COV_2_positivity + sex + ethnicity_wo + Sars_COV_2_positivity, family=binomial(link="cloglog"), method = "fREML", data = data, discrete=TRUE, nthreads =12)

bam(cbind(n_withsymptom, n_withoutsymptom) ~

vaccinated:Sars_COV_2_positivity + vaccinated +

s(study_day, bs="bs", k=15, by=Sars_COV_2_positivity) +

s(age_at_visit, bs="bs", k=15, by=Sars_COV_2_positivity) +

sex:Sars_COV_2_positivity + ethnicity_wo:Sars_COV_2_positivity +

sex + ethnicity_wo + Sars_COV_2_positivity, family=binomial(link="cloglog"), method = "fREML", data = data, discrete=TRUE, nthreads =12)

In PCR-positives only:

bam(cbind(n_withsymptom, n_withoutsymptom) ~

s(ct _mean, bs="bs", k=5) +

s(study_day, bs="bs", k=15) +

s(age_at_visit, bs="bs", k=15) +

sex + ethnicity_wo, family=binomial(link="cloglog"), method = "fREML", data = data, discrete=TRUE, nthreads =12)

Also adjusting for S-gene positivity (present/absent/unknown):

bam(cbind(n_withsymptom, n_withoutsymptom) ~

s(ct _mean, bs="bs", k=5) +

S_gene_positivity_pattern +

s(study_day, bs="bs", k=15) +

s(age_at_visit, bs="bs", k=15) +

sex + ethnicity_wo, family=binomial(link="cloglog"), method = "fREML", data = data, discrete=TRUE, nthreads =12)

### Performance metrics

For each combination of symptoms considered, we calculated the number of true positives (≥1 symptom from the combination being considered reported for a PCR-positive; TP), false positives (≥1 symptom reported for a negative visit; FP), true negatives (symptoms in the combination not reported for a negative visit; TN), false negatives (symptoms in the combination not reported in a PCR-positive; FN). We compared sensitivity=TP/(TP+FN); specificity=TN/(TN+FP); positive predictive value=TP/(TP+FP) (PPV); negative predictive value=TN/(TN+FN) (NPV); area under the receiver operating characteristic curve (AUROC), the area under the curve of specificity and 1–sensitivity, which, in this case where we have a binary outcome and a binary exposure, is 0.5*(sensitivity+specificity); tests per case =1/PPV (TPC); and the inflation factor=episodes/visits with ≥1 symptom/episodes/visits with classic symptoms.

### Logistic regression

To assess the association of each symptom with infection in symptomatic PCR-positives or PCR-negatives, we used logistic regression with SARS-CoV-2 positivity as the outcome and the 12 elicited symptoms as the explanatory variables, univariably, mutually adjusted for all symptoms, and mutually adjusted for all symptoms, age, sex and ethnicity (as for the GAM models).

# Results

## Defining the negative visit comparator group

**Table S1** shows the total number of test-negative visits and the number of participants in which they occur, in all PCR-negative visits and restricting to those visits with evidence of symptoms only, for each step of the hierarchical restriction of test-negative visits to form the comparator. This was done in order to exclude potential contamination from undetected positives, ongoing symptoms after detected positives, and symptoms due to vaccination. Overall, 75% of test-negative visits in 93% participants with any test-negative visit were retained in analyses, and 62% of test-negative visits where any symptoms were reported in 68% of participants with any test-negative visit where any symptoms were reported. Most test-negative visits were excluded because of self-isolation or contact with definite positives. The larger exclusion of test-negative visits where any symptoms were reported, despite the fact that symptoms were not a reason for exclusion (with the exception of visits from a small number of individuals reporting loss of taste/smell in 2020 before tests were available) illustrate the likely contamination of all test-negative visits with both undetected positives and ongoing symptoms post COVID-19 infection (**Table S2**).

Symptom co-reporting in symptomatic PCR-positives was broadly similar regardless of Ct, variant, vaccination status or age (**Fig.S2**), although myalgia was less strongly co-reported with headache/fatigue in those 6-15y, with Delta and in those ≥14d post-second vaccination.

Comparing the probability of reporting specific symptoms in PCR-negatives with evidence of symptoms over time before (**Fig.S2**) and after (**Fig.2**) the final exclusion of negative visits that occurred within [-7,+14 days] of either first or second vaccination date, the substantially higher probabilities of reporting fever, headache, fatigue weakness, muscle ache myalgia and nausea-vomiting in the first quarter of 2021 are plausibly driven by side effects of vaccination, as these probabilities markedly reduce after the exclusion.

All symptoms were associated with SARS-CoV-2 positivity univariably (**Fig.S10**); as expected, associations attenuated in mutually adjusted models. Associations were strongest for reporting loss of smell or taste, and then fever, fatigue weakness, cough, muscle ache myalgia, headache and shortness of breath, with lower odds associated with reporting sore throat, diarrhoea, nausea vomiting or abdominal pain adjusting for other symptoms.

Among all symptomatic PCR-positives, the percentage meeting the current UK criteria (loss of taste or loss of smell or fever or cough) was 74% (TPC 4.6), while the percentage meeting the US definition (any of the 12 elicited symptoms) was 90% (TPC 8.7), with 2.3 fold higher number of tests with the US definition compared to the UK definition (defined as inflation) (**Table S7**). Considering individual age groups, these figures were, respectively, 68% (TPC 19.5) and 83% (TPC 19.6), inflation 1.2 for 2-5 years; 60% (TPC 9.5) and 82% (TPC 12.7), inflation 1.8 for 6-10 years; 68% (TPC 4.2) and 89% (TPC 6.8), inflation 2.1 for 11-15 years; 76% (TPC 3.7) and 90% (TPC 7.2), inflation 2.3 for 16-44 years; 76% (TPC 3.9) and 91% (TPC 8.2), inflation 2.5 for 45-64 years; 69% (TPC 7.1) and 88% (TPC 14.5) inflation 2.6 for 65+ years. Sensitivity, specificity, AUROC, TPC and inflation are provided for these, as well as for all the best performing combinations in terms of sensitivity, AUROC, TPC and inflation overall and for subgroups by Ct, variant, vaccination status and age in **Table S7**.

Figure S1. Percentage of positive episodes with each S-gene positivity pattern over time


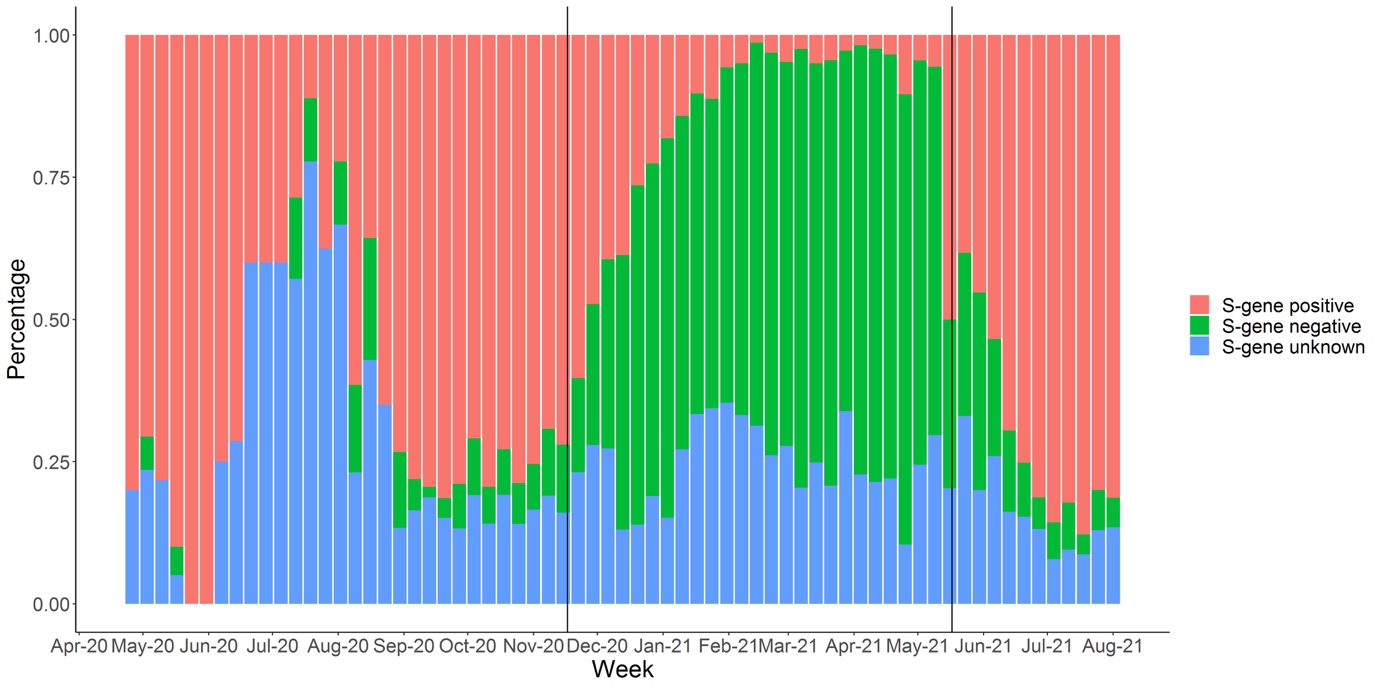


Note: vertical lines at 17 November 2020 and 17 May 2021. S-gene positive if the S-gene was ever detected within any positive test in the episode (by definition, with N/ORF1ab/both) (wild-type/Delta-compatible), otherwise S-gene negative if positive at least once for ORF1ab+N (Alpha-compatible), otherwise S-gene unknown (N-only/ORF1ab-only).

Figure S2. Probability of reporting specific symptoms at negative-visits with any evidence of symptoms before the final exclusion of negative visits where visit date is within [-7,+14] days of date of either first or second vaccination


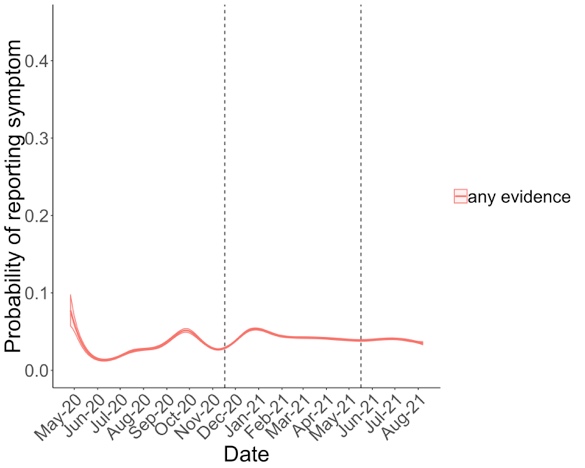

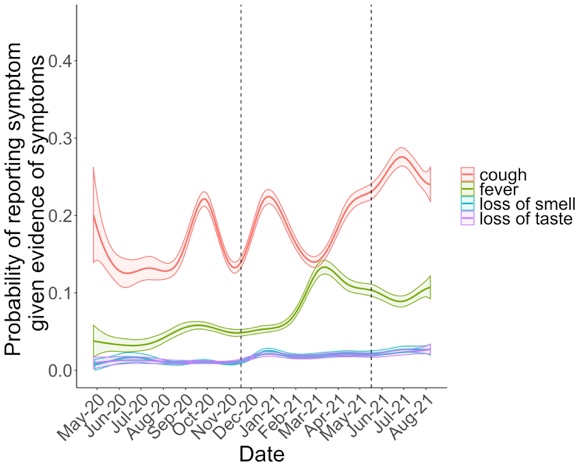

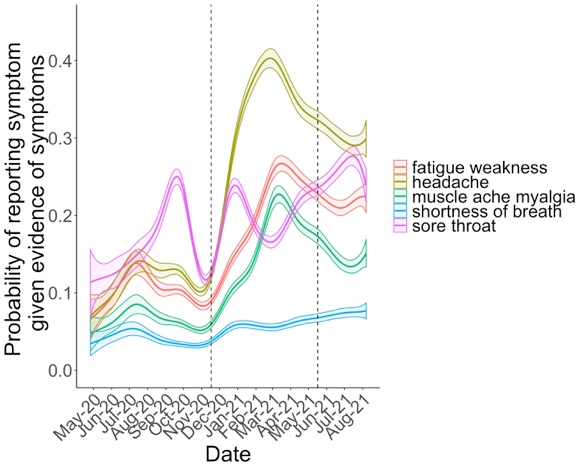

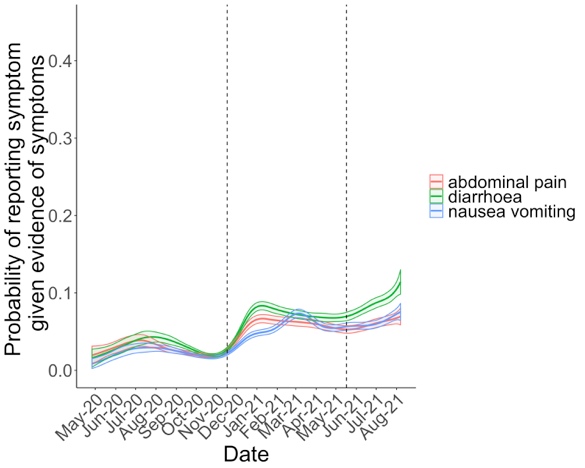


Note: adjusted for sex, age and ethnicity (reference category age 45, male, white ethnicity). Vaccination programme started 8 December 2020, but increased in magnitude in January 2021.

Figure S3. Hierarchical clustering of symptoms

1. **Test-negative visits (left) and all positive episodes (right)**

|  |  |
| --- | --- |

1. **Ct<30 (left) and Ct≥30 (right)**

| **** | **** |
| --- | --- |

1. **Variant: wild-type compatible, Ct<30 (left), Alpha-compatible, Ct<30 (middle) and Delta-compatible, Ct<30 (right)**

| **** | **** | **** |
| --- | --- | --- |

1. **Not yet vaccinated (left), 21 days after first vaccination and before second vaccination (middle) and 14 days or more after second vaccination (right)**

| **** | **** | **** |
| --- | --- | --- |

1. **Age**

**2-5y 6-10y 11-15y**

| **** | **** | **** |
| --- | --- | --- |

**16-44 y 45-64y 65+y**

| **** | **** | **** |
| --- | --- | --- |

Note: different scales.

Figure S4. Probability of reporting any evidence of symptoms, as well as the probability of reporting each of the 12 symptoms in those with evidence of symptoms by mean Ct (model adjusted for age, sex and ethnicity and S-gene positivity pattern).


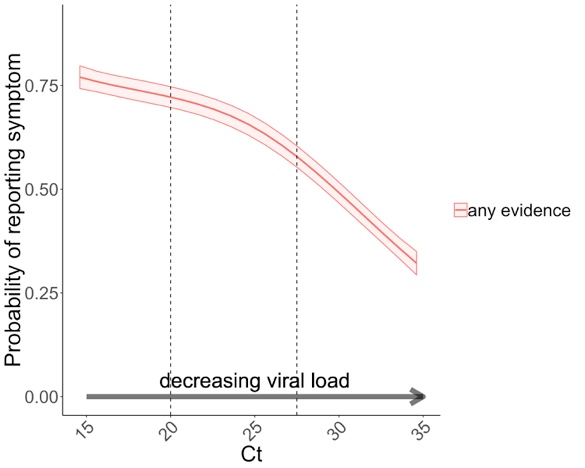

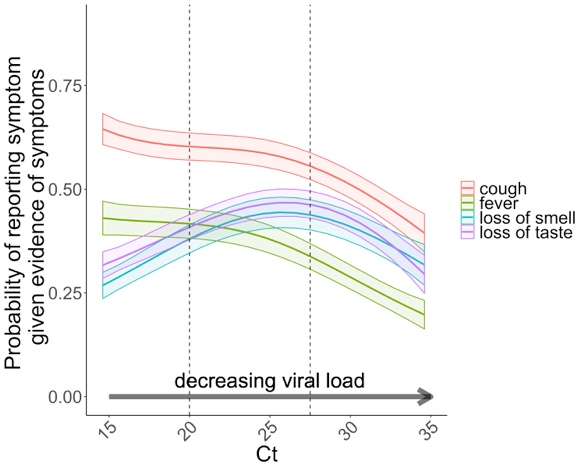

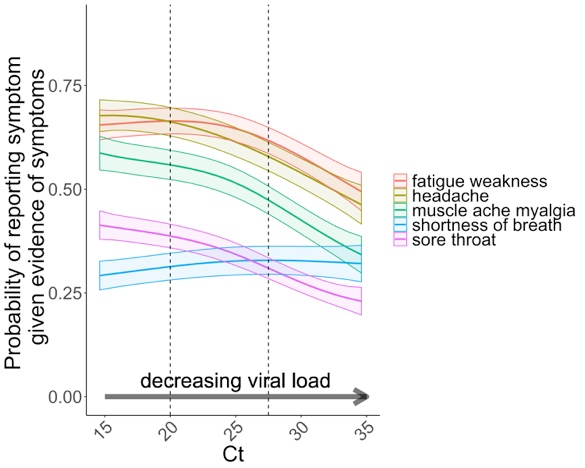

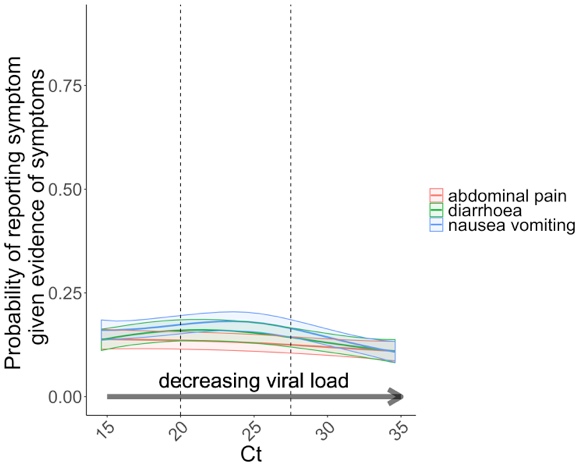


Figure S5. By Ct, performance of individual symptoms, as well as the classic four symptoms (cough, fever, loss of taste/smell), classic plus all possible combinations of 1/2/3/4 symptoms, and any of the 12 named symptoms, in predicting SARS-CoV-2 positivity in those with evidence of symptoms in terms of sensitivity and overall accuracy (AUROC).

A. Ct<30 B. Ct≥30


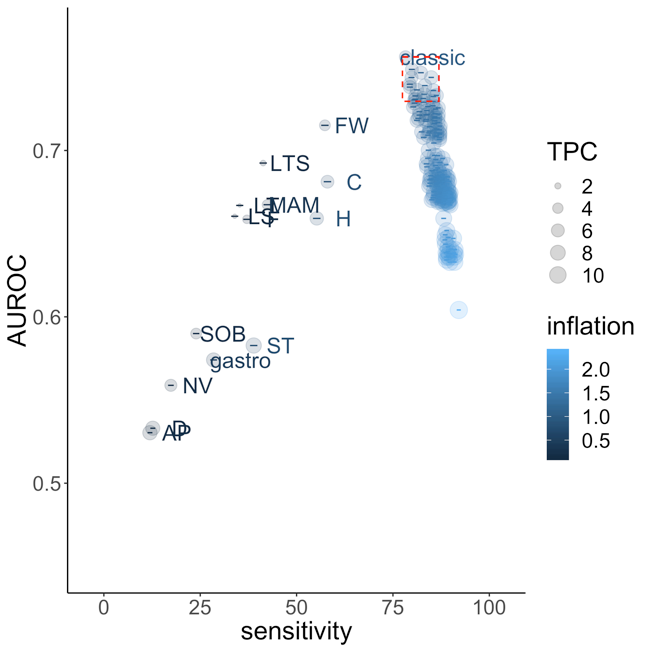

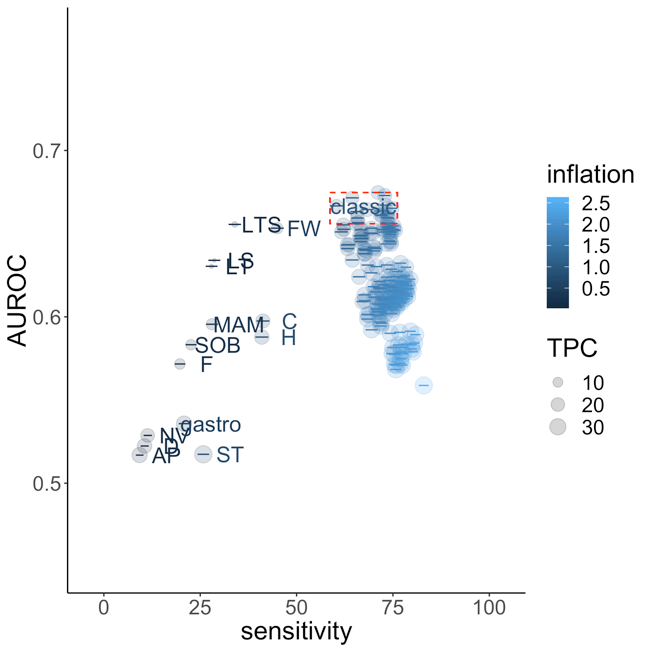


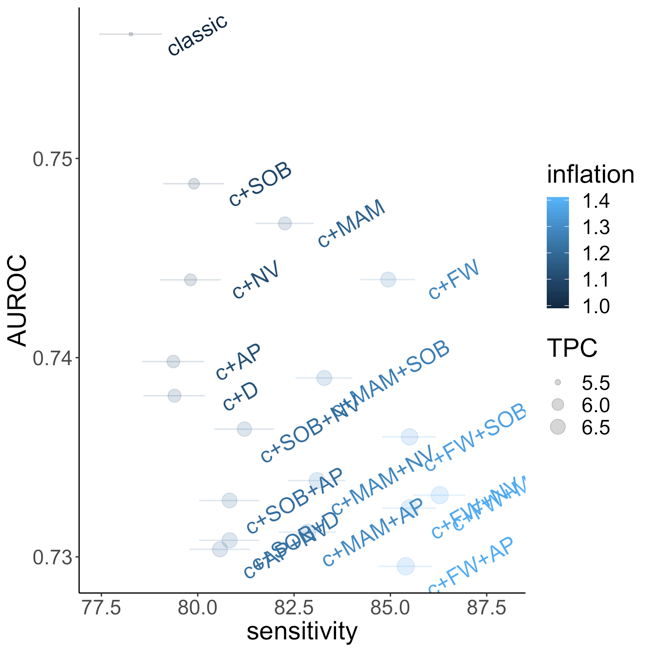

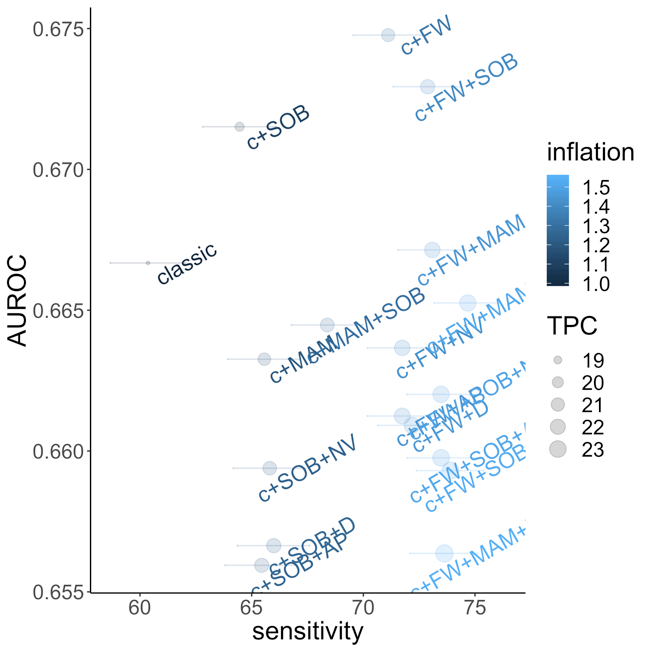


Note: For exact values see Table S8. Bottom row is an expanded version of the top right corner of the top row panels (red box, AUROC >90^th^ quantile, sensitivity > sensitivity of combination of classic 4 symptoms). Inflation (relative numbers reporting these symptoms compared to classic symptoms) and tests per positive case (TPC) are also included in the visualisation. TPC=1/positive predictive value. By definition, as the number of symptoms increases, sensitivity also increases.

Note: abbreviations: c – classic, Fever - F, Headache - H, Muscle ache/myalgia - MAM, Weakness/tiredness - FW, Nausea/vomiting - NV, Abdominal pain - AP, Diarrhoea - D, Sore throat - ST, Cough - C, Shortness of breath - SOB, Loss of taste - LT, Loss of smell – LS, Loss of taste or smell – LTS

Figure S6. By viral variant, performance of individual symptoms, as well as the classic four symptoms (cough, fever, loss of taste/smell), classic plus all possible combinations of 1/2/3/4 symptoms, and any of the 12 named symptoms, in predicting SARS-CoV-2 positivity in those with evidence of symptoms in terms of sensitivity and overall accuracy (AUROC).

A. Wild type, Ct<30 B. Alpha-compatible, Ct<30 C. Delta-compatible, Ct<30


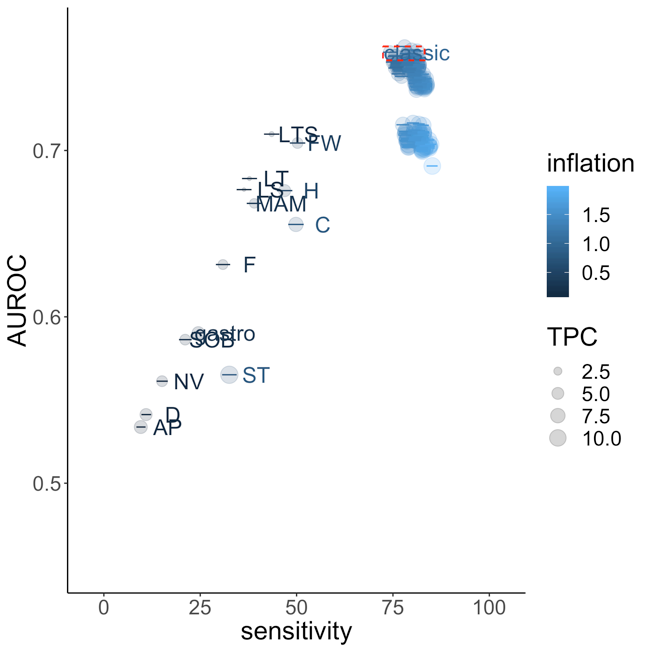

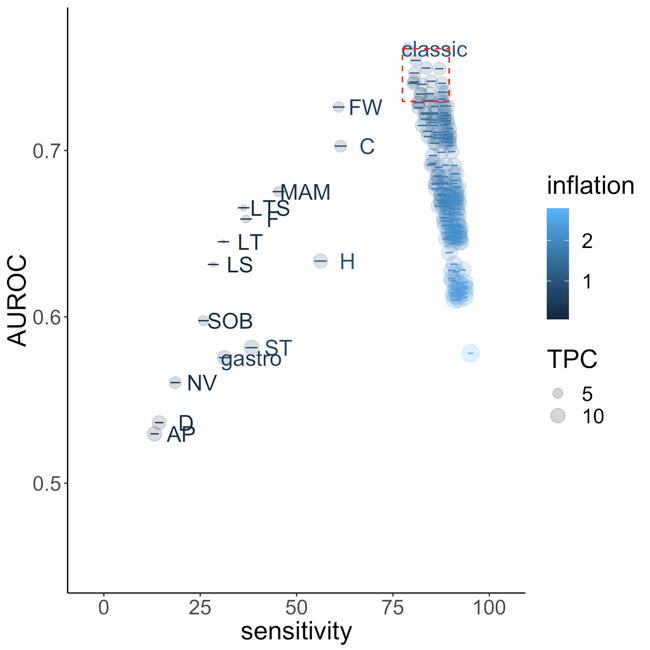

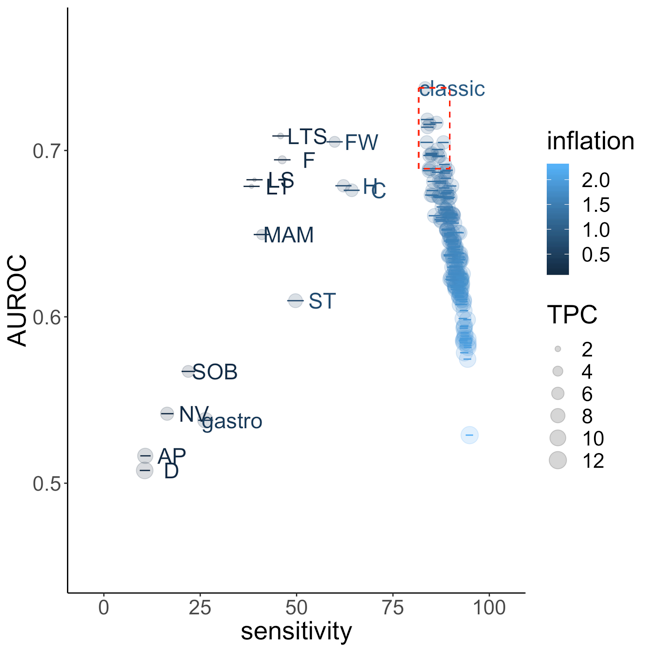


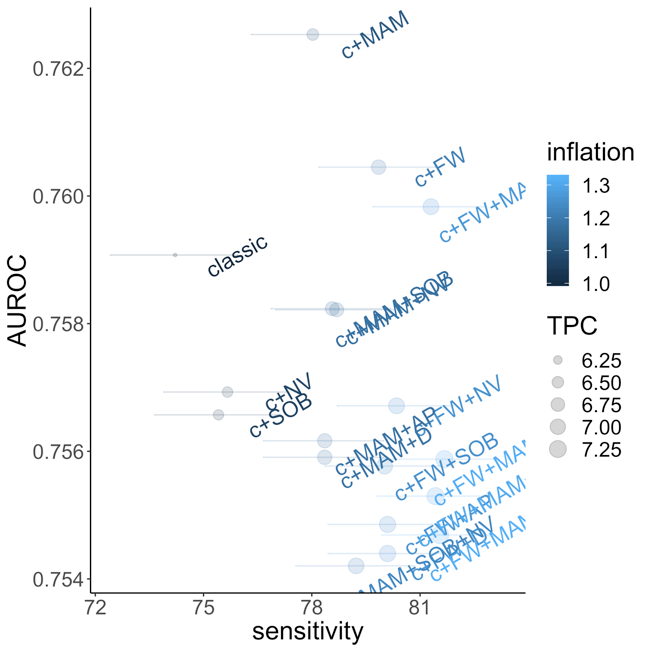

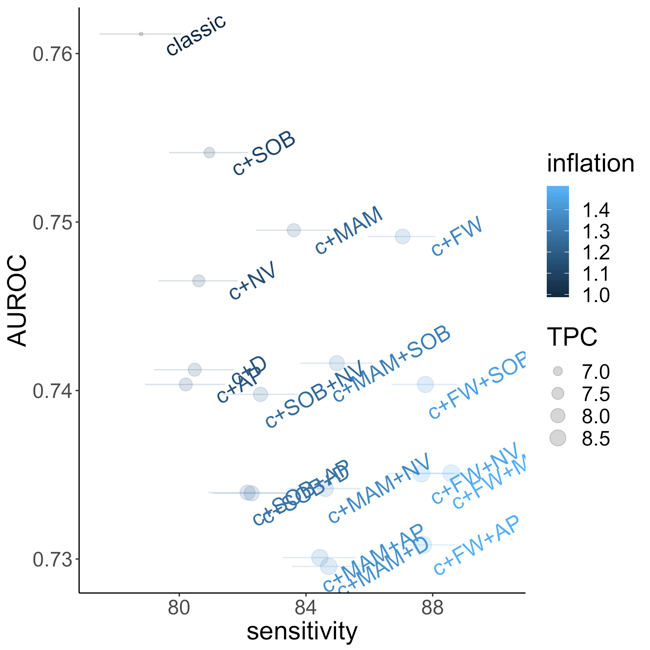

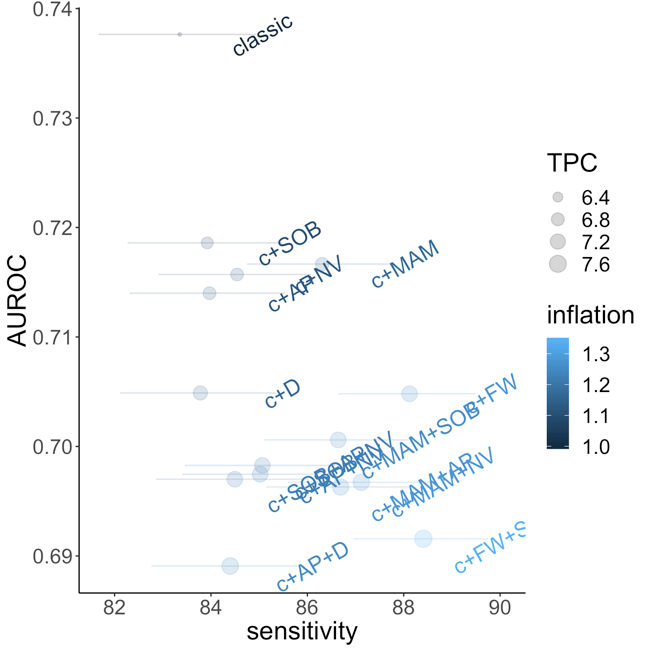


Note: For exact values see Table S8. Bottom row is an expanded version of the top right corner of the top row panels (red box, AUROC >90^th^ quantile, sensitivity > sensitivity of combination of classic 4 symptoms). Inflation (relative numbers reporting these symptoms compared to classic symptoms) and tests per positive case (TPC) are also included in the visualisation. TPC=1/positive predictive value. By definition, as the number of symptoms increases, sensitivity also increases.

Note: abbreviations: c – classic, Fever - F, Headache - H, Muscle ache/myalgia - MAM, Weakness/tiredness - FW, Nausea/vomiting - NV, Abdominal pain - AP, Diarrhoea - D, Sore throat - ST, Cough - C, Shortness of breath - SOB, Loss of taste - LT, Loss of smell – LS, Loss of taste or smell – LTS

Figure S7. By vaccination status, performance of individual symptoms, as well as the classic four symptoms (cough, fever, loss of taste/smell), classic plus all possible combinations of 1/2/3/4 symptoms, and any of the 12 named symptoms, in predicting SARS-CoV-2 positivity in those with evidence of symptoms in terms of sensitivity and overall accuracy (AUROC).

A. not yet vaccinated B. ≥21 days post 1^st^ vaccination C. ≥14 days post 2^nd^ vaccination and before 2^nd^ vaccination


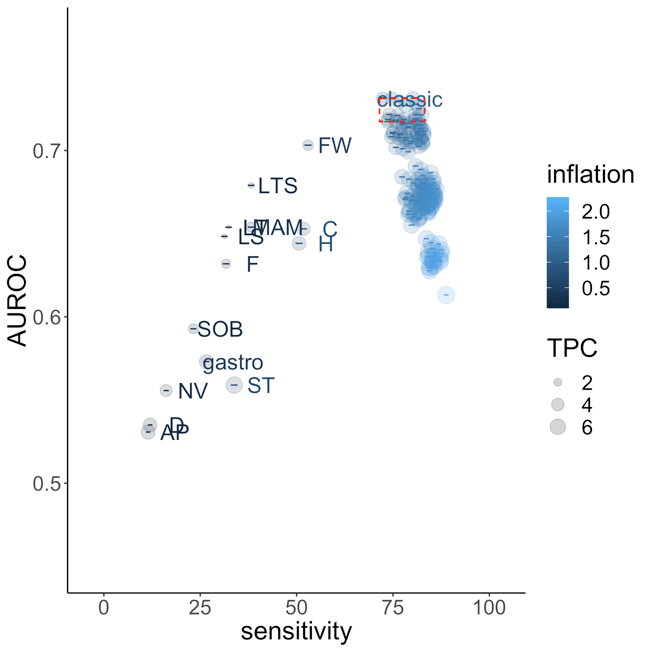

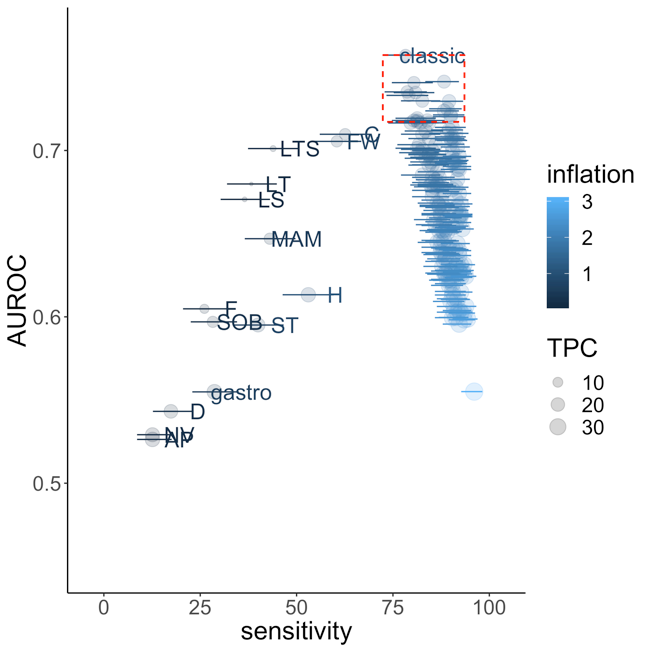

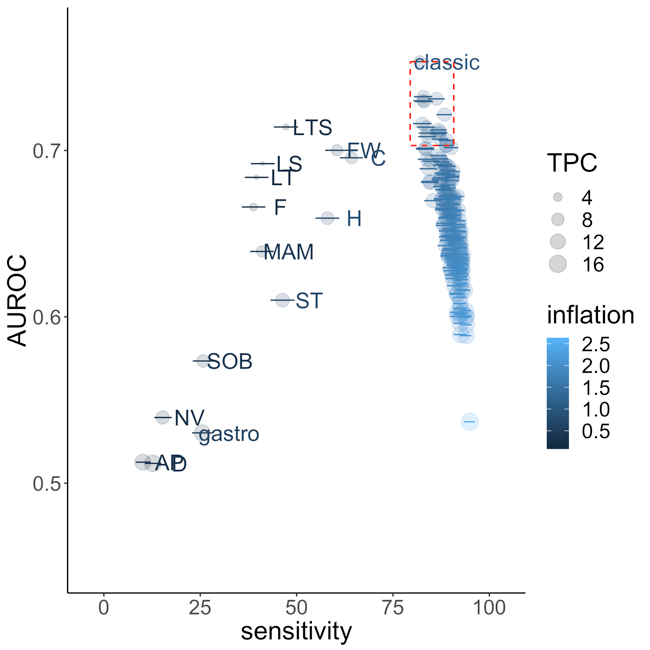


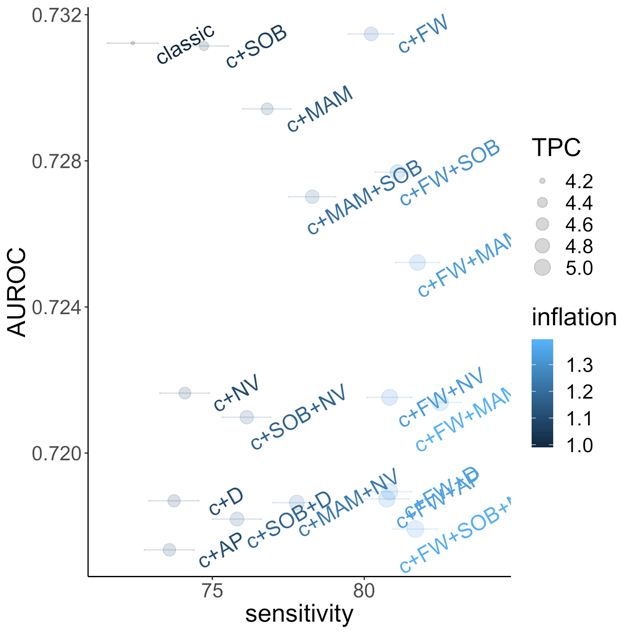

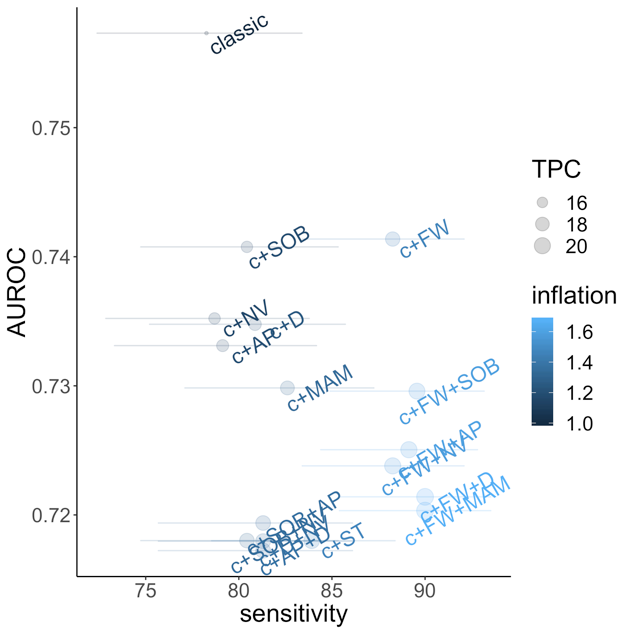

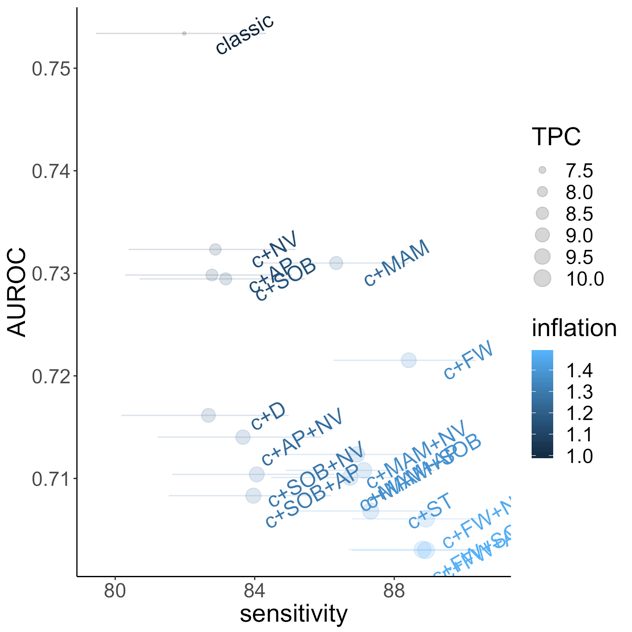


Note: For exact values see Table S8. Bottom row is an expanded version of the top right corner of the top row panels (red box, AUROC >90^th^ quantile, sensitivity > sensitivity of combination of classic 4 symptoms). Inflation (relative numbers reporting these symptoms compared to classic symptoms) and tests per positive case (TPC) are also included in the visualisation. TPC=1/positive predictive value. By definition, as the number of symptoms increases, sensitivity also increases.

Note: abbreviations: c – classic, Fever - F, Headache - H, Muscle ache/myalgia - MAM, Weakness/tiredness - FW, Nausea/vomiting - NV, Abdominal pain - AP, Diarrhoea - D, Sore throat - ST, Cough - C, Shortness of breath - SOB, Loss of taste - LT, Loss of smell – LS, Loss of taste or smell – LTS

Figure S8. By age, performance of individual symptoms, as well as the classic four symptoms (cough, fever, loss of taste/smell), classic plus all possible combinations of 1/2/3/4 symptoms, and any of the 12 named symptoms, in predicting SARS-CoV-2 positivity in those with evidence of symptoms in terms of sensitivity and overall accuracy (AUROC).

A. 2-5 B. 6-10 C. 11-15


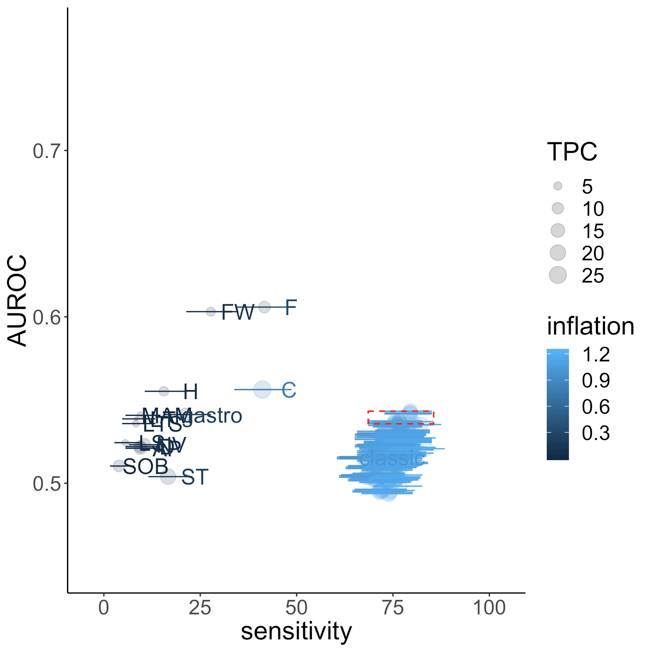

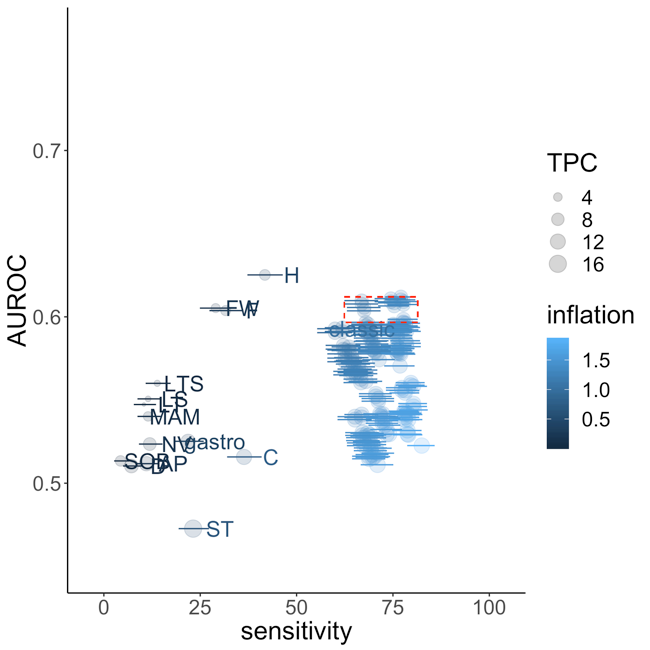

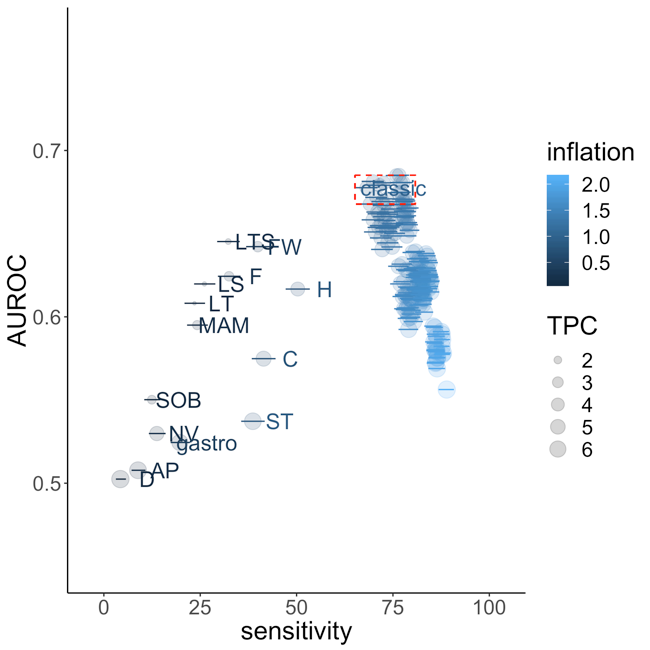


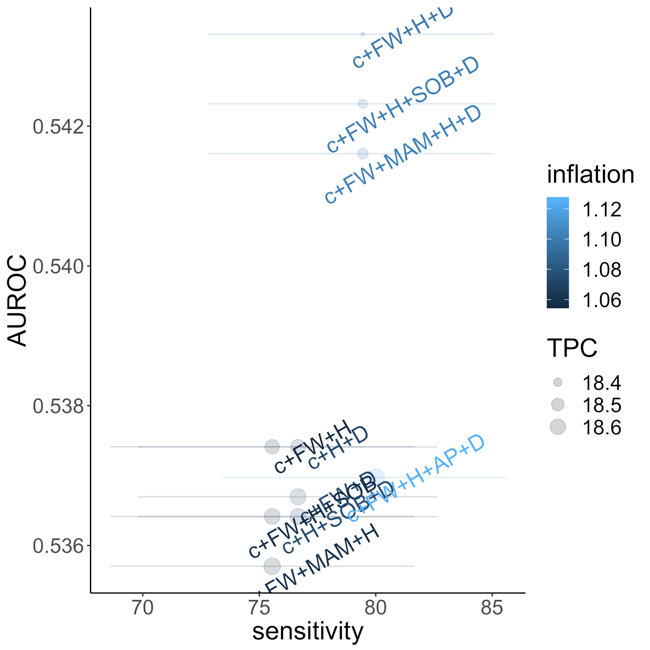

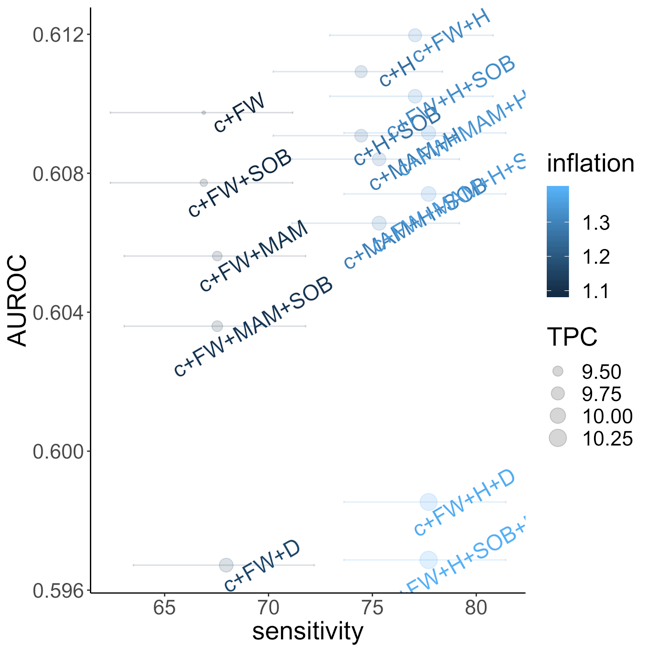

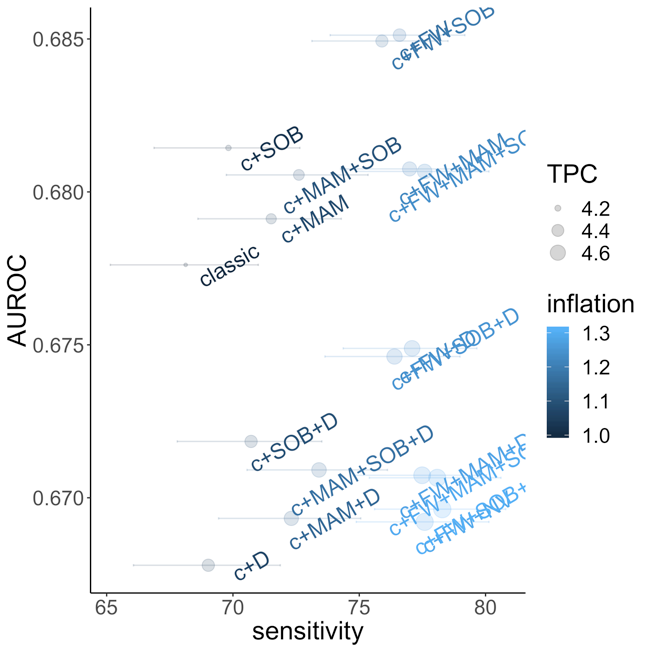


D. 16-44 E. 45-64 F. 65+


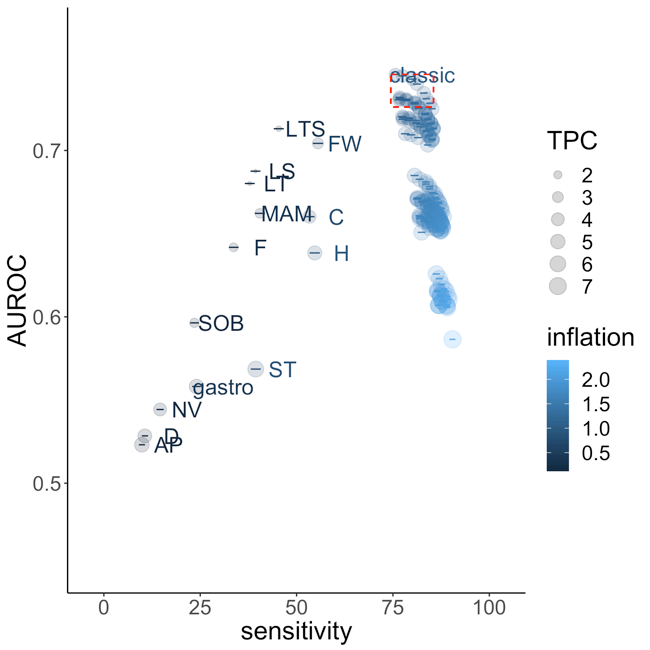

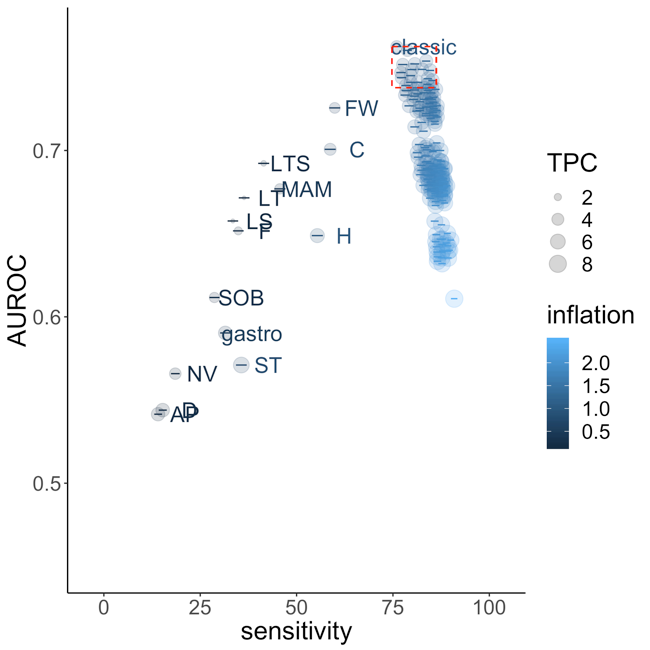

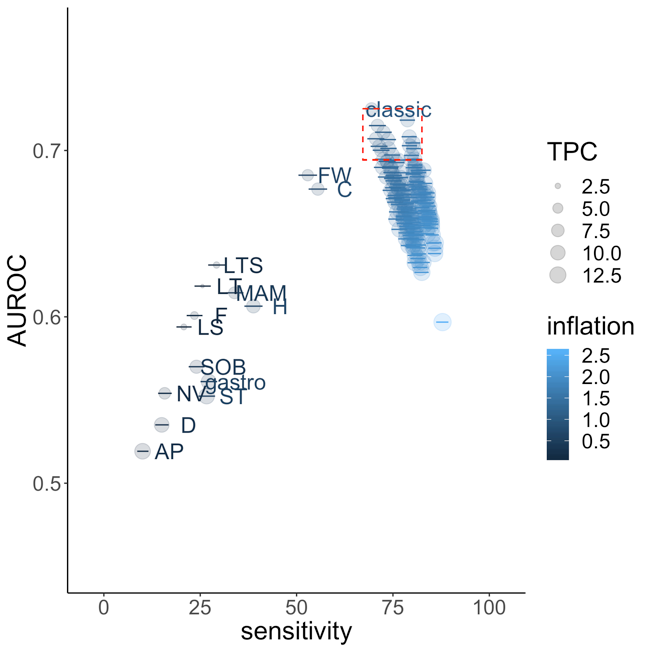


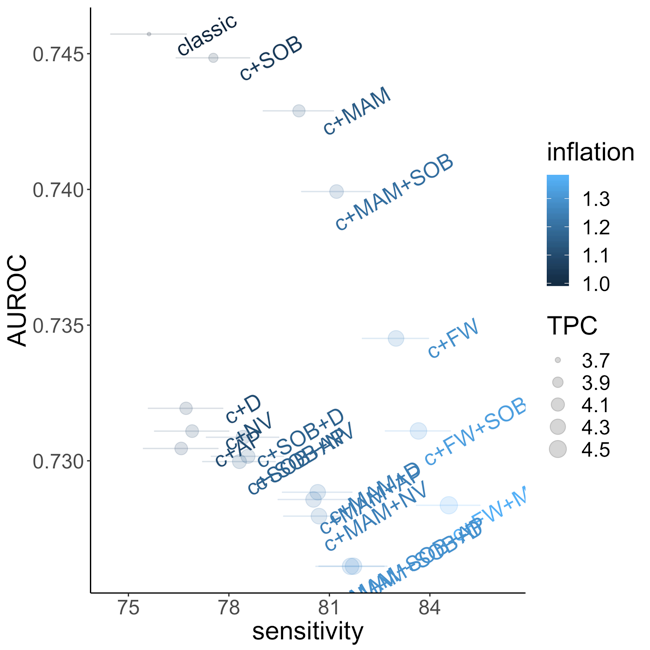

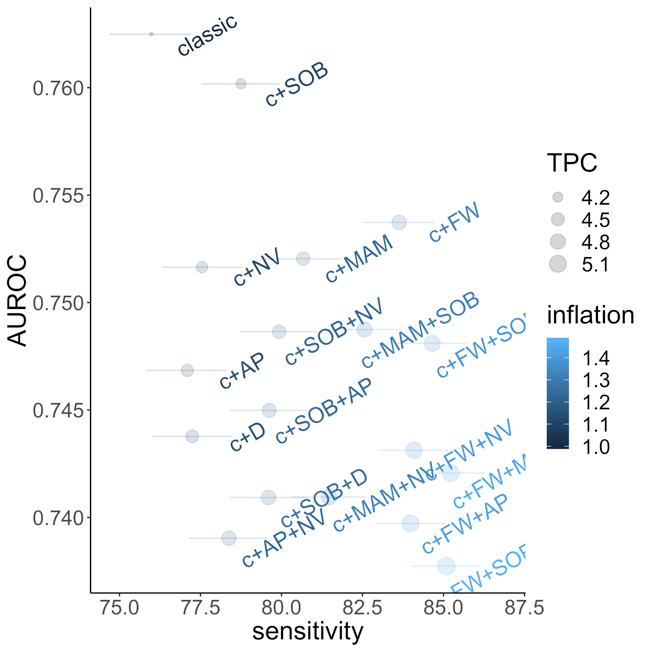

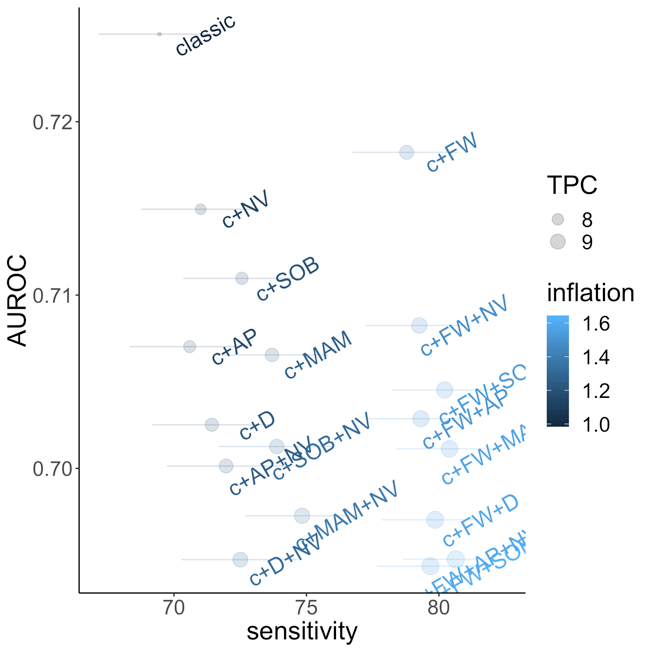


Note: For exact values see Table S8. Bottom row is an expanded version of the top right corner of the top row panels (red box, AUROC >90^th^ quantile, sensitivity > sensitivity of combination of classic 4 symptoms). Inflation (relative numbers reporting these symptoms compared to classic symptoms) and tests per positive case (TPC) are also included in the visualisation. TPC=1/positive predictive value. By definition, as the number of symptoms increases, sensitivity also increases.

Note: abbreviations: c – classic, Fever - F, Headache - H, Muscle ache/myalgia - MAM, Weakness/tiredness - FW, Nausea/vomiting - NV, Abdominal pain - AP, Diarrhoea - D, Sore throat - ST, Cough - C, Shortness of breath - SOB, Loss of taste - LT, Loss of smell – LS, Loss of taste or smell – LTS

Figure S9. By time period, performance of individual symptoms, as well as the classic four symptoms (cough, fever, loss of taste/smell), classic plus all possible combinations of 1/2/3/4 symptoms, and any of the 12 named symptoms, in predicting SARS-CoV-2 positivity in those with evidence of symptoms in terms of sensitivity and overall accuracy (AUROC).

A. 26/Apr/2020-1/Sep/2020 B. 1/Sep/2020-17/Nov/2020 C.17/Nov/2020-1/Mar/2021


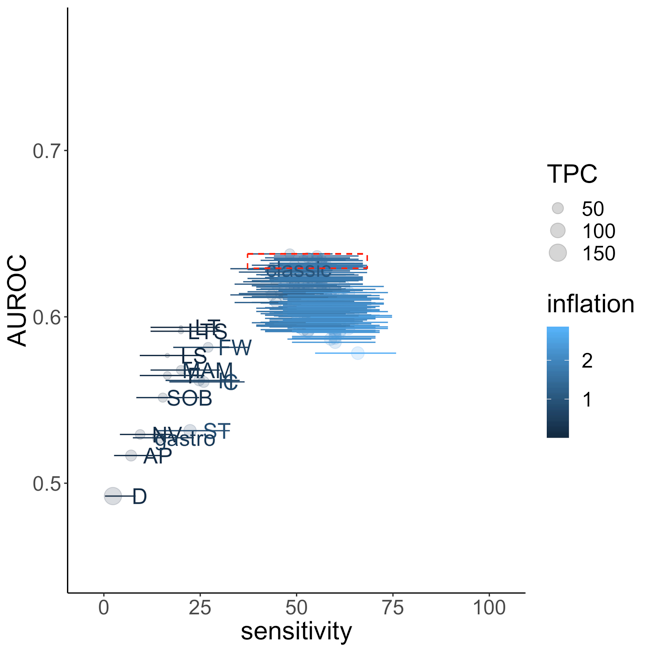

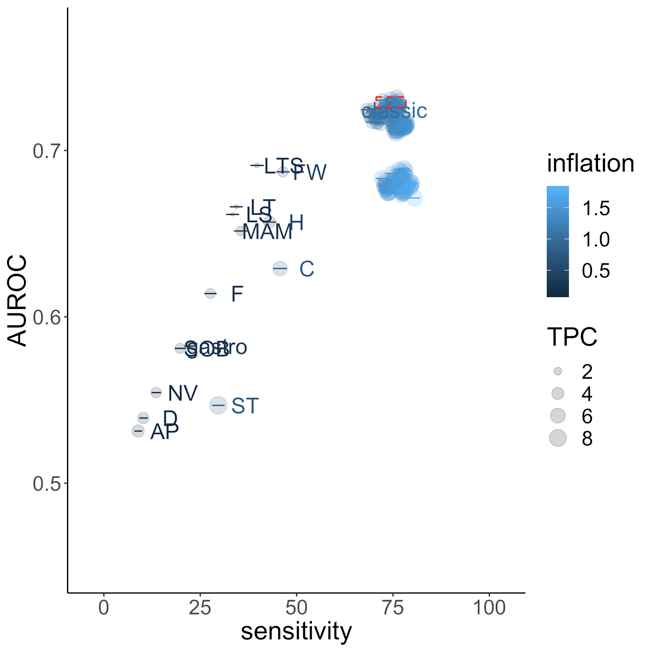

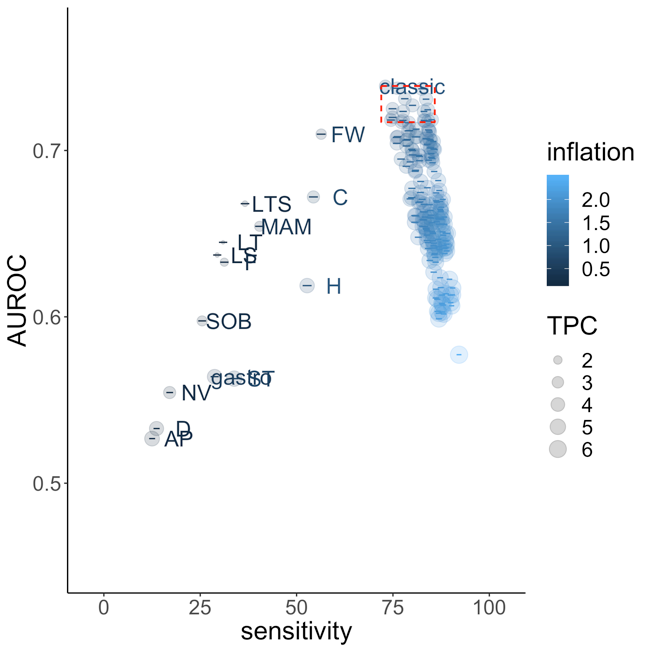


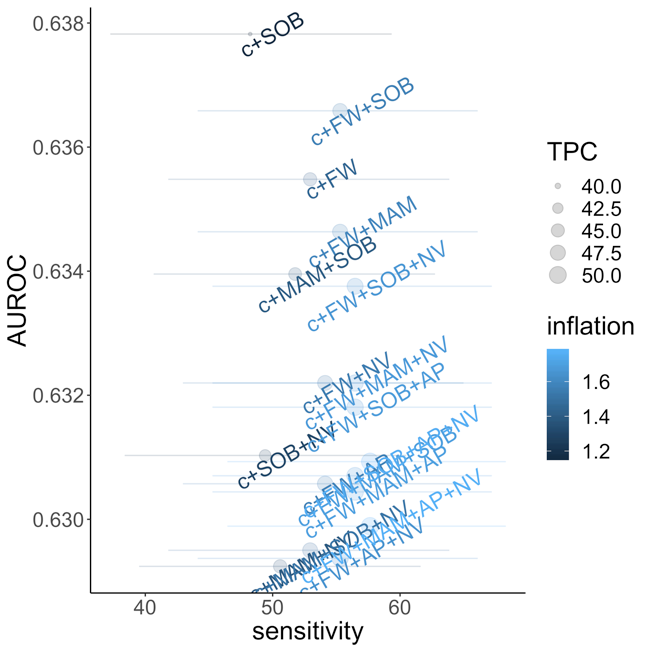

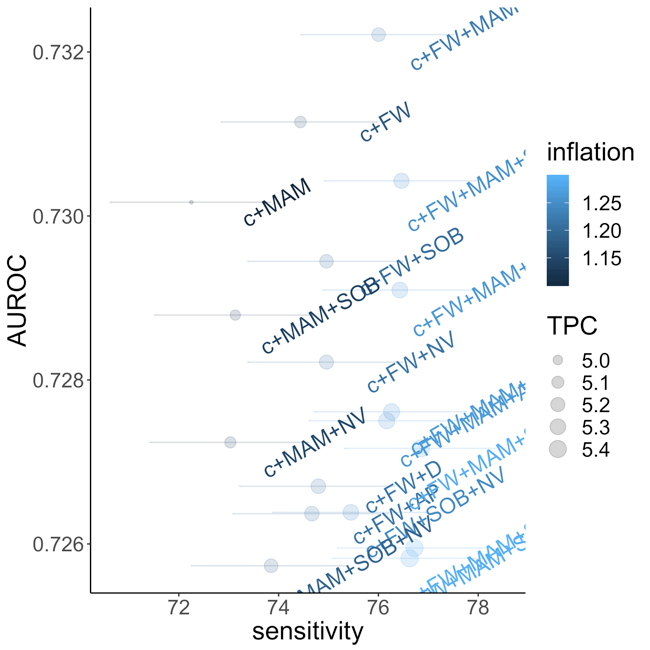

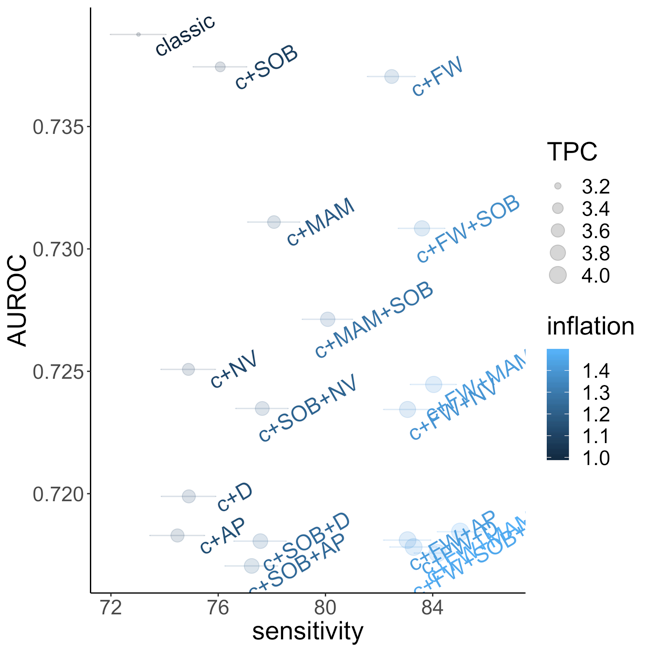


D. 1/Mar/2021-17/May/2021 E. 17/May/2021-7/Aug/2021


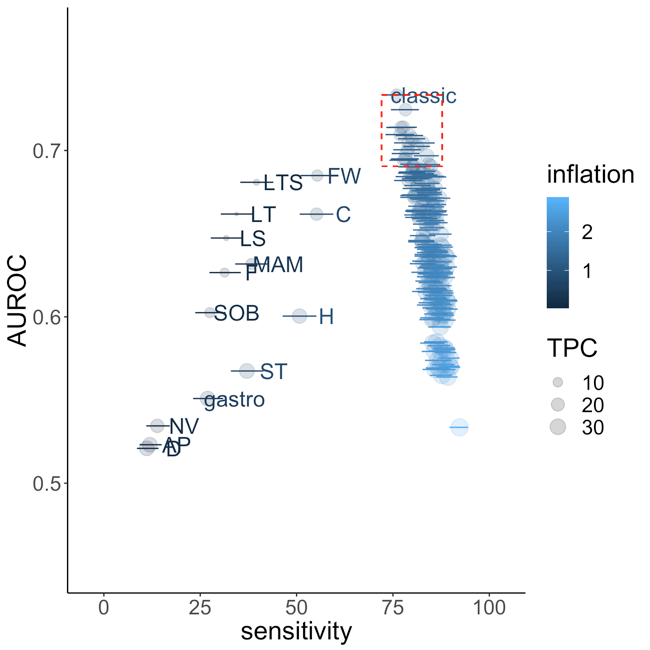

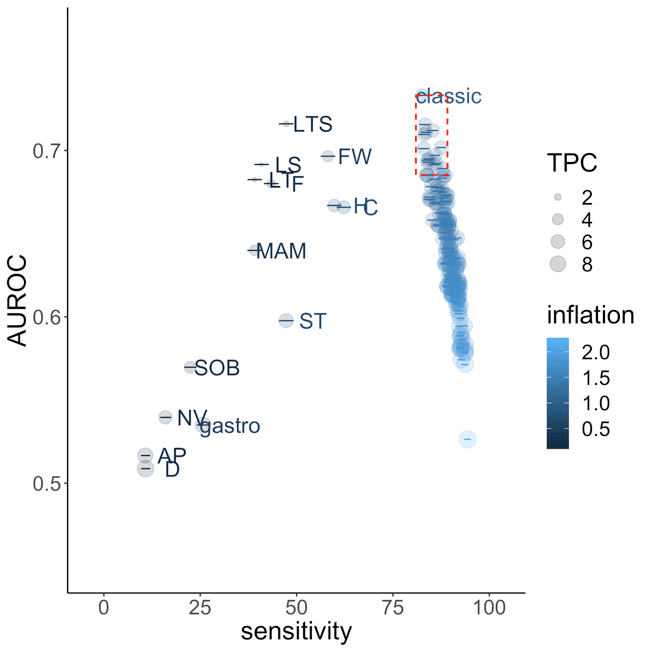


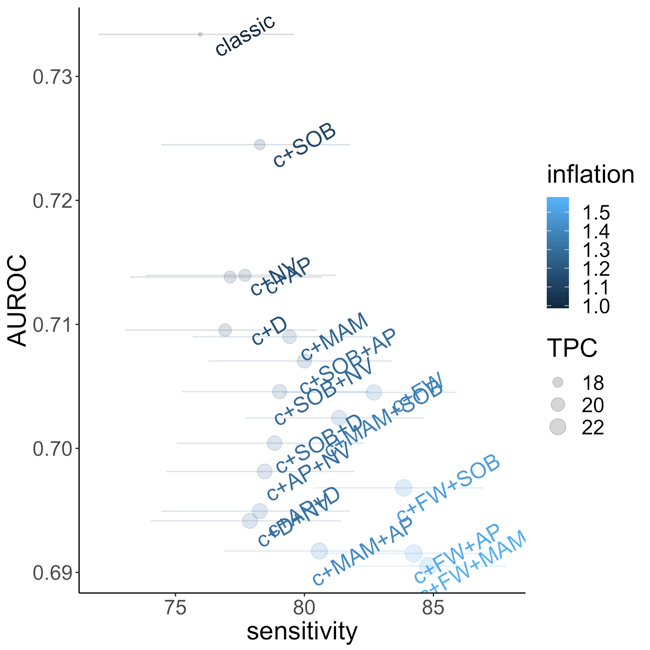

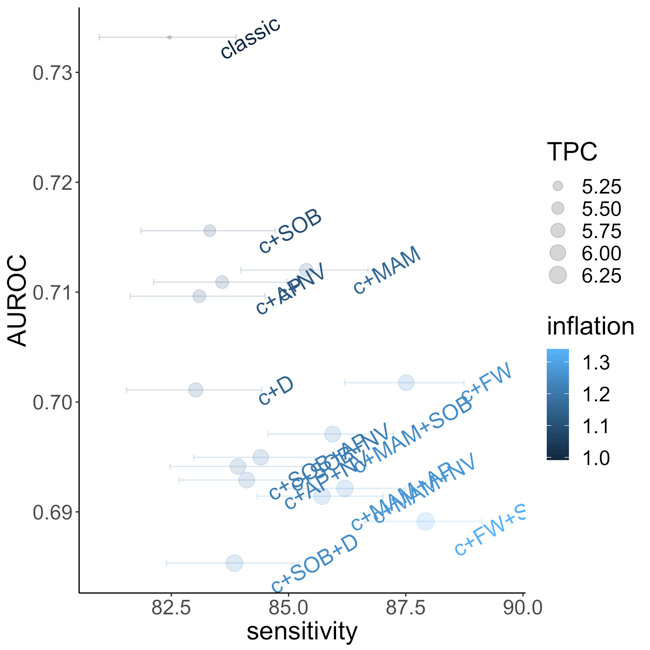


Note: For exact values see Table S8. Bottom row is an expanded version of the top right corner of the top row panels (red box, AUROC >90^th^ quantile, sensitivity > sensitivity of combination of classic 4 symptoms). Inflation (relative numbers reporting these symptoms compared to classic symptoms) and tests per positive case (TPC) are also included in the visualisation. TPC=1/positive predictive value. By definition, as the number of symptoms increases, sensitivity also increases.

Note: abbreviations: c – classic, Fever - F, Headache - H, Muscle ache/myalgia - MAM, Weakness/tiredness - FW, Nausea/vomiting - NV, Abdominal pain - AP, Diarrhoea - D, Sore throat - ST, Cough - C, Shortness of breath - SOB, Loss of taste - LT, Loss of smell – LS, Loss of taste or smell – LTS

Figure S10. SARS-CoV-2 positivity odds ratios from a logistic regression model with 12 elicited symptoms as the explanatory variables, univariably, mutually adjusted for all symptoms, and mutually adjusted for all symptoms, age, sex and ethnicity


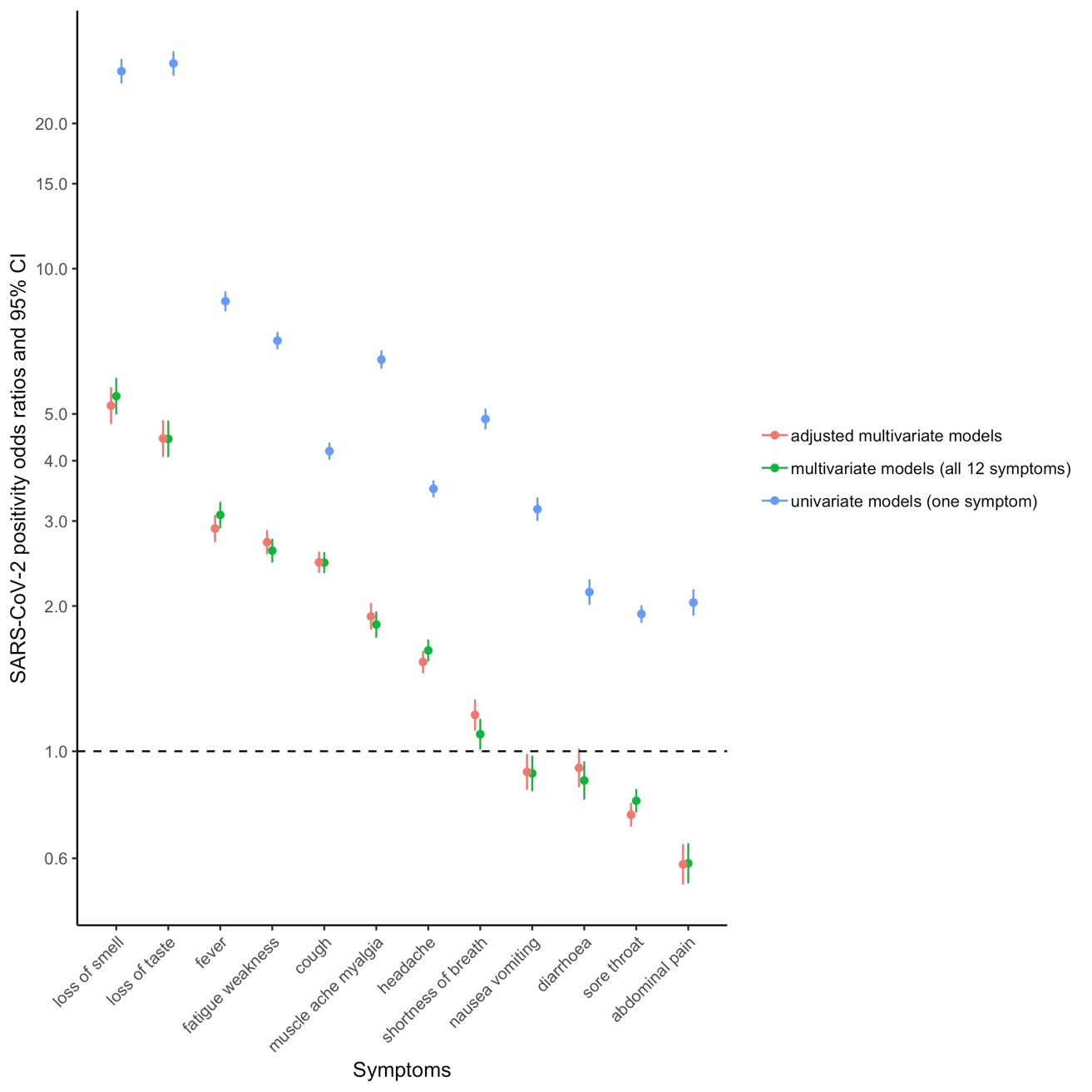


Figure S11. Distribution of Ct for each of the 12 symptoms in positive episodes with symptoms absent at all visits within 35 days of the index positive (00), absent at the index positive, but present at least one subsequent visit (01), present at the index positive, but absent at subsequent visits (10), present initially and at least one subsequent visit (11)


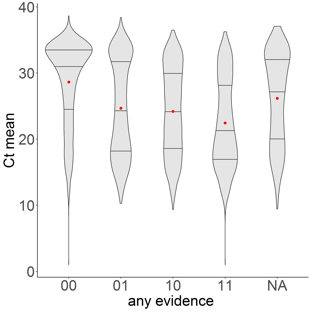


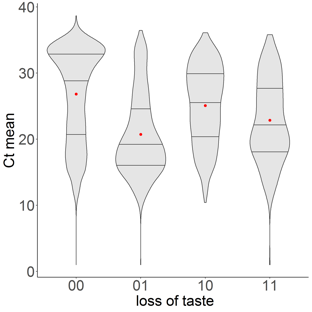

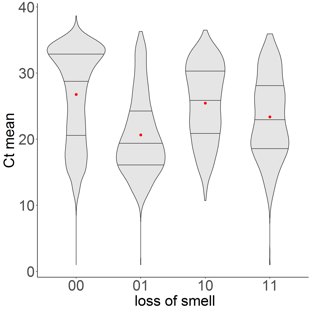

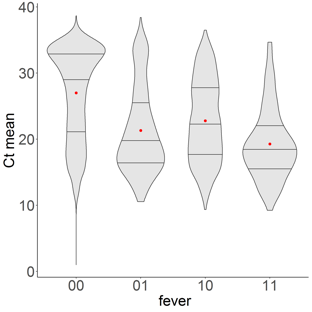

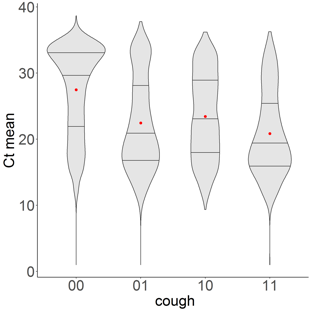


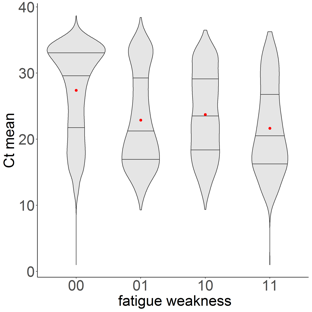

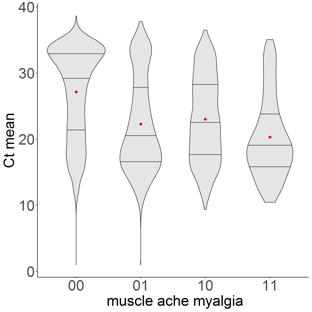

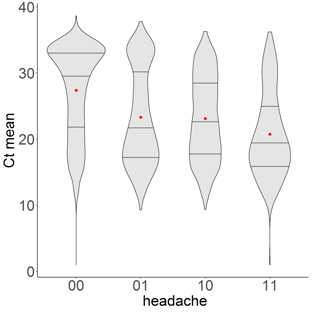

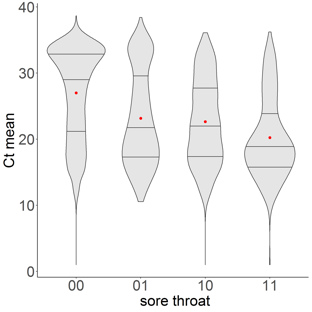

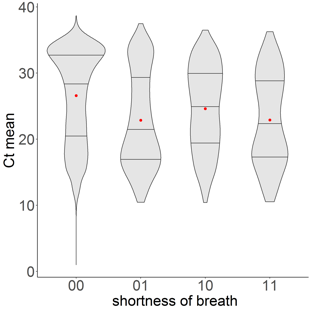

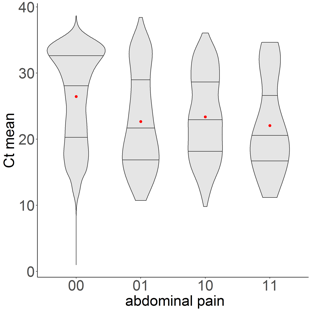

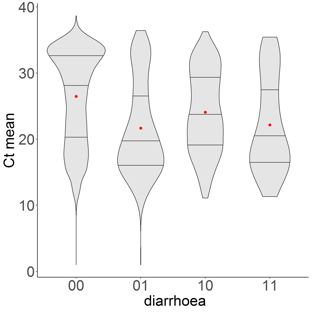

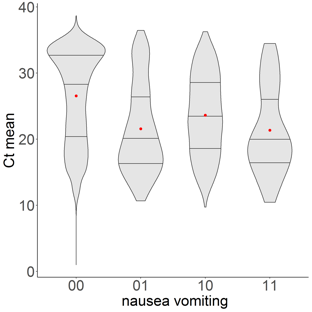


Table S1 Negative visits; summary of exclusions

| **Negative**  **visits**  **excluding** | **With and without reported symptoms** | | **With reported symptoms** | |
| --- | --- | --- | --- | --- |
|  | **Visits**  **(% from first row)** | **Participants**  **(% from first row)** | **Visits**  **(% from first row)** | **Participants**  **(% from first row)** |
| **0** | 5,095,824 | 489,804 | 209,281 | 142,513 |
| **1** | 4,981,365 (98%) | 482,963 (99%) | 202,429 (97%) | 138,393 (97%) |
| **1+2** | 4,795,502 (94%) | 471,641 (96%) | 189,041 (90%) | 130,242 (91%) |
| **1+2+3** | 4,708,088 (92%) | 464,232 (95%) | 183,380 (88%) | 126,777 (89%) |
| **1+2+3+4** | 4,702,276 (92%) | 463,638 (95%) | 182,128 (87%) | 126,183 (89%) |
| **1+2+3+4+5** | 4,172,632 (82%) | 458,510 (94%) | 149,822 (72%) | 108,773 (76%) |
| **1+2+3+4+5+6** | 3,806,692 (75%) | 457,215 (93%) | 130,612 (62%) | 97,186 (68%) |
| **Summary** | **130,612/3,806,692** **(3.4%) of negative visits reported symptoms**  **97,816/457,215 (21%) of participants in the negative cohort reported symptoms** | | | |

1. Visits where PCR test negative
2. **From -90 days before** the first antibody positive test in the study prior to **vaccination**, where antibody results are likely to represent previous undetected infection;
3. **From -35 days before** the first positive onwards from individuals who ever tested PCR positive in the study or in the linked English testing programme (to avoid ongoing long COVID symptoms,[1] and COVID-19-related symptoms shortly before the positive test);
4. **From -35 days before** any self-reported positive swab test result onwards (for the same reason; reflecting the fact that individuals may have obtained tests elsewhere)
5. From a small number of individuals who reported either loss of taste or loss of smell at their first study visit and had no national testing programme result within [-21,+21] days (all before 1 July 2020), given the high specificity of this symptom for COVID-19 infection, the fact that it would have been impossible for these individuals to get an external test and the potential for subsequent symptoms to represent long COVID
6. Where participants reported self-isolating OR contact with **definite** positives in the preceding 28 days (since these individuals have much higher risk of SARS-CoV-2 infection which may not have been detected) and the **previous and the next visit** (because of higher risk of unidentified positivity, and because they may have been contact traced through the national training programme they may be more likely to report symptoms through recall bias, regardless of status)
7. Occurring within [-7,+14 days] of either first or second vaccination date[2], to avoid the inclusion of common symptoms caused by vaccination in the test-negative comparator group and to reflect the possibility of small inaccuracies in reported date of vaccination for some participants

Table S2 Numbers with each symptom in test-negative visits and percentage remaining from each as exclusion becomes stricter

|  | **Visits where PCR test negative** | **Additionally, exclude all visits from -90 days before first antibody positive test in CIS before vaccination onwards** | **Exclude all visits from -35 days before the first positive from individuals ever tested PCR positive in the study onwards** | **Additionally, exclude all visits from -35 days before the first positive from individuals ever tested positive in the linked T&T data onwards** | **Additionally, exclude all visits from -35 days before first self-reporting positive swab and date onwards** | **Additionally, exclude all visits from individuals who report either loss of taste or loss of smell at their first visit and have no T&T result within**  **[-21,+21] days** | **Additionally, exclude**  **specific visits where participants report self-isolating and the previous and the next visit** | **Additionally, exclude**  **specific visits where participants report contact with definite positives in last 28 days and the previous and the next visit** | **Additionally, exclude (negative) visits occurring within [-7,+14 days] of either first or second vaccination date** |
| --- | --- | --- | --- | --- | --- | --- | --- | --- | --- |
| **Symptom** | **Number reporting this symptom after exclusions, i.e. remaining in analysis (% of those reporting this symptom in all PCR negative visits)** | | | | | | | | |
| **loss of smell** | 7,460 | 6,748 (90%) | 5,804 (78%) | 4,986 (67%) | 4,542 (61%) | 3,899 (52%) | 3,066 (41%) | 2,750 (37%) | 2,418 (32%) |
| **loss of taste** | 7,572 | 6,941 (92%) | 6,036 (80%) | 5,242 (69%) | 4,806 (63%) | 4,193 (55%) | 3,125 (41%) | 2,779 (37%) | 2,415 (32%) |
| **loss of taste or smell** | 10,655 | 9,782 (92%) | 8,563 (80%) | 7,545 (71%) | 6,968 (65%) | 6,103 (57%) | 4,746 (45%) | 4,248 (40%) | 3,706 (35%) |
| **abdominal pain** | 12,976 | 12,619 (97%) | 12,082 (93%) | 11,749 (91%) | 11,374 (88%) | 11,264 (87%) | 9,572 (74%) | 8,894 (69%) | 7,682 (59%) |
| **diarrhoea** | 13,335 | 12,961 (97%) | 12,454 (93%) | 12,126 (91%) | 11,753 (88%) | 11,635 (87%) | 9,921 (74%) | 9,210 (69%) | 7,936 (60%) |
| **nausea vomiting** | 14,100 | 13,671 (97%) | 13,074 (93%) | 12,631 (90%) | 12,250 (87%) | 12,127 (86%) | 10,228 (73%) | 9,439 (67%) | 7,328 (52%) |
| **shortness of breath** | 16,227 | 15,399 (95%) | 14,273 (88%) | 13,391 (83%) | 12,790 (79%) | 12,541 (77%) | 9,659 (60%) | 8,947 (55%) | 7,768 (48%) |
| **fever** | 16,616 | 16,147 (97%) | 15,486 (93%) | 14,975 (90%) | 14,427 (87%) | 14,296 (86%) | 11,363 (68%) | 10,480 (63%) | 7,034 (42%) |
| **muscle ache myalgia** | 26,621 | 25,623 (96%) | 24,229 (91%) | 23,271 (87%) | 22,360 (84%) | 22,114 (83%) | 18,604 (70%) | 17,266 (65%) | 11,636 (44%) |
| **fatigue weakness** | 40,220 | 38,540 (96%) | 36,214 (90%) | 34,603 (86%) | 33,276 (83%) | 32,907 (82%) | 27,549 (68%) | 25,367 (63%) | 18,704 (47%) |
| **sore throat** | 45,159 | 44,024 (97%) | 42,565 (94%) | 41,653 (92%) | 40,472 (90%) | 40,183 (89%) | 34,842 (77%) | 32,020 (71%) | 29,156 (65%) |
| **cough** | 46,561 | 45,176 (97%) | 43,292 (93%) | 41,936 (90%) | 40,519 (87%) | 40,185 (86%) | 33,641 (72%) | 30,920 (66%) | 28,428 (61%) |
| **headache** | 55,608 | 53,896 (97%) | 51,696 (93%) | 50,191 (90%) | 48,720 (88%) | 48,384 (87%) | 42,559 (77%) | 38,950 (70%) | 30,558 (55%) |
| **any evidence of symptoms** | 209,281 | 202,429 (97%) | 194,322 (93%) | 189,041 (90%) | 183,380 (88%) | 182,128 (87%) | 161,936 (77%) | 149,822 (72%) | 130,612 (62%) |

*Table S3 Characteristics of all and symptomatic positive episodes and all and symptomatic negative visits*

|  | **Positive episodes** | | **Negative visits** | |
| --- | --- | --- | --- | --- |
|  | **All positive episodes (N=27,869)** | **Symptomatic positive episodes (N=13,427)** | **All negative visits (N=3,806,692)** | **Symptomatic negative visits (N=130,612)** |
| Age (years) | 42 (22-58) | 42 (25-56) | 52 (32-66) | 48 (32-63) |
| 2-5 | 672 (2%) | 180 (1%) | 84,200 (2%) | 3,508 (3%) |
| 6-10 | 1,542 (6%) | 462 (3%) | 175,245 (5%) | 5,695 (4%) |
| 11-15 | 2,419 (9%) | 1,004 (7%) | 204,871 (5%) | 6,677 (5%) |
| 16-44 | 10,379 (37%) | 5,520 (41%) | 1,055,350 (28%) | 42,378 (32%) |
| 45-64 | 8,486 (30%) | 4,588 (34%) | 1,218,093 (32%) | 43,481 (33%) |
| 65+ | 4,371 (16%) | 1,673 (12%) | 1,068,933 (28%) | 28,873 (22%) |
| Male | 13,447 (48%) | 6,232 (46%) | 1,803,043 (47%) | 56,289 (43%) |
| Female | 14,422 (52%) | 7,195 (54%) | 2,003,649 (53%) | 74,323 (57%) |
| White | 24,877 (89%) | 12,116 (90%) | 3,534,865 (93%) | 122,251 (94%) |
| Asian/Asian British | 1,579 (6%) | 679 (5%) | 143,744 (4%) | 3,825 (3%) |
| Black/African/Caribbean/Black British | 406 (1%) | 156 (1%) | 33,418 (1%) | 930 (1%) |
| Mixed/Multiple ethnic groups | 689 (2%) | 312 (2%) | 64,276 (2%) | 2,544 (2%) |
| Other ethnic groups | 317 (1%) | 163 (1%) | 29,958 (1%) | 1,052 (1%) |
| Not yet vaccinated | 23,308 (84%) | 11,192 (83%) | 2,769,853 (73%) | 98,621 (76%) |
| ≥21 days from 1^st^ vaccine,  before 2^nd^ vaccine | 551 (2%) | 230 (2%) | 291,874 (8%) | 8,878 (7%) |
| ≥14 days from 2^nd^ vaccine | 1,969 (7%) | 1,010 (8%) | 557,849 (15%) | 16,753 (13%) |

| **Gene positivity pattern** | **All positive episodes** | | **Symptomatic positive episodes** | |
| --- | --- | --- | --- | --- |
|  | **Ct<30** | **Ct≥30** | **Ct<30** | **Ct≥30** |
| S-gene present before 17/Nov/2020 (wild-type) | 3,995 | 578 | 2,412 | 229 |
| S-gene absent from 17/Nov/2020 to 17/May/2020  (Alpha-compatible) | 6,324 | 2,561 | 3,900 | 849 |
| S-gene present from 17/May/2020 onwards (Delta-compatible) | 3,309 | 724 | 2,096 | 293 |

Note: showing median (IQR) or n (col %). Overall PPV of any evidence of symptoms predicting PCR positive episodes is 9%.

*Table S4 Actual UK population by age category, and in this study by month and by the three periods with different dominating variants: wild type, Alpha and Delta*

|  | **2-11 years** | **12-16 years** | **17-24 years** | **25-34 years** | **35-49 years** | **50-69 years** | **70+ years** | **Total** |
| --- | --- | --- | --- | --- | --- | --- | --- | --- |
| **Actual UK population by age** | 8,112,866 (12.6%) | 3,880,522 (6.0%) | 5,886,258 (9.1%) | 8,901,150 (13.8%) | 12,732,174 (19.7%) | 16,315,106 (25.2%) | 8,835,719 (13.7%) | 64,663,795 |
| **In this study** |  |  |  |  |  |  |  |  |
| **04/2020** | 131 (9.1%) | 90 (6.2%) | 100 (6.9%) | 146 (10.1%) | 299 (20.7%) | 518 (35.9%) | 158 (11.0%) | 1,442 |
| **05/2020** | 4,572 (10.2%) | 2,943 (6.6%) | 3,121 (7.0%) | 4,506 (10.0%) | 9,887 (22.0%) | 15,418 (34.3%) | 4,464 (9.9%) | 44,911 |
| **06/2020** | 6,371 (9.6%) | 4,013 (6.1%) | 4,101 (6.2%) | 5,859 (8.8%) | 13,130 (19.8%) | 23,379 (35.2%) | 9,497 (14.3%) | 66,350 |
| **07/2020** | 7,906 (8.8%) | 5,152 (5.7%) | 4,934 (5.5%) | 7,404 (8.3%) | 16,949 (18.9%) | 31,102 (34.7%) | 16,254 (18.1%) | 89,701 |
| **08/2020** | 10,923 (8.0%) | 7,283 (5.3%) | 7,517 (5.5%) | 12,025 (8.8%) | 25,411 (18.5%) | 47,652 (34.8%) | 26,290 (19.2%) | 137,101 |
| **09/2020** | 22,607 (8.1%) | 14,350 (5.1%) | 14,183 (5.1%) | 25,587 (9.1%) | 54,683 (19.5%) | 94,577 (33.8%) | 54,257 (19.4%) | 280,244 |
| **10/2020** | 34,510 (7.3%) | 21,041 (4.5%) | 23,016 (4.9%) | 46,029 (9.8%) | 91,649 (19.5%) | 162,114 (34.5%) | 91,775 (19.5%) | 470,134 |
| **11/2020** | 29,578 (7.4%) | 17,960 (4.5%) | 20,476 (5.1%) | 39,787 (10.0%) | 80,308 (20.2%) | 139,112 (34.9%) | 70,957 (17.8%) | 398,178 |
| **12/2020** | 22,766 (6.7%) | 14,427 (4.3%) | 16,040 (4.7%) | 32,662 (9.7%) | 67,155 (19.8%) | 121,395 (35.9%) | 64,123 (18.9%) | 338,568 |
| **01/2021** | 27,229 (8.2%) | 19,680 (5.9%) | 18,459 (5.5%) | 33,537 (10.0%) | 69,452 (20.8%) | 121,078 (36.2%) | 44,768 (13.4%) | 334,203 |
| **02/2021** | 27,037 (9.5%) | 20,446 (7.2%) | 18,424 (6.5%) | 31,629 (11.1%) | 64,502 (22.6%) | 86,795 (30.5%) | 36,119 (12.7%) | 284,952 |
| **03/2021** | 25,098 (8.9%) | 18,326 (6.5%) | 15,879 (5.7%) | 28,432 (10.1%) | 56,687 (20.2%) | 80,008 (28.5%) | 56,377 (20.1%) | 280,807 |
| **04/2021** | 21,027 (8.8%) | 15,248 (6.4%) | 13,284 (5.6%) | 23,698 (9.9%) | 44,941 (18.8%) | 87,739 (36.7%) | 33,027 (13.8%) | 238,964 |
| **05/2021** | 21,071 (8.3%) | 15,656 (6.2%) | 13,144 (5.2%) | 21,974 (8.7%) | 41,837 (16.5%) | 79,821 (31.5%) | 59,717 (23.6%) | 253,220 |
| **06/2021** | 19,180 (7.3%) | 13,808 (5.3%) | 9,774 (3.7%) | 16,739 (6.4%) | 44,736 (17.1%) | 91,968 (35.2%) | 65,003 (24.9%) | 261,208 |
| **07/2021** | 18,383 (6.3%) | 13,191 (4.5%) | 9,928 (3.4%) | 20,644 (7.1%) | 46,561 (16.0%) | 112,809 (38.8%) | 69,013 (23.8%) | 290,529 |
| **08/2021** | 3,723 (5.8%) | 2,799 (4.4%) | 2,258 (3.5%) | 4,186 (6.5%) | 10,793 (16.9%) | 24,923 (38.9%) | 15,367 (24.0%) | 64,049 |
| **26/04/2020-16/11/2020** | 101,795 (7.9%) | 63,846 (4.9%) | 67,161 (5.2%) | 121,811 (9.4%) | 252,455 (19.5%) | 445,797 (34.5%) | 239,528 (18.5%) | 1,292,393 |
| **17/11/2020-16/05/2021** | 148,796 (8.3%) | 105,081 (5.8%) | 99,362 (5.5%) | 181,902 (10.1%) | 364,336 (20.2%) | 606,562 (33.7%) | 295,639 (16.4%) | 1,801,678 |
| **16/05/2021-07/08/2021** | 51,521 (7.0%) | 37,486 (5.1%) | 28,115 (3.8%) | 51,131 (6.9%) | 122,189 (16.5%) | 268,049 (36.2%) | 181,999 (24.6%) | 740,490 |

Note: Actual UK population by age category taken from <https://www.ons.gov.uk/peoplepopulationandcommunity/healthandsocialcare/conditionsanddiseases/methodologies/coronaviruscovid19infectionsurveyqmi>

*Table S5 Actual UK population by sex, and in this study by month and by the three periods with different dominating variants, wild type, Alpha and Delta*

|  | **Male** | **Female** | **Male percentage** | **Female percentage** |
| --- | --- | --- | --- | --- |
| **Actual UK population by sex** | 31,190,622 | 32,753,171 | 48.8% | 51.2% |
| **04/2020** | 715 | 727 | 49.6% | 50.4% |
| **05/2020** | 21,868 | 23,043 | 48.7% | 51.3% |
| **06/2020** | 32,294 | 34,056 | 48.7% | 51.3% |
| **07/2020** | 43,169 | 46,532 | 48.1% | 51.9% |
| **08/2020** | 65,463 | 71,638 | 47.7% | 52.3% |
| **09/2020** | 133,378 | 146,866 | 47.6% | 52.4% |
| **10/2020** | 222,738 | 247,396 | 47.4% | 52.6% |
| **11/2020** | 189,273 | 208,905 | 47.5% | 52.5% |
| **12/2020** | 160,187 | 178,381 | 47.3% | 52.7% |
| **01/2021** | 160,288 | 173,915 | 48% | 52% |
| **02/2021** | 135,928 | 149,024 | 47.7% | 52.3% |
| **03/2021** | 133,631 | 147,176 | 47.6% | 52.4% |
| **04/2021** | 112,870 | 126,094 | 47.2% | 52.8% |
| **05/2021** | 118,822 | 134,398 | 46.9% | 53.1% |
| **06/2021** | 121,364 | 139,844 | 46.5% | 53.5% |
| **07/2021** | 134,851 | 155,678 | 46.4% | 53.6% |
| **08/2021** | 29,651 | 34,398 | 46.3% | 53.7% |
| **26/04/2020-16/11/2020** | 615,884 | 676,509 | 47.7% | 52.3% |
| **17/11/2020-16/05/2021** | 856,028 | 945,650 | 47.5% | 52.5% |
| **16/05/2021-07/08/2021** | 344,578 | 395,912 | 46.5% | 53.5% |

Note: Actual UK population by sex taken from <https://www.ons.gov.uk/peoplepopulationandcommunity/healthandsocialcare/conditionsanddiseases/methodologies/coronaviruscovid19infectionsurveyqmi>

*Table S6 Actual UK population by ethnicity, and in this study by month and by the three periods with different dominating variants: wild type, Alpha and Delta*

|  | **White** | **Asian/Asian British** | **Black/African/Caribbean/Black British** | **Mixed/Multiple ethnic groups** | **Other ethnic groups** | **Total** |
| --- | --- | --- | --- | --- | --- | --- |
| **Actual UK population by ethnicity** | 55,513,974 (85.9%) | 4,791,530 (7.4%) | 2,150,975 (3.3%) | 1,064,744 (1.6%) | 1,142,570 (1.8%) | 64,663,793 |
| **In this study** |  |  |  |  |  |  |
| **04/2020** | 1,359 (94.4%) | 55 (3.8%) | 10 (0.7%) | 13 (0.9%) | 2 (0.1%) | 1,439 |
| **05/2020** | 42,181 (94.1%) | 1,341 (3%) | 366 (0.8%) | 733 (1.6%) | 219 (0.5%) | 44,840 |
| **06/2020** | 62,593 (94.5%) | 1,767 (2.7%) | 518 (0.8%) | 1,042 (1.6%) | 334 (0.5%) | 66,254 |
| **07/2020** | 84,270 (94%) | 2,799 (3.1%) | 727 (0.8%) | 1,460 (1.6%) | 397 (0.4%) | 89,653 |
| **08/2020** | 127,601 (93.1%) | 5,011 (3.7%) | 1,294 (0.9%) | 2,264 (1.7%) | 862 (0.6%) | 137,032 |
| **09/2020** | 259,592 (92.7%) | 10,988 (3.9%) | 2,588 (0.9%) | 4,815 (1.7%) | 2,202 (0.8%) | 280,185 |
| **10/2020** | 435,193 (92.6%) | 18,870 (4%) | 4,139 (0.9%) | 7,855 (1.7%) | 4,026 (0.9%) | 470,083 |
| **11/2020** | 369,459 (92.8%) | 15,247 (3.8%) | 3,388 (0.9%) | 6,740 (1.7%) | 3,335 (0.8%) | 398,169 |
| **12/2020** | 315,216 (93.1%) | 12,472 (3.7%) | 2,823 (0.8%) | 5,344 (1.6%) | 2,709 (0.8%) | 338,564 |
| **01/2021** | 310,942 (93%) | 11,954 (3.6%) | 2,739 (0.8%) | 5,765 (1.7%) | 2,795 (0.8%) | 334,195 |
| **02/2021** | 262,584 (92.2%) | 11,635 (4.1%) | 2,657 (0.9%) | 5,617 (2%) | 2,458 (0.9%) | 284,951 |
| **03/2021** | 260,960 (92.9%) | 10,357 (3.7%) | 2,276 (0.8%) | 4,946 (1.8%) | 2,266 (0.8%) | 280,805 |
| **04/2021** | 221,441 (92.7%) | 9,226 (3.9%) | 2,110 (0.9%) | 4,337 (1.8%) | 1,848 (0.8%) | 238,962 |
| **05/2021** | 234,473 (92.6%) | 9,967 (3.9%) | 2,401 (0.9%) | 4,356 (1.7%) | 2,022 (0.8%) | 253,219 |
| **06/2021** | 242,147 (92.7%) | 10,213 (3.9%) | 2,515 (1%) | 4,237 (1.6%) | 2,091 (0.8%) | 261,203 |
| **07/2021** | 269,946 (92.9%) | 11,129 (3.8%) | 2,721 (0.9%) | 4,463 (1.5%) | 2,268 (0.8%) | 290,527 |
| **08/2021** | 59,785 (93.3%) | 2,292 (3.6%) | 552 (0.9%) | 978 (1.5%) | 441 (0.7%) | 64,048 |
| **26/04/2020-16/11/2020** | 1,200,702 (92.9%) | 48,705 (3.8%) | 11,325 (0.9%) | 21,536 (1.7%) | 9,725 (0.8%) | 1,291,993 |
| **17/11/2020-16/05/2021** | 1,671,579 (92.8%) | 68,116 (3.8%) | 15,505 (0.9%) | 31,701 (1.8%) | 14,754 (0.8%) | 1,801,655 |
| **16/05/2021-07/08/2021** | 687,461 (92.8%) | 28,502 (3.8%) | 6,994 (0.9%) | 11,728 (1.6%) | 5,796 (0.8%) | 740,481 |

Note: Actual UK population by ethnicity taken from <https://www.ons.gov.uk/peoplepopulationandcommunity/healthandsocialcare/conditionsanddiseases/methodologies/coronaviruscovid19infectionsurveyqmi>

*Table S7 Sensitivity, specificity, test per case (TPC), AUROC and inflation factor for various symptom combinations across the whole study period*

| **Criteria** | **Symptoms** | **sensitivity** | **specificity** | **AUROC** | **TPC** | **inflation** |
| --- | --- | --- | --- | --- | --- | --- |
| ***All positives*** | | | | | | |
| **Any of the 12 elicited symptoms** | Any one of the 12 elicited symptoms | 90 | 29 | 0.593 | 8.7 | 2.3 |
| **4 classic symptoms** | *Fever or cough or loss of taste or loss of smell* | 74 | 73 | 0.734 | 4.6 | 1 |
| **4 classic symptoms + fatigue/weakness**** | *Fever or cough or loss of taste or loss of smell or* **fatigue/weakness** | 81 | 64 | 0.727 | 5.3 | 1.3 |
| **4 classic symptoms + fatigue/weakness + muscle ache/mylagia**** | *Fever or cough or loss of taste or loss of smell or* **fatigue/weakness** *or* **muscle ache/myalgia** | 83 | 60 | 0.716 | 5.7 | 1.4 |
| **4 symptoms: highest sensitivity** | *loss of smell or cough or* **fatigue/weakness** *or* **headache** | 83 | 52 | 0.675 | 6.6 | 1.6 |
| **4 symptoms: highest AUROC** | *loss of taste or loss of smell or fever or* **fatigue/weakness** | 71 | 81 | 0.759 | 3.6 | 0.8 |
| **4 symptoms: lowest TPC** | *loss of taste or loss of smell or fever or* **shortness of breath** | 61 | 87 | 0.741 | 3 | 0.5 |
| **4 symptoms: lowest inflation factor** | *loss of taste or loss of smell or* **abdominal pain** *or* **nausea/vomiting** | 49 | 88 | 0.682 | 3.5 | 0.5 |
| **1 symptom: highest sensitivity** | **fatigue/weakness** | 54 | 86 | 0.7 | 3.6 | 0.6 |
| **1 symptom: highest AUROC** | **fatigue/weakness** | 54 | 86 | 0.7 | 3.6 | 0.6 |
| **1 symptom: lowest TPC** | *loss of taste* | 33 | 98 | 0.658 | 1.5 | 0.2 |
| **1 symptom: lowest inflation factor** | *loss of smell* | 33 | 98 | 0.654 | 1.6 | 0.2 |
| **2 symptoms: highest sensitivity** | *cough or* **headache** | 72 | 59 | 0.655 | 6.5 | 1.4 |
| **2 symptoms: highest AUROC** | *loss of smell or* **fatigue/weakness** | 64 | 84 | 0.742 | 3.4 | 0.6 |
| **2 symptoms: lowest TPC** | *loss of taste or loss of smell* | 39 | 97 | 0.683 | 1.7 | 0.2 |
| **2 symptoms: lowest inflation factor** | *loss of taste or loss of smell* | 39 | 97 | 0.683 | 1.7 | 0.2 |
| **3 symptoms: highest sensitivity** | *cough or* **fatigue/weakness** *or* **headache** | 79 | 53 | 0.659 | 6.8 | 1.6 |
| **3 symptoms: highest AUROC** | *loss of smell or fever or* **fatigue/weakness** | 70 | 81 | 0.754 | 3.6 | 0.7 |
| **3 symptoms: lowest TPC** | *loss of taste or loss of smell or fever* | 55 | 92 | 0.737 | 2.4 | 0.4 |
| **3 symptoms: lowest inflation factor** | *loss of taste or loss of smell or* **nausea/vomiting** | 46 | 92 | 0.689 | 2.7 | 0.4 |
| **5 symptoms: highest sensitivity** | *loss of smell or cough or* **fatigue/weakness** *or* **headache** *or* **sore throat** | 86 | 41 | 0.632 | 7.7 | 2 |
| **5 symptoms: highest AUROC** | *loss of taste or loss of smell or fever or* **fatigue/weakness** *or* **muscle ache/myalgia** | 74 | 77 | 0.752 | 4.1 | 0.9 |
| **5 symptoms: lowest TPC** | *loss of taste or loss of smell or fever or* **shortness of breath** *or* **nausea/vomiting** | 63 | 83 | 0.732 | 3.6 | 0.7 |
| **5 symptoms: lowest inflation factor** | *loss of taste or loss of smell or* **abdominal pain** *or* **diarrhoea** *or* **nausea/vomiting** | 51 | 84 | 0.677 | 4 | 0.6 |
| **6 symptoms: highest sensitivity** | *loss of smell or fever or cough or* **fatigue/weakness** *or* **headache** *or* **sore throat** | 87 | 39 | 0.632 | 7.8 | 2 |
| **6 symptoms: highest AUROC** | *loss of taste or loss of smell or fever or* **fatigue/weakness** *or* **muscle ache/myalgia** *or* **shortness of breath** | 75 | 74 | 0.745 | 4.4 | 1 |
| **6 symptoms: lowest TPC** | *loss of taste or loss of smell or fever or* **shortness of breath** *or* **abdominal pain** *or* **nausea/vomiting** | 64 | 80 | 0.721 | 4.1 | 0.8 |
| **6 symptoms: lowest inflation factor** | *loss of taste or loss of smell or fever or* **abdominal pain** *or* **diarrhoea** *or* **nausea/vomiting** | 62 | 80 | 0.709 | 4.1 | 0.8 |
| **7 symptoms: highest sensitivity** | *loss of smell or fever or cough or* **fatigue/weakness** *or* **muscle ache/myalgia** *or* **headache** *or* **sore throat** | 88 | 37 | 0.623 | 8 | 2.1 |
| **7 symptoms: highest AUROC** | *loss of taste or loss of smell or fever or* **fatigue/weakness** *or* **muscle ache/myalgia** *or* **shortness of breath** *or* **nausea/vomiting** | 76 | 71 | 0.734 | 4.7 | 1.1 |
| **7 symptoms: lowest TPC** | *loss of taste or loss of smell or fever or* **shortness of breath** *or* **abdominal pain** *or* **diarrhoea** *or* **nausea/vomiting** | 66 | 76 | 0.71 | 4.5 | 0.9 |
| **7 symptoms: lowest inflation factor** | *loss of taste or loss of smell or fever or* **shortness of breath** *or* **abdominal pain** *or* **diarrhoea** *or* **nausea/vomiting** | 66 | 76 | 0.71 | 4.5 | 0.9 |
| ***Ct<30*** | | | | | | |
| **Any of the 12 elicited symptoms** | Any one of the 12 elicited symptoms | 92 | 29 | 0.604 | 11 | 2.4 |
| **4 classic symptoms** | *Fever or cough or loss of taste or loss of smell* | 78 | 73 | 0.756 | 5.5 | 1 |
| **4 classic symptoms + fatigue/weakness**** | *Fever or cough or loss of taste or loss of smell or* **fatigue/weakness** | 85 | 64 | 0.744 | 6.5 | 1.3 |
| **4 classic symptoms + fatigue/weakness + muscle ache/mylagia**** | *Fever or cough or loss of taste or loss of smell or* **fatigue/weakness** *or* **muscle ache/myalgia** | 86 | 60 | 0.733 | 7 | 1.4 |
| **4 symptoms: highest sensitivity** | *loss of smell or cough or* **fatigue/weakness** *or* **headache** | 86 | 52 | 0.689 | 8.3 | 1.7 |
| **4 symptoms: highest AUROC** | *loss of taste or loss of smell or fever or* **fatigue/weakness** | 75 | 81 | 0.778 | 4.4 | 0.8 |
| **4 symptoms: lowest TPC** | *loss of taste or loss of smell or fever or* **shortness of breath** | 65 | 87 | 0.76 | 3.5 | 0.5 |
| **4 symptoms: lowest inflation factor** | *loss of taste or loss of smell or* **abdominal pain** *or* ***nausea/vomiting*** | 51 | 88 | 0.695 | 4.1 | 0.5 |
| **1 symptom: highest sensitivity** | *cough* | 58 | 78 | 0.681 | 5.9 | 0.8 |
| **1 symptom: highest AUROC** | **fatigue/weakness** | 57 | 86 | 0.715 | 4.2 | 0.6 |
| **1 symptom: lowest TPC** | *loss of taste* | 35 | 98 | 0.667 | 1.7 | 0.1 |
| **1 symptom: lowest inflation factor** | *loss of smell* | 34 | 98 | 0.66 | 1.7 | 0.1 |
| **2 symptoms: highest sensitivity** | *cough or* **headache** | 76 | 59 | 0.674 | 8 | 1.4 |
| **2 symptoms: highest AUROC** | *loss of smell or* ***fatigue/weakness*** | 67 | 84 | 0.756 | 4 | 0.6 |
| **2 symptoms: lowest TPC** | *loss of taste or loss of smell* | 41 | 97 | 0.692 | 1.9 | 0.2 |
| **2 symptoms: lowest inflation factor** | *loss of taste or loss of smell* | 41 | 97 | 0.692 | 1.9 | 0.2 |
| **3 symptoms: highest sensitivity** | *cough or* **fatigue/weakness** *or* **headache** | 82 | 53 | 0.675 | 8.5 | 1.6 |
| **3 symptoms: highest AUROC** | *loss of smell or fever or* **fatigue/weakness** | 73 | 81 | 0.773 | 4.3 | 0.7 |
| **3 symptoms: lowest TPC** | *loss of taste or loss of smell or fever* | 60 | 92 | 0.759 | 2.7 | 0.4 |
| **3 symptoms: lowest inflation factor** | *loss of taste or loss of smell or* **nausea/vomiting** | 48 | 92 | 0.702 | 3.2 | 0.4 |
| **5 symptoms: highest sensitivity** | *loss of smell or cough or* **fatigue/weakness** *or* **headache** *or* **sore throat** | 88 | 41 | 0.645 | 9.7 | 2 |
| **5 symptoms: highest AUROC** | *loss of taste or loss of smell or fever or* **fatigue/weakness** *or* **muscle ache/myalgia** | 77 | 77 | 0.77 | 4.9 | 0.9 |
| **5 symptoms: lowest TPC** | *loss of taste or loss of smell or fever or* **shortness of breath** *or* **nausea/vomiting** | 67 | 83 | 0.751 | 4.3 | 0.7 |
| **5 symptoms: lowest inflation factor** | *loss of taste or loss of smell or* **abdominal pain** *or* **diarrhoea** *or* **nausea/vomiting** | 54 | 84 | 0.69 | 4.8 | 0.6 |
| **6 symptoms: highest sensitivity** | *loss of smell or fever or cough or* **fatigue/weakness** *or* **headache** *or* **sore throat** | 90 | 39 | 0.646 | 9.8 | 2.1 |
| **6 symptoms: highest AUROC** | *loss of taste or loss of smell or fever or* **fatigue/weakness** *or* **muscle ache/myalgia** *or* **shortness of breath** | 79 | 74 | 0.762 | 5.3 | 1 |
| **6 symptoms: lowest TPC** | *loss of taste or loss of smell or fever or* **abdominal pain** *or* **diarrhoea** *or* **nausea/vomiting** | 66 | 80 | 0.731 | 4.9 | 0.7 |
| **6 symptoms: lowest inflation factor** | *loss of taste or loss of smell or fever or* **abdominal pain** *or* **diarrhoea** *or* **nausea/vomiting** | 66 | 80 | 0.731 | 4.9 | 0.7 |
| **7 symptoms: highest sensitivity** | *loss of taste or loss of smell or fever or cough or* **fatigue/weakness** *or* **headache** *or* **sore throat** | 91 | 39 | 0.647 | 9.8 | 2.1 |
| **7 symptoms: highest AUROC** | *loss of taste or loss of smell or fever or* **fatigue/weakness** *or* **muscle ache/myalgia** *or* **shortness of breath** *or* **nausea/vomiting** | 79 | 71 | 0.751 | 5.7 | 1.1 |
| **7 symptoms: lowest TPC** | *loss of taste or loss of smell or fever or* **shortness of breath** *or* **abdominal pain** *or* **diarrhoea** *or* **nausea/vomiting** | 69 | 76 | 0.729 | 5.4 | 0.9 |
| **7 symptoms: lowest inflation factor** | *loss of taste or loss of smell or fever or* **shortness of breath** *or* **abdominal pain** *or* **diarrhoea** *or* **nausea/vomiting** | 69 | 76 | 0.729 | 5.4 | 0.9 |
| ***Ct≥30*** | | | | | | |
| **Any of the 12 elicited symptoms** | Any one of the 12 elicited symptoms | 83 | 29 | 0.559 | 34.6 | 2.6 |
| **4 classic symptoms** | *Fever or cough or loss of taste or loss of smell* | 60 | 73 | 0.667 | 18.5 | 1 |
| **4 classic symptoms + fatigue/weakness**** | *Fever or cough or loss of taste or loss of smell or* **fatigue/weakness** | 71 | 64 | 0.675 | 20.9 | 1.3 |
| **4 classic symptoms + fatigue/weakness + muscle ache/mylagia**** | *Fever or cough or loss of taste or loss of smell or* **fatigue/weakness** *or* **muscle ache/myalgia** | 73 | 60 | 0.667 | 22.2 | 1.5 |
| **4 symptoms: highest sensitivity** | *loss of smell or cough or* **fatigue/weakness** *or* **headache** | 75 | 52 | 0.634 | 26.1 | 1.8 |
| **4 symptoms: highest AUROC** | *loss of taste or loss of smell or* **fatigue/weakness** *or* **shortness of breath** | 60 | 81 | 0.704 | 13.6 | 0.7 |
| **4 symptoms: lowest TPC** | *loss of taste or loss of smell or fever or* **shortness of breath** | 50 | 87 | 0.688 | 10.9 | 0.5 |
| **4 symptoms: lowest inflation factor** | *loss of taste or loss of smell or* **abdominal pain** *or* **nausea/vomiting** | 41 | 88 | 0.644 | 12.7 | 0.5 |
| **1 symptom: highest sensitivity** | **fatigue/weakness** | 45 | 86 | 0.653 | 13.5 | 0.5 |
| **1 symptom: highest AUROC** | **fatigue/weakness** | 45 | 86 | 0.653 | 13.5 | 0.5 |
| **1 symptom: lowest TPC** | *loss of smell* | 29 | 98 | 0.634 | 3.5 | 0.1 |
| **1 symptom: lowest inflation factor** | *loss of taste* | 28 | 98 | 0.63 | 3.6 | 0.1 |
| **2 symptoms: highest sensitivity** | *cough or* **fatigue/weakness** | 61 | 68 | 0.643 | 21.8 | 1.2 |
| **2 symptoms: highest AUROC** | *loss of smell or* **fatigue/weakness** | 55 | 84 | 0.698 | 12 | 0.6 |
| **2 symptoms: lowest TPC** | *loss of taste or loss of smell* | 34 | 97 | 0.656 | 4.3 | 0.1 |
| **2 symptoms: lowest inflation factor** | *loss of taste or loss of smell* | 34 | 97 | 0.656 | 4.3 | 0.1 |
| **3 symptoms: highest sensitivity** | *cough or* **fatigue/weakness** *or* **headache** | 70 | 53 | 0.612 | 27.6 | 1.7 |
| **3 symptoms: highest AUROC** | *loss of taste or loss of smell or* **fatigue/weakness** | 57 | 84 | 0.704 | 12.1 | 0.6 |
| **3 symptoms: lowest TPC** | *loss of taste or loss of smell or* **shortness of breath** | 45 | 92 | 0.682 | 8.2 | 0.3 |
| **3 symptoms: lowest inflation factor** | *loss of taste or loss of smell or fever* | 42 | 92 | 0.672 | 8.2 | 0.3 |
| **5 symptoms: highest sensitivity** | *loss of smell or cough or* **fatigue/weakness** *or* **headache** *or* **sore throat** | 78 | 41 | 0.593 | 30.8 | 2.1 |
| **5 symptoms: highest AUROC** | *loss of taste or loss of smell or fever or* **fatigue/weakness** *or* **shortness of breath** | 63 | 78 | 0.701 | 15 | 0.8 |
| **5 symptoms: lowest TPC** | *loss of taste or loss of smell or fever or* **shortness of breath** *or* **nausea/vomiting** | 52 | 83 | 0.676 | 13.6 | 0.6 |
| **5 symptoms: lowest inflation factor** | *loss of taste or loss of smell or* **abdominal pain** *or* **diarrhoea** *or* **nausea/vomiting** | 44 | 84 | 0.638 | 15.3 | 0.6 |
| **6 symptoms: highest sensitivity** | *loss of smell or cough or* **fatigue/weakness** *or* **headache** *or* **shortness of breath** *or* **sore throat** | 79 | 39 | 0.591 | 31.2 | 2.2 |
| **6 symptoms: highest AUROC** | *loss of taste or loss of smell or fever or* **fatigue/weakness** *or* **muscle ache/myalgia** *or* **shortness of breath** | 65 | 74 | 0.696 | 16.7 | 1 |
| **6 symptoms: lowest TPC** | *loss of taste or loss of smell or fever or* **shortness of breath** *or* **abdominal pain** *or* **nausea/vomiting** | 53 | 80 | 0.665 | 16 | 0.8 |
| **6 symptoms: lowest inflation factor** | *loss of taste or loss of smell or fever or* **abdominal pain** *or* **diarrhoea** *or* **nausea/vomiting** | 49 | 80 | 0.646 | 16.8 | 0.7 |
| **7 symptoms: highest sensitivity** | *loss of smell or cough or* **fatigue/weakness** *or* **muscle ache/myalgia** *or* **headache** *or* **shortness of breath** *or* **sore throat** | 80 | 37 | 0.584 | 31.9 | 2.3 |
| **7 symptoms: highest AUROC** | *loss of taste or loss of smell or fever or* **fatigue/weakness** *or* **muscle ache/myalgia** *or* **shortness of breath** *or* **nausea/vomiting** | 66 | 71 | 0.685 | 18.2 | 1.1 |
| **7 symptoms: lowest TPC** | *loss of taste or loss of smell or fever or* **shortness of breath** *or* **abdominal pain** *or* **diarrhoea** *or* **nausea/vomiting** | 55 | 76 | 0.657 | 17.8 | 0.9 |
| **7 symptoms: lowest inflation factor** | *loss of taste or loss of smell or fever or* **shortness of breath** *or* **abdominal pain** *or* **diarrhoea** *or* **nausea/vomiting** | 55 | 76 | 0.657 | 17.8 | 0.9 |
| ***Wild type, Ct<30*** | | | | | | |
| **Any of the 12 elicited symptoms** | Any one of the 12 elicited symptoms | 85 | 53 | 0.691 | 10.3 | 1.9 |
| **4 classic symptoms** | *Fever or cough or loss of taste or loss of smell* | 74 | 78 | 0.759 | 6.1 | 1 |
| **4 classic symptoms + fatigue/weakness**** | *Fever or cough or loss of taste or loss of smell or* **fatigue/weakness** | 80 | 72 | 0.76 | 6.9 | 1.2 |
| **4 classic symptoms + fatigue/weakness + muscle ache/mylagia**** | *Fever or cough or loss of taste or loss of smell or* **fatigue/weakness** *or* **muscle ache/myalgia** | 81 | 71 | 0.76 | 7.1 | 1.3 |
| **4 symptoms: highest sensitivity** | *loss of smell or cough or* **fatigue/weakness** *or* **headache** | 80 | 68 | 0.74 | 7.7 | 1.4 |
| **4 symptoms: highest AUROC** | *loss of taste or loss of smell or fever or* **fatigue/weakness** | 71 | 87 | 0.788 | 4.1 | 0.6 |
| **4 symptoms: lowest TPC** | *loss of taste or loss of smell or* **diarrhoea** *or* **nausea/vomiting** | 52 | 94 | 0.727 | 3 | 0.3 |
| **4 symptoms: lowest inflation factor** | *loss of taste or loss of smell or* **abdominal pain** *or* **diarrhoea** | 49 | 94 | 0.717 | 3.1 | 0.3 |
| **1 symptom: highest sensitivity** | **fatigue/weakness** | 50 | 91 | 0.704 | 4.1 | 0.5 |
| **1 symptom: highest AUROC** | **fatigue/weakness** | 50 | 91 | 0.704 | 4.1 | 0.5 |
| **1 symptom: lowest TPC** | *loss of smell* | 36 | 99 | 0.676 | 1.5 | 0.1 |
| **1 symptom: lowest inflation factor** | *loss of smell* | 36 | 99 | 0.676 | 1.5 | 0.1 |
| **2 symptoms: highest sensitivity** | *cough or* **fatigue/weakness** | 68 | 75 | 0.712 | 7.3 | 1.1 |
| **2 symptoms: highest AUROC** | *loss of smell or* **fatigue/weakness** | 62 | 90 | 0.761 | 3.7 | 0.5 |
| **2 symptoms: lowest TPC** | *loss of taste or loss of smell* | 44 | 98 | 0.71 | 1.6 | 0.2 |
| **2 symptoms: lowest inflation factor** | *loss of taste or loss of smell* | 44 | 98 | 0.71 | 1.6 | 0.2 |
| **3 symptoms: highest sensitivity** | *loss of smell or cough or* **headache** | 76 | 72 | 0.739 | 7.2 | 1.2 |
| **3 symptoms: highest AUROC** | *loss of smell or fever or* **fatigue/weakness** | 69 | 87 | 0.779 | 4.2 | 0.6 |
| **3 symptoms: lowest TPC** | *loss of taste or loss of smell or* **nausea/vomiting** | 49 | 96 | 0.725 | 2.4 | 0.3 |
| **3 symptoms: lowest inflation factor** | *loss of taste or loss of smell or* **diarrhoea** | 47 | 96 | 0.715 | 2.5 | 0.3 |
| **5 symptoms: highest sensitivity** | *loss of smell or fever or cough or* **fatigue/weakness** *or* **headache** | 82 | 67 | 0.741 | 7.9 | 1.4 |
| **5 symptoms: highest AUROC** | *loss of taste or loss of smell or fever or* **fatigue/weakness** *or* **muscle ache/myalgia** | 74 | 85 | 0.792 | 4.5 | 0.7 |
| **5 symptoms: lowest TPC** | *loss of taste or loss of smell or* **abdominal pain** *or* **diarrhoea** *or* **nausea/vomiting** | 53 | 92 | 0.726 | 3.5 | 0.4 |
| **5 symptoms: lowest inflation factor** | *loss of taste or loss of smell or* **abdominal pain** *or* **diarrhoea** *or* **nausea/vomiting** | 53 | 92 | 0.726 | 3.5 | 0.4 |
| **6 symptoms: highest sensitivity** | *loss of smell or fever or cough or* **fatigue/weakness** *or* **headache** *or* **sore throat** | 83 | 57 | 0.701 | 9.7 | 1.8 |
| **6 symptoms: highest AUROC** | *loss of taste or loss of smell or fever or* **fatigue/weakness** *or* **muscle ache/myalgia** *or* **nausea/vomiting** | 74 | 84 | 0.79 | 4.7 | 0.8 |
| **6 symptoms: lowest TPC** | *loss of taste or loss of smell or fever or* **abdominal pain** *or* **diarrhoea** *or* **nausea/vomiting** | 63 | 89 | 0.76 | 4 | 0.6 |
| **6 symptoms: lowest inflation factor** | *loss of taste or loss of smell or* **shortness of breath** *or* **abdominal pain** *or* **diarrhoea** *or* **nausea/vomiting** | 57 | 89 | 0.733 | 4.1 | 0.5 |
| **7 symptoms: highest sensitivity** | *loss of taste or loss of smell or fever or cough or* **fatigue/weakness** *or* **headache** *or* **sore throat** | 84 | 57 | 0.706 | 9.6 | 1.8 |
| **7 symptoms: highest AUROC** | *loss of taste or loss of smell or fever or* **fatigue/weakness** *or* **muscle ache/myalgia** *or* **abdominal pain** *or* **nausea/vomiting** | 75 | 82 | 0.785 | 5 | 0.8 |
| **7 symptoms: lowest TPC** | *loss of taste or loss of smell or fever or* **muscle ache/myalgia** *or* **abdominal pain** *or* **diarrhoea** *or* **nausea/vomiting** | 69 | 86 | 0.774 | 4.5 | 0.7 |
| **7 symptoms: lowest inflation factor** | *loss of taste or loss of smell or fever or* **shortness of breath** *or* **abdominal pain** *or* **diarrhoea** *or* **nausea/vomiting** | 66 | 86 | 0.76 | 4.5 | 0.7 |
| ***Alpha variant compatible, Ct<30*** | | | | | | |
| **Any of the 12 elicited symptoms** | Any one of the 12 elicited symptoms | 95 | 20 | 0.578 | 15 | 2.7 |
| **4 classic symptoms** | *Fever or cough or loss of taste or loss of smell* | 79 | 73 | 0.761 | 6.6 | 1 |
| **4 classic symptoms + fatigue/weakness**** | *Fever or cough or loss of taste or loss of smell or* **fatigue/weakness** | 87 | 63 | 0.749 | 8.2 | 1.4 |
| **4 classic symptoms + fatigue/weakness + muscle ache/mylagia**** | *Fever or cough or loss of taste or loss of smell or* **fatigue/weakness** *or* **muscle ache/myalgia** | 89 | 58 | 0.735 | 8.9 | 1.5 |
| **4 symptoms: highest sensitivity** | *cough or* **fatigue/weakness** *or* **headache** *or* **sore throat** | 89 | 36 | 0.621 | 13.2 | 2.2 |
| **4 symptoms: highest AUROC** | *loss of taste or loss of smell or fever or* **fatigue/weakness** | 76 | 79 | 0.775 | 5.6 | 0.8 |
| **4 symptoms: lowest TPC** | *loss of taste or loss of smell or fever or* **shortness of breath** | 63 | 87 | 0.749 | 4.5 | 0.5 |
| **4 symptoms: lowest inflation factor** | *loss of taste or loss of smell or fever or* **nausea/vomiting** | 60 | 87 | 0.737 | 4.6 | 0.5 |
| **1 symptom: highest sensitivity** | *cough* | 61 | 79 | 0.703 | 6.7 | 0.8 |
| **1 symptom: highest AUROC** | **fatigue/weakness** | 61 | 84 | 0.726 | 5.3 | 0.6 |
| **1 symptom: lowest TPC** | *loss of taste* | 31 | 98 | 0.645 | 2.1 | 0.1 |
| **1 symptom: lowest inflation factor** | *loss of smell* | 28 | 98 | 0.632 | 2.2 | 0.1 |
| **2 symptoms: highest sensitivity** | *cough or* **fatigue/weakness** | 80 | 67 | 0.733 | 8 | 1.2 |
| **2 symptoms: highest AUROC** | *loss of taste or* **fatigue/weakness** | 68 | 83 | 0.754 | 5.2 | 0.7 |
| **2 symptoms: lowest TPC** | *loss of taste or loss of smell* | 36 | 97 | 0.666 | 2.5 | 0.2 |
| **2 symptoms: lowest inflation factor** | *loss of taste or loss of smell* | 36 | 97 | 0.666 | 2.5 | 0.2 |
| **3 symptoms: highest sensitivity** | *cough or* **fatigue/weakness** *or* **headache** | 86 | 47 | 0.664 | 11.4 | 1.9 |
| **3 symptoms: highest AUROC** | *loss of taste or fever or* **fatigue/weakness** | 74 | 80 | 0.772 | 5.5 | 0.8 |
| **3 symptoms: lowest TPC** | *loss of taste or loss of smell or fever* | 57 | 92 | 0.744 | 3.3 | 0.4 |
| **3 symptoms: lowest inflation factor** | *loss of taste or loss of smell or fever* | 57 | 92 | 0.744 | 3.3 | 0.4 |
| **5 symptoms: highest sensitivity** | *loss of taste or cough or* **fatigue/weakness** *or* **headache** *or* **sore throat** | 91 | 35 | 0.628 | 13 | 2.3 |
| **5 symptoms: highest AUROC** | *loss of taste or loss of smell or fever or fatigue/weakness or* **shortness of breath** | 77 | 76 | 0.765 | 6.3 | 0.9 |
| **5 symptoms: lowest TPC** | *loss of taste or loss of smell or fever or* **shortness of breath** *or* **nausea/vomiting** | 66 | 82 | 0.739 | 5.6 | 0.7 |
| **5 symptoms: lowest inflation factor** | *loss of taste or loss of smell or fever or* **abdominal pain** *or* **nausea/vomiting** | 63 | 82 | 0.723 | 5.8 | 0.7 |
| **6 symptoms: highest sensitivity** | *loss of taste or fever or cough or* **fatigue/weakness** *or* **headache** *or* **sore throat** | 93 | 33 | 0.629 | 13.1 | 2.3 |
| **6 symptoms: highest AUROC** | *loss of taste or loss of smell or fever or* **fatigue/weakness** *or* **muscle ache/myalgia** *or* **shortness of breath** | 80 | 71 | 0.753 | 7.1 | 1.1 |
| **6 symptoms: lowest TPC** | *loss of taste or loss of smell or fever or* **shortness of breath** *or* **abdominal pain** *or* **nausea/vomiting** | 67 | 77 | 0.724 | 6.6 | 0.8 |
| **6 symptoms: lowest inflation factor** | *loss of taste or loss of smell or fever or* **abdominal pain** *or* **diarrhoea** *or* **nausea/vomiting** | 65 | 78 | 0.712 | 6.7 | 0.8 |
| **7 symptoms: highest sensitivity** | *loss of smell or fever or cough or* **fatigue/weakness** *or* **muscle ache/myalgia** *or* **headache** *or* **sore throat** | 93 | 30 | 0.617 | 13.5 | 2.4 |
| **7 symptoms: highest AUROC** | *loss of taste or loss of smell or fever or* **fatigue/weakness** *or* **muscle ache/myalgia** *or* **shortness of breath** *or* **nausea/vomiting** | 80 | 68 | 0.741 | 7.7 | 1.2 |
| **7 symptoms: lowest TPC** | *loss of taste or loss of smell or fever or* **shortness of breath** *or* **abdominal pain** *or* **diarrhoea** *or* **nausea/vomiting** | 69 | 74 | 0.712 | 7.4 | 1 |
| **7 symptoms: lowest inflation factor** | *loss of taste or loss of smell or fever or* **shortness of breath** *or* **abdominal pain** *or* **diarrhoea** *or* **nausea/vomiting** | 69 | 74 | 0.712 | 7.4 | 1 |
| ***Delta variant compatible, Ct<30*** | | | | | | |
| **Any of the 12 elicited symptoms** | Any one of the 12 elicited symptoms | 95 | 11 | 0.529 | 12.1 | 2.3 |
| **4 classic symptoms** | *Fever or cough or loss of taste or loss of smell* | 83 | 64 | 0.738 | 6.1 | 1 |
| **4 classic symptoms + fatigue/weakness**** | *Fever or cough or loss of taste or loss of smell or* **fatigue/weakness** | 88 | 53 | 0.705 | 7.3 | 1.3 |
| **4 classic symptoms + fatigue/weakness + muscle ache/mylagia**** | *Fever or cough or loss of taste or loss of smell or* **fatigue/weakness** *or* **muscle ache/myalgia** | 89 | 48 | 0.688 | 7.8 | 1.4 |
| **4 symptoms: highest sensitivity** | *loss of smell or cough or* **headache** *or* **sore throat** | 90 | 35 | 0.627 | 9.5 | 1.7 |
| **4 symptoms: highest AUROC** | *loss of taste or loss of smell or fever or* **muscle ache/myalgia** | 74 | 80 | 0.772 | 4.1 | 0.6 |
| **4 symptoms: lowest TPC** | *loss of taste or loss of smell or fever or* **abdominal pain** | 68 | 83 | 0.755 | 3.9 | 0.5 |
| **4 symptoms: lowest inflation factor** | *loss of taste or loss of smell or* **abdominal pain** *or* **nausea/vomiting** | 55 | 83 | 0.69 | 4.6 | 0.5 |
| **1 symptom: highest sensitivity** | *cough* | 64 | 71 | 0.676 | 6.3 | 0.8 |
| **1 symptom: highest AUROC** | **fatigue/weakness** | 60 | 81 | 0.705 | 4.7 | 0.6 |
| **1 symptom: lowest TPC** | *loss of smell* | 39 | 97 | 0.682 | 1.8 | 0.1 |
| **1 symptom: lowest inflation factor** | *loss of taste* | 38 | 97 | 0.678 | 1.8 | 0.1 |
| **2 symptoms: highest sensitivity** | *cough or* **headache** | 82 | 50 | 0.663 | 8.1 | 1.3 |
| **2 symptoms: highest AUROC** | *loss of smell or fever* | 64 | 90 | 0.77 | 2.8 | 0.3 |
| **2 symptoms: lowest TPC** | *loss of taste or loss of smell* | 46 | 96 | 0.709 | 2.1 | 0.2 |
| **2 symptoms: lowest inflation factor** | *loss of taste or loss of smell* | 46 | 96 | 0.709 | 2.1 | 0.2 |
| **3 symptoms: highest sensitivity** | *cough or* **headache** *or* **sore throat** | 87 | 36 | 0.616 | 9.6 | 1.7 |
| **3 symptoms: highest AUROC** | *loss of taste or loss of smell or fever* | 67 | 89 | 0.778 | 2.9 | 0.4 |
| **3 symptoms: lowest TPC** | *loss of taste or loss of smell or fever* | 67 | 89 | 0.778 | 2.9 | 0.4 |
| **3 symptoms: lowest inflation factor** | *loss of taste or loss of smell or* **abdominal pain** | 50 | 89 | 0.694 | 3.6 | 0.4 |
| **5 symptoms: highest sensitivity** | *loss of smell or fever or cough or* **headache** *or* **sore throat** | 93 | 33 | 0.628 | 9.5 | 1.7 |
| **5 symptoms: highest AUROC** | *loss of taste or loss of smell or fever or* **fatigue/weakness** *or* **muscle ache/myalgia** | 81 | 69 | 0.754 | 5.4 | 0.9 |
| **5 symptoms: lowest TPC** | *loss of taste or loss of smell or fever or* **abdominal pain** *or* **nausea/vomiting** | 70 | 78 | 0.742 | 4.7 | 0.6 |
| **5 symptoms: lowest inflation factor** | *loss of taste or loss of smell or* **abdominal pain** *or* **diarrhoea** *or* **nausea/vomiting** | 56 | 78 | 0.673 | 5.6 | 0.6 |
| **6 symptoms: highest sensitivity** | *loss of smell or fever or cough or* **fatigue/weakness** *or* **headache** *or* **sore throat** | 94 | 26 | 0.6 | 10.3 | 1.9 |
| **6 symptoms: highest AUROC** | *loss of taste or loss of smell or fever or* **fatigue/weakness** *or* **muscle ache/myalgia** *or* **shortness of breath** | 82 | 65 | 0.738 | 6 | 1 |
| **6 symptoms: lowest TPC** | *loss of taste or loss of smell or fever or* **muscle ache/myalgia** *or* **abdominal pain** *or* **nausea/vomiting** | 76 | 71 | 0.737 | 5.5 | 0.8 |
| **6 symptoms: lowest inflation factor** | *loss of taste or loss of smell or fever or* **abdominal pain** *or* **diarrhoea** *or* **nausea/vomiting** | 71 | 73 | 0.719 | 5.5 | 0.8 |
| **7 symptoms: highest sensitivity** | *loss of taste or loss of smell or fever or cough or* **fatigue/weakness** *or* **headache** *or* **sore throat** | 94 | 25 | 0.598 | 10.3 | 1.9 |
| **7 symptoms: highest AUROC** | *loss of taste or loss of smell or fever or* **fatigue/weakness** *or* **muscle ache/myalgia** *or* **abdominal pain** *or* **nausea/vomiting** | 82 | 62 | 0.722 | 6.4 | 1.1 |
| **7 symptoms: lowest TPC** | *loss of taste or loss of smell or fever or* **muscle ache/myalgia** *or* **shortness of breath** *or* **abdominal pain** *or* **nausea/vomiting** | 78 | 66 | 0.72 | 6.1 | 0.9 |
| **7 symptoms: lowest inflation factor** | *loss of taste or loss of smell or fever or* **shortness of breath** *or* **abdominal pain** *or* **diarrhoea** *or* **nausea/vomiting** | 73 | 68 | 0.704 | 6.2 | 0.9 |
| ***not yet vaccinated*** | | | | | | |
| **Any of the 12 elicited symptoms** | Any one of the 12 elicited symptoms | 89 | 34 | 0.613 | 7.6 | 2.2 |
| **4 classic symptoms** | *Fever or cough or loss of taste or loss of smell* | 72 | 74 | 0.731 | 4.2 | 1 |
| **4 classic symptoms + fatigue/weakness**** | *Fever or cough or loss of taste or loss of smell or* **fatigue/weakness** | 80 | 66 | 0.731 | 4.7 | 1.3 |
| **4 classic symptoms + fatigue/weakness + muscle ache/mylagia**** | *Fever or cough or loss of taste or loss of smell or* **fatigue/weakness** *or* **muscle ache/myalgia** | 82 | 63 | 0.725 | 5 | 1.3 |
| **4 symptoms: highest sensitivity** | *loss of smell or cough or* **fatigue/weakness** *or* **headache** | 82 | 55 | 0.685 | 5.8 | 1.6 |
| **4 symptoms: highest AUROC** | *loss of taste or loss of smell or fever or* **fatigue/weakness** | 70 | 83 | 0.764 | 3.2 | 0.7 |
| **4 symptoms: lowest TPC** | *loss of taste or loss of smell or fever or* **shortness of breath** | 60 | 89 | 0.744 | 2.6 | 0.5 |
| **4 symptoms: lowest inflation factor** | *loss of taste or loss of smell or* **abdominal pain** *or* **diarrhoea** | 47 | 89 | 0.679 | 3.1 | 0.5 |
| **1 symptom: highest sensitivity** | **fatigue/weakness** | 53 | 88 | 0.703 | 3.1 | 0.5 |
| **1 symptom: highest AUROC** | **fatigue/weakness** | 53 | 88 | 0.703 | 3.1 | 0.5 |
| **1 symptom: lowest TPC** | *loss of taste* | 32 | 98 | 0.654 | 1.4 | 0.2 |
| **1 symptom: lowest inflation factor** | *loss of smell* | 31 | 98 | 0.648 | 1.4 | 0.1 |
| **2 symptoms: highest sensitivity** | *cough or* **headache** | 70 | 61 | 0.656 | 5.9 | 1.4 |
| **2 symptoms: highest AUROC** | *loss of smell or* **fatigue/weakness** | 63 | 87 | 0.746 | 2.9 | 0.6 |
| **2 symptoms: lowest TPC** | *loss of taste or loss of smell* | 38 | 98 | 0.679 | 1.6 | 0.2 |
| **2 symptoms: lowest inflation factor** | *loss of taste or loss of smell* | 38 | 98 | 0.679 | 1.6 | 0.2 |
| **3 symptoms: highest sensitivity** | *cough or* **fatigue/weakness** *or* **headache** | 78 | 56 | 0.667 | 6 | 1.6 |
| **3 symptoms: highest AUROC** | *loss of smell or fever or* **fatigue/weakness** | 68 | 83 | 0.758 | 3.2 | 0.7 |
| **3 symptoms: lowest TPC** | *loss of taste or loss of smell or fever* | 54 | 93 | 0.734 | 2.2 | 0.4 |
| **3 symptoms: lowest inflation factor** | *loss of taste or loss of smell or* **diarrhoea** | 43 | 93 | 0.681 | 2.5 | 0.4 |
| **5 symptoms: highest sensitivity** | *loss of smell or cough or* **fatigue/weakness** *or* **headache** *or* **sore throat** | 85 | 44 | 0.642 | 6.9 | 1.9 |
| **5 symptoms: highest AUROC** | *loss of taste or loss of smell or fever or* **fatigue/weakness** *or* **muscle ache/myalgia** | 73 | 79 | 0.761 | 3.5 | 0.8 |
| **5 symptoms: lowest TPC** | *loss of taste or loss of smell or fever or* **shortness of breath** *or* **nausea/vomiting** | 62 | 85 | 0.737 | 3.1 | 0.6 |
| **5 symptoms: lowest inflation factor** | *loss of taste or loss of smell or* **abdominal pain** *or* **diarrhoea** *or* **nausea/vomiting** | 50 | 86 | 0.682 | 3.4 | 0.6 |
| **6 symptoms: highest sensitivity** | *loss of smell or fever or cough or* **fatigue/weakness** *or* **headache** *or* **sore throat** | 86 | 42 | 0.642 | 6.9 | 2 |
| **6 symptoms: highest AUROC** | *loss of taste or loss of smell or fever or* **fatigue/weakness** *or* **muscle ache/myalgia** *or* **shortness of breath** | 74 | 77 | 0.757 | 3.7 | 0.9 |
| **6 symptoms: lowest TPC** | *loss of taste or loss of smell or fever or* **shortness of breath** *or* **diarrhoea** *or* **nausea/vomiting** | 64 | 82 | 0.728 | 3.5 | 0.7 |
| **6 symptoms: lowest inflation factor** | *loss of taste or loss of smell or* **shortness of breath** *or* **abdominal pain** *or* **diarrhoea** *or* **nausea/vomiting** | 56 | 83 | 0.696 | 3.7 | 0.7 |
| **7 symptoms: highest sensitivity** | *loss of taste or loss of smell or fever or cough or* **fatigue/weakness** *or* **headache** *or* **sore throat** | 87 | 42 | 0.644 | 6.9 | 2 |
| **7 symptoms: highest AUROC** | *loss of taste or loss of smell or fever or* **fatigue/weakness** *or* **muscle ache/myalgia** *or* **shortness of breath** *or* **nausea/vomiting** | 75 | 75 | 0.747 | 4 | 1 |
| **7 symptoms: lowest TPC** | *loss of taste or loss of smell or fever or* **shortness of breath** *or* **abdominal pain** *or* **diarrhoea** *or* **nausea/vomiting** | 65 | 79 | 0.72 | 3.8 | 0.8 |
| **7 symptoms: lowest inflation factor** | *loss of taste or loss of smell or fever or* **shortness of breath** *or* **abdominal pain** *or* **diarrhoea** *or* **nausea/vomiting** | 65 | 79 | 0.72 | 3.8 | 0.8 |
| ***≥21 days from 1st vaccine, <0 days from 2nd vaccine*** | | | | | | |
| **Any of the 12 elicited symptoms** | Any one of the 12 elicited symptoms | 96 | 15 | 0.555 | 35.2 | 3 |
| **4 classic symptoms** | *Fever or cough or loss of taste or loss of smell* | 78 | 73 | 0.757 | 14.2 | 1 |
| **4 classic symptoms + fatigue/weakness**** | *Fever or cough or loss of taste or loss of smell or* **fatigue/weakness** | 88 | 60 | 0.741 | 18.5 | 1.5 |
| **4 classic symptoms + fatigue/weakness + muscle ache/mylagia**** | *Fever or cough or loss of taste or loss of smell or* **fatigue/weakness** *or* **muscle ache/myalgia** | 90 | 54 | 0.72 | 20.7 | 1.7 |
| **4 symptoms: highest sensitivity** | *loss of smell or cough or* **fatigue/weakness** *or* **headache** | 90 | 42 | 0.662 | 25.7 | 2.1 |
| **4 symptoms: highest AUROC** | *loss of taste or loss of smell or fever or cough* | 78 | 73 | 0.757 | 14.2 | 1 |
| **4 symptoms: lowest TPC** | *loss of taste or loss of smell or fever or* **nausea/vomiting** | 53 | 86 | 0.698 | 11 | 0.5 |
| **4 symptoms: lowest inflation factor** | *loss of taste or loss of smell or fever or* **nausea/vomiting** | 53 | 86 | 0.698 | 11 | 0.5 |
| **1 symptom: highest sensitivity** | *cough* | 63 | 79 | 0.71 | 13.7 | 0.8 |
| **1 symptom: highest AUROC** | *cough* | 63 | 79 | 0.71 | 13.7 | 0.8 |
| **1 symptom: lowest TPC** | *loss of taste* | 38 | 98 | 0.68 | 3.3 | 0.1 |
| **1 symptom: lowest inflation factor** | *loss of taste* | 38 | 98 | 0.68 | 3.3 | 0.1 |
| **2 symptoms: highest sensitivity** | *cough or* **fatigue/weakness** | 80 | 64 | 0.718 | 18.4 | 1.3 |
| **2 symptoms: highest AUROC** | *loss of taste or cough* | 72 | 78 | 0.75 | 12.9 | 0.8 |
| **2 symptoms: lowest TPC** | *loss of taste or loss of smell* | 44 | 96 | 0.701 | 4.2 | 0.2 |
| **2 symptoms: lowest inflation factor** | *loss of taste or loss of smell* | 44 | 96 | 0.701 | 4.2 | 0.2 |
| **3 symptoms: highest sensitivity** | *cough or* **fatigue/weakness** *or* **headache** | 87 | 44 | 0.651 | 26.1 | 2 |
| **3 symptoms: highest AUROC** | *loss of taste or loss of smell or cough* | 75 | 77 | 0.757 | 13 | 0.9 |
| **3 symptoms: lowest TPC** | *loss of taste or loss of smell or fever* | 52 | 92 | 0.717 | 7.3 | 0.3 |
| **3 symptoms: lowest inflation factor** | *loss of taste or loss of smell or fever* | 52 | 92 | 0.717 | 7.3 | 0.3 |
| **5 symptoms: highest sensitivity** | *loss of smell or cough or* **fatigue/weakness** *or* **headache** *or* **sore throat** | 92 | 32 | 0.622 | 29.4 | 2.4 |
| **5 symptoms: highest AUROC** | *loss of taste or loss of smell or fever or cough or* **fatigue/weakness** | 88 | 60 | 0.741 | 18.5 | 1.5 |
| **5 symptoms: lowest TPC** | *loss of taste or loss of smell or fever or* **abdominal pain** *or* **nausea/vomiting** | 55 | 81 | 0.679 | 14.3 | 0.7 |
| **5 symptoms: lowest inflation factor** | *loss of taste or loss of smell or fever or* **abdominal pain** *or* **nausea/vomiting** | 55 | 81 | 0.679 | 14.3 | 0.7 |
| **6 symptoms: highest sensitivity** | *loss of smell or cough or* **fatigue/weakness** *or* **headache** *or* **sore throat** *or* **diarrhoea** | 94 | 27 | 0.605 | 30.9 | 2.6 |
| **6 symptoms: highest AUROC** | *loss of taste or loss of smell or fever or cough or* **fatigue/weakness** *or* **shortness of breath** | 90 | 56 | 0.73 | 19.8 | 1.6 |
| **6 symptoms: lowest TPC** | *loss of taste or loss of smell or fever or* **abdominal pain** *or* **diarrhoea** *or* **nausea/vomiting** | 58 | 76 | 0.671 | 16.9 | 0.9 |
| **6 symptoms: lowest inflation factor** | *loss of taste or loss of smell or fever or* **abdominal pain** *or* **diarrhoea** *or* **nausea/vomiting** | 58 | 76 | 0.671 | 16.9 | 0.9 |
| **7 symptoms: highest sensitivity** | *loss of smell or cough or* **fatigue/weakness** *or* **muscle ache/myalgia** *or* **headache** *or* **sore throat** *or* **diarrhoea** | 95 | 23 | 0.59 | 32.3 | 2.8 |
| **7 symptoms: highest AUROC** | *loss of taste or loss of smell or fever or cough or* **fatigue/weakness** *or* **shortness of breath** *or* **abdominal pain** | 90 | 52 | 0.714 | 21.3 | 1.7 |
| **7 symptoms: lowest TPC** | *loss of taste or loss of smell or fever or* **fatigue/weakness** *or* **abdominal pain** *or* **diarrhoea** *or* **nausea/vomiting** | 77 | 64 | 0.705 | 19.1 | 1.3 |
| **7 symptoms: lowest inflation factor** | *loss of taste or loss of smell or fever or* **shortness of breath** *or* **abdominal pain** *or* **diarrhoea** *or* **nausea/vomiting** | 63 | 70 | 0.662 | 19.6 | 1.1 |
| ***≥14 days from 2nd vaccine*** | | | | | | |
| **Any of the 12 elicited symptoms** | Any one of the 12 elicited symptoms | 95 | 12 | 0.537 | 16.3 | 2.6 |
| **4 classic symptoms** | *Fever or cough or loss of taste or loss of smell* | 82 | 69 | 0.753 | 7.3 | 1 |
| **4 classic symptoms + fatigue/weakness**** | *Fever or cough or loss of taste or loss of smell or* **fatigue/weakness** | 88 | 56 | 0.722 | 9.3 | 1.4 |
| **4 classic symptoms + fatigue/weakness + muscle ache/mylagia**** | *Fever or cough or loss of taste or loss of smell or* **fatigue/weakness** *or* **muscle ache/myalgia** | 90 | 50 | 0.702 | 10.2 | 1.5 |
| **4 symptoms: highest sensitivity** | *loss of smell or cough or* **fatigue/weakness** *or* **headache** | 91 | 43 | 0.669 | 11.4 | 1.7 |
| **4 symptoms: highest AUROC** | *loss of taste or loss of smell or fever or* **muscle ache/myalgia** | 73 | 80 | 0.763 | 5.6 | 0.7 |
| **4 symptoms: lowest TPC** | *loss of taste or loss of smell or fever or* **nausea/vomiting** | 66 | 85 | 0.752 | 4.9 | 0.5 |
| **4 symptoms: lowest inflation factor** | *loss of taste or loss of smell or fever or* **nausea/vomiting** | 66 | 85 | 0.752 | 4.9 | 0.5 |
| **1 symptom: highest sensitivity** | *cough* | 64 | 75 | 0.696 | 7.5 | 0.8 |
| **1 symptom: highest AUROC** | **fatigue/weakness** | 60 | 80 | 0.7 | 6.6 | 0.7 |
| **1 symptom: lowest TPC** | *loss of smell* | 41 | 97 | 0.692 | 2.1 | 0.1 |
| **1 symptom: lowest inflation factor** | *loss of taste* | 40 | 97 | 0.684 | 2.2 | 0.1 |
| **2 symptoms: highest sensitivity** | *cough or* **headache** | 81 | 54 | 0.677 | 10.3 | 1.4 |
| **2 symptoms: highest AUROC** | *loss of smell or fever* | 60 | 92 | 0.759 | 3.2 | 0.3 |
| **2 symptoms: lowest TPC** | *loss of taste or loss of smell* | 47 | 96 | 0.714 | 2.5 | 0.2 |
| **2 symptoms: lowest inflation factor** | *loss of taste or loss of smell* | 47 | 96 | 0.714 | 2.5 | 0.2 |
| **3 symptoms: highest sensitivity** | *cough or* **fatigue/weakness** *or* **headache** | 87 | 44 | 0.657 | 11.6 | 1.7 |
| **3 symptoms: highest AUROC** | *loss of taste or loss of smell or fever* | 63 | 91 | 0.769 | 3.5 | 0.4 |
| **3 symptoms: lowest TPC** | *loss of taste or loss of smell or fever* | 63 | 91 | 0.769 | 3.5 | 0.4 |
| **3 symptoms: lowest inflation factor** | *loss of taste or loss of smell or fever* | 63 | 91 | 0.769 | 3.5 | 0.4 |
| **5 symptoms: highest sensitivity** | *loss of smell or cough or* **fatigue/weakness** *or* **headache** *or* **sore throat** | 92 | 32 | 0.62 | 13.3 | 2 |
| **5 symptoms: highest AUROC** | *loss of taste or loss of smell or fever or* **muscle ache/myalgia** *or* **nausea/vomiting** | 74 | 75 | 0.745 | 6.6 | 0.8 |
| **5 symptoms: lowest TPC** | *loss of taste or loss of smell or fever or* **abdominal pain** *or* **nausea/vomiting** | 67 | 80 | 0.734 | 6 | 0.7 |
| **5 symptoms: lowest inflation factor** | *loss of taste or loss of smell or fever or* **abdominal pain** *or* **nausea/vomiting** | 67 | 80 | 0.734 | 6 | 0.7 |
| **6 symptoms: highest sensitivity** | *loss of smell or fever or cough or* **fatigue/weakness** *or* **headache** *or* **sore throat** | 93 | 30 | 0.618 | 13.4 | 2.1 |
| **6 symptoms: highest AUROC** | *loss of taste or loss of smell or fever or* **fatigue/weakness** *or* **abdominal pain** *or* **nausea/vomiting** | 79 | 66 | 0.729 | 8 | 1.1 |
| **6 symptoms: lowest TPC** | *loss of taste or loss of smell or fever or* **abdominal pain** *or* **diarrhoea** *or* **nausea/vomiting** | 68 | 74 | 0.707 | 7.4 | 0.8 |
| **6 symptoms: lowest inflation factor** | *loss of taste or loss of smell or fever or* **abdominal pain** *or* **diarrhoea** *or* **nausea/vomiting** | 68 | 74 | 0.707 | 7.4 | 0.8 |
| **7 symptoms: highest sensitivity** | *loss of smell or fever or cough or* **fatigue/weakness** *or* **muscle ache/myalgia** *or* **headache** *or* **sore throat** | 94 | 26 | 0.598 | 14.1 | 2.2 |
| **7 symptoms: highest AUROC** | *loss of taste or loss of smell or fever or* **fatigue/weakness** *or* **muscle ache/myalgia** *or* **abdominal pain** *or* **nausea/vomiting** | 82 | 61 | 0.713 | 9 | 1.2 |
| **7 symptoms: lowest TPC** | *loss of taste or loss of smell or fever or* **muscle ache/myalgia** *or* **abdominal pain** *or* **diarrhoea** *or* **nausea/vomiting** | 75 | 65 | 0.701 | 8.7 | 1.1 |
| **7 symptoms: lowest inflation factor** | *loss of taste or loss of smell or fever or* **shortness of breath** *or* **abdominal pain** *or* **diarrhoea** *or* **nausea/vomiting** | 72 | 66 | 0.69 | 8.8 | 1.1 |
| ***2-5 years*** | | | | | | |
| **Any of the 12 elicited symptoms** | Any one of the 12 elicited symptoms | 83 | 21 | 0.521 | 19.5 | 1.2 |
| **4 classic symptoms** | *Fever or cough or loss of taste or loss of smell* | 68 | 35 | 0.516 | 19.6 | 1 |
| **4 classic symptoms + fatigue/weakness**** | *Fever or cough or loss of taste or loss of smell or* **fatigue/weakness** | 73 | 33 | 0.531 | 18.8 | 1 |
| **4 classic symptoms + fatigue/weakness + muscle ache/mylagia**** | *Fever or cough or loss of taste or loss of smell or* **fatigue/weakness** *or* **muscle ache/myalgia** | 73 | 33 | 0.529 | 18.9 | 1 |
| **4 symptoms: highest sensitivity** | *fever or cough or* **fatigue/weakness** *or* **diarrhoea** | 76 | 31 | 0.536 | 18.6 | 1.1 |
| **4 symptoms: highest AUROC** | *loss of taste or fever or* **fatigue/weakness** *or* **diarrhoea** | 59 | 72 | 0.656 | 10.1 | 0.4 |
| **4 symptoms: lowest TPC** | *loss of taste or loss of smell or* **muscle ache/myalgia** *or* **headache** | 24 | 94 | 0.588 | 6.2 | 0.1 |
| **4 symptoms: lowest inflation factor** | *loss of taste or loss of smell or* **muscle ache/myalgia** *or* **shortness of breath** | 14 | 96 | 0.553 | 6.3 | 0.1 |
| **1 symptom: highest sensitivity** | *fever* | 42 | 80 | 0.606 | 10.6 | 0.3 |
| **1 symptom: highest AUROC** | *fever* | 42 | 80 | 0.606 | 10.6 | 0.3 |
| **1 symptom: lowest TPC** | *loss of taste* | 8 | 99 | 0.539 | 2.4 | 0 |
| **1 symptom: lowest inflation factor** | *loss of smell* | 6 | 99 | 0.524 | 3.4 | 0 |
| **2 symptoms: highest sensitivity** | *fever or cough* | 66 | 36 | 0.51 | 19.9 | 1 |
| **2 symptoms: highest AUROC** | *fever or* **fatigue/weakness** | 53 | 76 | 0.646 | 9.8 | 0.4 |
| **2 symptoms: lowest TPC** | *loss of taste or* **muscle ache/myalgia** | 14 | 98 | 0.561 | 3.5 | 0 |
| **2 symptoms: lowest inflation factor** | *loss of taste or loss of smell* | 8 | 99 | 0.536 | 3.7 | 0 |
| **3 symptoms: highest sensitivity** | *fever or cough or* **fatigue/weakness** | 72 | 34 | 0.531 | 18.8 | 1 |
| **3 symptoms: highest AUROC** | *loss of taste or fever or* **fatigue/weakness** | 55 | 76 | 0.654 | 9.6 | 0.4 |
| **3 symptoms: lowest TPC** | *loss of taste or loss of smell or* **muscle ache/myalgia** | 14 | 98 | 0.558 | 4.2 | 0 |
| **3 symptoms: lowest inflation factor** | *loss of taste or loss of smell or* **muscle ache/myalgia** | 14 | 98 | 0.558 | 4.2 | 0 |
| **5 symptoms: highest sensitivity** | *fever or cough or* **fatigue/weakness** *or* **headache** *or* **diarrhoea** | 79 | 30 | 0.543 | 18.4 | 1.1 |
| **5 symptoms: highest sensitivity** | *fever or cough or* **fatigue/weakness** *or* **sore throat** *or* **diarrhoea** | 79 | 26 | 0.526 | 19.2 | 1.1 |
| **5 symptoms: highest sensitivity** | *fever or cough or* **headache** *or* **sore throat** *or* **diarrhoea** | 79 | 26 | 0.526 | 19.2 | 1.1 |
| **5 symptoms: highest AUROC** | *loss of taste or fever or* **fatigue/weakness** *or* **headache** *or* **diarrhoea** | 62 | 70 | 0.659 | 10.4 | 0.5 |
| **5 symptoms: lowest TPC** | *loss of taste or loss of smell or* **fatigue/weakness** *or* **muscle ache/myalgia** *or* **headache** | 38 | 88 | 0.632 | 7 | 0.2 |
| **5 symptoms: lowest inflation factor** | *loss of taste or loss of smell or* **muscle ache/myalgia** *or* **headache** *or* **shortness of breath** | 24 | 92 | 0.583 | 7.2 | 0.1 |
| **6 symptoms: highest sensitivity** | *fever or cough or* **fatigue/weakness** *or* **headache** *or* **sore throat** *or* **diarrhoea** | 82 | 25 | 0.534 | 18.9 | 1.2 |
| **6 symptoms: highest AUROC** | *loss of taste or fever or* **fatigue/weakness** *or* **headache** *or* **shortness of breath** *or* **diarrhoea** | 62 | 69 | 0.658 | 10.6 | 0.5 |
| **6 symptoms: lowest TPC** | *loss of taste or loss of smell or* **fatigue/weakness** *or* **muscle ache/myalgia** *or* **headache** *or* **shortness of breath** | 39 | 87 | 0.629 | 7.5 | 0.2 |
| **6 symptoms: lowest inflation factor** | *loss of taste or loss of smell or* **muscle ache/myalgia** *or headache or* **shortness of breath** *or* **abdominal pain** | 31 | 88 | 0.593 | 8.6 | 0.2 |
| **7 symptoms: highest sensitivity** | *loss of taste or fever or cough or* **fatigue/weakness** *or* **headache** *or* **sore throat** *or* **diarrhoea** | 82 | 25 | 0.537 | 18.7 | 1.2 |
| **7 symptoms: highest sensitivity** | *loss of smell or fever or cough or* **fatigue/weakness** *or headache or* **sore throat** *or* **diarrhoea** | 82 | 25 | 0.535 | 18.8 | 1.2 |
| **7 symptoms: highest sensitivity** | *fever or cough or* **fatigue/weakness** *or* **headache** *or* **sore throat** *or* **abdominal pain** *or* **diarrhoea** | 82 | 23 | 0.528 | 19.1 | 1.2 |
| **7 symptoms: highest sensitivity** | *fever or cough or* **fatigue/weakness** *or* **headache** *or* **sore throat** *or* **diarrhoea** *or* **nausea/vomiting** | 82 | 23 | 0.527 | 19.2 | 1.2 |
| **7 symptoms: highest AUROC** | *loss of taste or fever or* **fatigue/weakness** *or* **muscle ache/myalgia** *or* **headache** *or* **shortness of breath** *or* **diarrhoea** | 62 | 69 | 0.656 | 10.7 | 0.5 |
| **7 symptoms: lowest TPC** | *loss of taste or loss of smell or* **fatigue/weakness** *or* **muscle ache/myalgia** *or* **headache** *or* **shortness of breath** *or* **abdominal pain** | 43 | 84 | 0.633 | 8.3 | 0.3 |
| **7 symptoms: lowest inflation factor** | *loss of taste or loss of smell or* **muscle ache/myalgia** *or* **headache** *or* **shortness of breath** *or* **abdominal pain** *or* **diarrhoea** | 36 | 85 | 0.604 | 9.3 | 0.3 |
| ***6-10 years*** | | | | | | |
| **Any of the 12 elicited symptoms** | Any one of the 12 elicited symptoms | 82 | 22 | 0.523 | 12.7 | 1.8 |
| **4 classic symptoms** | *Fever or cough or loss of taste or loss of smell* | 60 | 59 | 0.593 | 9.5 | 1 |
| **4 classic symptoms + fatigue/weakness**** | *Fever or cough or loss of taste or loss of smell or* **fatigue/weakness** | 67 | 55 | 0.61 | 9.3 | 1.1 |
| **4 classic symptoms + fatigue/weakness + muscle ache/mylagia**** | *Fever or cough or loss of taste or loss of smell or* **fatigue/weakness** *or* **muscle ache/myalgia** | 68 | 54 | 0.606 | 9.5 | 1.1 |
| **4 symptoms: highest sensitivity** | *fever or cough or* **fatigue/weakness** *or* **headache** | 74 | 46 | 0.603 | 9.9 | 1.3 |
| **4 symptoms: highest AUROC** | *loss of smell or fever or* **fatigue/weakness** *or* **headache** | 65 | 71 | 0.679 | 6.5 | 0.7 |
| **4 symptoms: lowest TPC** | *loss of taste or loss of smell or* **muscle ache/myalgia** *or* **shortness of breath** | 25 | 94 | 0.592 | 4.2 | 0.2 |
| **4 symptoms: lowest inflation factor** | *loss of taste or loss of smell or* **muscle ache/myalgia** *or* **shortness of breath** | 25 | 94 | 0.592 | 4.2 | 0.2 |
| **1 symptom: highest sensitivity** | **headache** | 42 | 83 | 0.625 | 5.9 | 0.4 |
| **1 symptom: highest AUROC** | **headache** | 42 | 83 | 0.625 | 5.9 | 0.4 |
| **1 symptom: lowest TPC** | *loss of taste* | 10 | 99 | 0.547 | 2.1 | 0 |
| **1 symptom: lowest inflation factor** | *loss of taste* | 10 | 99 | 0.547 | 2.1 | 0 |
| **2 symptoms: highest sensitivity** | *cough or* **headache** | 62 | 54 | 0.58 | 10.2 | 1.1 |
| **2 symptoms: highest AUROC** | *fever or* **headache** | 55 | 75 | 0.652 | 6.5 | 0.6 |
| **2 symptoms: lowest TPC** | *loss of taste or loss of smell* | 14 | 98 | 0.56 | 2.6 | 0.1 |
| **2 symptoms: lowest inflation factor** | *loss of taste or loss of smell* | 14 | 98 | 0.56 | 2.6 | 0.1 |
| **3 symptoms: highest sensitivity** | *fever or cough or* **headache** | 71 | 49 | 0.599 | 9.9 | 1.2 |
| **3 symptoms: highest AUROC** | *loss of smell or fever or* **headache** | 60 | 75 | 0.671 | 6.3 | 0.7 |
| **3 symptoms: lowest TPC** | *loss of taste or loss of smell or* **shortness of breath** | 16 | 97 | 0.566 | 3.5 | 0.1 |
| **3 symptoms: lowest inflation factor** | *loss of taste or loss of smell or* **shortness of breath** | 16 | 97 | 0.566 | 3.5 | 0.1 |
| **5 symptoms: highest sensitivity** | *fever or cough or* **fatigue/weakness** *or* **headache** *or* **sore throat** | 77 | 33 | 0.552 | 11.7 | 1.6 |
| **5 symptoms: highest AUROC** | *loss of taste or loss of smell or fever or* **fatigue/weakness** *or* **headache** | 65 | 71 | 0.679 | 6.5 | 0.7 |
| **5 symptoms: lowest TPC** | *loss of taste or loss of smell or* **fatigue/weakness** *or* **muscle ache/myalgia** *or* **shortness of breath** | 39 | 88 | 0.635 | 4.9 | 0.3 |
| **5 symptoms: lowest inflation factor** | *loss of taste or loss of smell or* **muscle ache/myalgia** *or* **shortness of breath** *or* **diarrhoea** | 29 | 89 | 0.59 | 5.7 | 0.3 |
| **6 symptoms: highest sensitivity** | *loss of smell or fever or cough or* **fatigue/weakness** *or* **headache** *or* **sore throat** | 79 | 33 | 0.561 | 11.4 | 1.6 |
| **6 symptoms: highest AUROC** | *loss of taste or loss of smell or fever or* **fatigue/weakness** *or* **muscle ache/myalgia** *or* **headache** | 66 | 69 | 0.676 | 6.7 | 0.8 |
| **6 symptoms: lowest TPC** | *loss of taste or loss of smell or fever or* **fatigue/weakness** *or* **muscle ache/myalgia** *or* **shortness of breath** | 53 | 80 | 0.663 | 5.7 | 0.5 |
| **6 symptoms: lowest inflation factor** | *loss of taste or loss of smell or* **muscle ache/myalgia** *or* **shortness of breath** *or* **diarrhoea** *or* **nausea/vomiting** | 35 | 83 | 0.59 | 6.9 | 0.4 |
| **7 symptoms: highest sensitivity** | *loss of smell or fever or cough or* **fatigue/weakness** *or* **headache** *or* **sore throat** *or* **abdominal pain** | 80 | 29 | 0.544 | 12 | 1.7 |
| **7 symptoms: highest AUROC** | *loss of taste or loss of smell or fever or* **fatigue/weakness** *or* **muscle ache/myalgia** *or* **headache** *or* **shortness of breath** | 66 | 69 | 0.673 | 6.8 | 0.8 |
| **7 symptoms: lowest TPC** | *loss of taste or loss of smell or fever or* **fatigue/weakness** *or* **muscle ache/myalgia** *or* **shortness of breath** *or* **diarrhoea** | 54 | 76 | 0.651 | 6.4 | 0.6 |
| **7 symptoms: lowest inflation factor** | *loss of taste or loss of smell or* **fatigue/weakness** *or* **muscle ache/myalgia** *or* **shortness of breath** *or* **diarrhoea** *or* **nausea/vomiting** | 45 | 79 | 0.619 | 6.8 | 0.5 |
| ***11-15*** | | | | | | |
| **Any of the 12 elicited symptoms** | Any one of the 12 elicited symptoms | 89 | 22 | 0.556 | 6.8 | 2.1 |
| **4 classic symptoms** | *Fever or cough or loss of taste or loss of smell* | 68 | 67 | 0.678 | 4.2 | 1 |
| **4 classic symptoms + fatigue/weakness**** | *Fever or cough or loss of taste or loss of smell or* **fatigue/weakness** | 76 | 61 | 0.685 | 4.4 | 1.2 |
| **4 classic symptoms + fatigue/weakness + muscle ache/mylagia**** | *Fever or cough or loss of taste or loss of smell or* **fatigue/weakness** *or* **muscle ache/myalgia** | 77 | 59 | 0.681 | 4.5 | 1.2 |
| **4 symptoms: highest sensitivity** | *loss of smell or cough or* **headache** *or* **sore throat** | 81 | 36 | 0.585 | 6.2 | 1.8 |
| **4 symptoms: highest AUROC** | *loss of taste or loss of smell or fever or* **fatigue/weakness** | 66 | 82 | 0.738 | 2.9 | 0.7 |
| **4 symptoms: lowest TPC** | *loss of taste or loss of smell or* **muscle ache/myalgia** *or* **shortness of breath** | 49 | 90 | 0.698 | 2.3 | 0.4 |
| **4 symptoms: lowest inflation factor** | *loss of taste or loss of smell or* **shortness of breath** *or* **diarrhoea** | 41 | 91 | 0.661 | 2.4 | 0.3 |
| **1 symptom: highest sensitivity** | **headache** | 50 | 73 | 0.617 | 4.6 | 0.8 |
| **1 symptom: highest AUROC** | **fatigue/weakness** | 40 | 88 | 0.642 | 2.9 | 0.4 |
| **1 symptom: lowest TPC** | *loss of taste* | 24 | 98 | 0.608 | 1.5 | 0.1 |
| **1 symptom: lowest inflation factor** | **shortness of breath** | 12 | 98 | 0.55 | 2.3 | 0.1 |
| **2 symptoms: highest sensitivity** | *cough or* **headache** | 66 | 52 | 0.59 | 5.8 | 1.4 |
| **2 symptoms: highest AUROC** | *loss of smell or* **fatigue/weakness** | 54 | 87 | 0.707 | 2.6 | 0.5 |
| **2 symptoms: lowest TPC** | *loss of taste or loss of smell* | 32 | 97 | 0.645 | 1.7 | 0.2 |
| **2 symptoms: lowest inflation factor** | *loss of taste or loss of smell* | 32 | 97 | 0.645 | 1.7 | 0.2 |
| **3 symptoms: highest sensitivity** | *loss of smell or cough or* **headache** | 74 | 51 | 0.626 | 5.4 | 1.4 |
| **3 symptoms: highest AUROC** | *loss of smell or fever or* **fatigue/weakness** | 64 | 82 | 0.731 | 2.8 | 0.6 |
| **3 symptoms: lowest TPC** | *loss of taste or loss of smell or* **shortness of breath** | 38 | 95 | 0.666 | 1.9 | 0.3 |
| **3 symptoms: lowest inflation factor** | *loss of taste or loss of smell or* **shortness of breath** | 38 | 95 | 0.666 | 1.9 | 0.3 |
| **5 symptoms: highest sensitivity** | *loss of smell or fever or cough or* **headache** *or* **sore throat** | 84 | 34 | 0.591 | 6.2 | 1.8 |
| **5 symptoms: highest AUROC** | *loss of taste or loss of smell or fever or* **fatigue/weakness** *or* **shortness of breath** | 68 | 80 | 0.74 | 2.9 | 0.7 |
| **5 symptoms: lowest TPC** | *loss of taste or loss of smell or fever or* **muscle ache/myalgia** *or* **shortness of breath** | 61 | 84 | 0.729 | 2.7 | 0.6 |
| **5 symptoms: lowest inflation factor** | *loss of taste or loss of smell or* **muscle ache/myalgia** *or* **shortness of breath** *or* **diarrhoea** | 51 | 87 | 0.689 | 2.7 | 0.5 |
| **6 symptoms: highest sensitivity** | *loss of smell or fever or cough or* **fatigue/weakness** *or* **headache** *or* **sore throat** | 87 | 31 | 0.588 | 6.3 | 1.9 |
| **6 symptoms: highest AUROC** | *loss of taste or loss of smell or fever or* **fatigue/weakness** *or* **muscle ache/myalgia** *or* **shortness of breath** | 69 | 78 | 0.737 | 3.1 | 0.8 |
| **6 symptoms: lowest TPC** | *loss of taste or loss of smell or fever or* **muscle ache/myalgia** *or* **shortness of breath** *or* **diarrhoea** | 62 | 82 | 0.718 | 3 | 0.6 |
| **6 symptoms: lowest inflation factor** | *loss of taste or loss of smell or* **muscle ache/myalgia** *or* **shortness of breath** *or* **abdominal pain** *or* **diarrhoea** | 53 | 82 | 0.674 | 3.3 | 0.6 |
| **7 symptoms: highest sensitivity** | *loss of taste or loss of smell or fever or cough or* **fatigue/weakness** *or* **headache** *or* **sore throat** | 88 | 31 | 0.591 | 6.3 | 1.9 |
| **7 symptoms: highest AUROC** | *loss of taste or loss of smell or fever or* **fatigue/weakness** *or* **muscle ache/myalgia** *or* **shortness of breath** *or* **diarrhoea** | 70 | 76 | 0.726 | 3.3 | 0.8 |
| **7 symptoms: lowest TPC** | *loss of taste or loss of smell or fever or* **fatigue/weakness** *or* **muscle ache/myalgia** *or* **shortness of breath** *or* **diarrhoea** | 70 | 76 | 0.726 | 3.3 | 0.8 |
| **7 symptoms: lowest inflation factor** | *loss of taste or loss of smell or* **muscle ache/myalgia** *or* **shortness of breath** *or* **abdominal pain** *or* **diarrhoea** *or* **nausea/vomiting** | 56 | 78 | 0.667 | 3.7 | 0.7 |
| ***16-44*** | | | | | | |
| **Any of the 12 elicited symptoms** | Any one of the 12 elicited symptoms | 90 | 27 | 0.586 | 7.2 | 2.3 |
| **4 classic symptoms** | *Fever or cough or loss of taste or loss of smell* | 76 | 74 | 0.746 | 3.7 | 1 |
| **4 classic symptoms + fatigue/weakness**** | *Fever or cough or loss of taste or loss of smell or* **fatigue/weakness** | 83 | 64 | 0.735 | 4.3 | 1.3 |
| **4 classic symptoms + fatigue/weakness + muscle ache/mylagia**** | *Fever or cough or loss of taste or loss of smell or* **fatigue/weakness** *or* **muscle ache/myalgia** | 85 | 61 | 0.728 | 4.5 | 1.4 |
| **4 symptoms: highest sensitivity** | *loss of smell or cough or* **fatigue/weakness** *or* **headache** | 85 | 49 | 0.669 | 5.6 | 1.7 |
| **4 symptoms: highest AUROC** | *loss of taste or loss of smell or fever or* **muscle ache/myalgia** | 69 | 86 | 0.776 | 2.5 | 0.6 |
| **4 symptoms: lowest TPC** | *loss of taste or loss of smell or fever or* **shortness of breath** | 65 | 89 | 0.769 | 2.3 | 0.5 |
| **4 symptoms: lowest inflation factor** | *loss of taste or loss of smell or* **abdominal pain** *or* **diarrhoea** | 52 | 89 | 0.704 | 2.6 | 0.5 |
| **1 symptom: highest sensitivity** | **fatigue/weakness** | 56 | 85 | 0.704 | 3 | 0.6 |
| **1 symptom: highest AUROC** | **fatigue/weakness** | 56 | 85 | 0.704 | 3 | 0.6 |
| **1 symptom: lowest TPC** | *loss of smell* | 39 | 98 | 0.688 | 1.4 | 0.2 |
| **1 symptom: lowest inflation factor** | **diarrhoea** | 11 | 95 | 0.528 | 4.6 | 0.2 |
| **2 symptoms: highest sensitivity** | *cough or* **headache** | 72 | 56 | 0.643 | 5.6 | 1.5 |
| **2 symptoms: highest AUROC** | *loss of smell or* **fatigue/weakness** | 68 | 84 | 0.759 | 2.8 | 0.7 |
| **2 symptoms: lowest TPC** | *loss of taste or loss of smell* | 45 | 97 | 0.713 | 1.5 | 0.2 |
| **2 symptoms: lowest inflation factor** | *loss of taste or loss of smell* | 45 | 97 | 0.713 | 1.5 | 0.2 |
| **3 symptoms: highest sensitivity** | *loss of smell or cough or* **headache** | 80 | 55 | 0.679 | 5.3 | 1.5 |
| **3 symptoms: highest AUROC** | *loss of smell or fever or* **fatigue/weakness** | 73 | 81 | 0.77 | 3 | 0.8 |
| **3 symptoms: lowest TPC** | *loss of taste or loss of smell or* **shortness of breath** | 54 | 93 | 0.736 | 1.9 | 0.4 |
| **3 symptoms: lowest inflation factor** | *loss of taste or loss of smell or* **shortness of breath** | 54 | 93 | 0.736 | 1.9 | 0.4 |
| **5 symptoms: highest sensitivity** | *loss of smell or cough or* **fatigue/weakness** *or* **headache** *or* **sore throat** | 87 | 36 | 0.615 | 6.6 | 2.1 |
| **5 symptoms: highest AUROC** | *loss of taste or loss of smell or fever or* **muscle ache/myalgia** *or* **shortness of breath** | 72 | 83 | 0.776 | 2.8 | 0.7 |
| **5 symptoms: lowest TPC** | *loss of taste or loss of smell or fever* **shortness of breath** *or* **diarrhoea** | 66 | 85 | 0.756 | 2.7 | 0.6 |
| **5 symptoms: lowest inflation factor** | *loss of taste or loss of smell or* **shortness of breath** *or* **abdominal pain** *or* **diarrhoea** | 58 | 86 | 0.719 | 2.9 | 0.6 |
| **6 symptoms: highest sensitivity** | *loss of smell or fever or cough or* **fatigue/weakness** *or* **headache** *or* **sore throat** | 88 | 35 | 0.613 | 6.7 | 2.1 |
| **6 symptoms: highest AUROC** | *loss of taste or loss of smell or fever or* **fatigue/weakness** *or* **muscle ache/myalgia** *or* **shortness of breath** | 78 | 75 | 0.768 | 3.4 | 1 |
| **6 symptoms: lowest TPC** | *loss of taste or loss of smell or fever or* **shortness of breath** *or* **abdominal pain** *or* **diarrhoea** | 67 | 82 | 0.745 | 3.1 | 0.7 |
| **6 symptoms: lowest inflation factor** | *loss of taste or loss of smell or* **shortness of breath** *or* **abdominal pain** *or* **diarrhoea** *or* **nausea/vomiting** | 60 | 82 | 0.713 | 3.2 | 0.7 |
| **7 symptoms: highest sensitivity** | *loss of taste or loss of smell or fever or cough or* **fatigue/weakness** *or* **headache** *or* **sore throat** | 89 | 34 | 0.615 | 6.7 | 2.1 |
| **7 symptoms: highest AUROC** | *loss of taste or loss of smell or fever or* **fatigue/weakness** *or* **muscle ache/myalgia** *or* **shortness of breath** *or* **abdominal pain** | 79 | 72 | 0.756 | 3.7 | 1 |
| **7 symptoms: lowest TPC** | *loss of taste or loss of smell or fever or* **shortness of breath** *or* **abdominal pain** *or* **diarrhoea** *or* **nausea/vomiting** | 68 | 79 | 0.736 | 3.4 | 0.8 |
| **7 symptoms: lowest inflation factor** | *loss of taste or loss of smell or fever or* **shortness of breath** *or* **abdominal pain** *or* **diarrhoea** *or* **nausea/vomiting** | 68 | 79 | 0.736 | 3.4 | 0.8 |
| ***45-64*** | | | | | | |
| **Any of the 12 elicited symptoms** | Any one of the 12 elicited symptoms | 91 | 31 | 0.611 | 8.2 | 2.5 |
| **4 classic symptoms** | *Fever or cough or loss of taste or loss of smell* | 76 | 77 | 0.762 | 3.9 | 1 |
| **4 classic symptoms + fatigue/weakness**** | *Fever or cough or loss of taste or loss of smell or* **fatigue/weakness** | 84 | 67 | 0.754 | 4.7 | 1.3 |
| **4 classic symptoms + fatigue/weakness + muscle ache/mylagia**** | *Fever or cough or loss of taste or loss of smell or* **fatigue/weakness** *or* **muscle ache/myalgia** | 85 | 63 | 0.742 | 5.1 | 1.5 |
| **4 symptoms: highest sensitivity** | *loss of smell or cough or* **fatigue/weakness** *or* **headache** | 86 | 53 | 0.694 | 6.2 | 1.8 |
| **4 symptoms: highest AUROC** | *loss of taste or loss of smell or fever or* **fatigue/weakness** | 74 | 81 | 0.773 | 3.4 | 0.8 |
| **4 symptoms: lowest TPC** | *loss of taste or loss of smell or fever or* **nausea/vomiting** | 60 | 89 | 0.744 | 2.8 | 0.6 |
| **4 symptoms: lowest inflation factor** | *loss of taste or loss of smell or* **abdominal pain** *or* **nausea/vomiting** | 52 | 88 | 0.698 | 3.2 | 0.6 |
| **1 symptom: highest sensitivity** | **fatigue/weakness** | 60 | 85 | 0.726 | 3.3 | 0.7 |
| **1 symptom: highest AUROC** | **fatigue/weakness** | 60 | 85 | 0.726 | 3.3 | 0.7 |
| **1 symptom: lowest TPC** | *loss of taste* | 36 | 98 | 0.672 | 1.5 | 0.2 |
| **1 symptom: lowest inflation factor** | *loss of smell* | 33 | 98 | 0.658 | 1.5 | 0.2 |
| **2 symptoms: highest sensitivity** | *cough or* **fatigue/weakness** | 77 | 70 | 0.736 | 4.7 | 1.2 |
| **2 symptoms: highest AUROC** | *loss of taste or* **fatigue/weakness** | 68 | 84 | 0.759 | 3.2 | 0.7 |
| **2 symptoms: lowest TPC** | *loss of taste or loss of smell* | 41 | 97 | 0.692 | 1.7 | 0.2 |
| **2 symptoms: lowest inflation factor** | *loss of taste or loss of smell* | 41 | 97 | 0.692 | 1.7 | 0.2 |
| **3 symptoms: highest sensitivity** | *cough or* **fatigue/weakness** *or* **headache** | 83 | 54 | 0.685 | 6.3 | 1.7 |
| **3 symptoms: highest AUROC** | *loss of taste or fever or* **fatigue/weakness** | 72 | 82 | 0.77 | 3.4 | 0.8 |
| **3 symptoms: lowest TPC** | *loss of taste or loss of smell or fever* | 57 | 93 | 0.749 | 2.2 | 0.4 |
| **3 symptoms: lowest inflation factor** | *loss of taste or loss of smell or* **nausea/vomiting** | 48 | 92 | 0.702 | 2.6 | 0.4 |
| **5 symptoms: highest sensitivity** | *loss of smell or cough or fatigue/weakness or* **headache** *or* **sore throat** | 88 | 42 | 0.651 | 7.2 | 2.1 |
| **5 symptoms: highest AUROC** | *loss of taste or loss of smell or fever or* **fatigue/weakness** *or* **shortness of breath** | 76 | 78 | 0.767 | 3.8 | 1 |
| **5 symptoms: lowest TPC** | *loss of taste or loss of smell or fever or* **shortness of breath** *or* **nausea/vomiting** | 66 | 84 | 0.75 | 3.3 | 0.7 |
| **5 symptoms: lowest inflation factor** | *loss of taste or loss of smell or* **abdominal pain** *or* **diarrhoea** *or* **nausea/vomiting** | 55 | 84 | 0.694 | 3.7 | 0.7 |
| **6 symptoms: highest sensitivity** | *loss of smell or fever or cough or* **fatigue/weakness** *or* **headache** *or* **sore throat** | 89 | 41 | 0.65 | 7.3 | 2.2 |
| **6 symptoms: highest AUROC** | *loss of taste or loss of smell or fever or* **fatigue/weakness** *or* **muscle ache/myalgia** *or* **shortness of breath** | 78 | 74 | 0.759 | 4.2 | 1.1 |
| **6 symptoms: lowest TPC** | *loss of taste or loss of smell or fever or* **shortness of breath** *or* **abdominal pain** *or* **nausea/vomiting** | 67 | 81 | 0.74 | 3.7 | 0.8 |
| **6 symptoms: lowest inflation factor** | *loss of taste or loss of smell or fever or* **abdominal pain** *or* **diarrhoea** *or* **nausea/vomiting** | 64 | 81 | 0.726 | 3.8 | 0.8 |
| **7 symptoms: highest sensitivity** | *loss of smell or fever or cough or* **fatigue/weakness** *or* **headache** *or* **shortness of breath** *or* **sore throat** | 89 | 40 | 0.646 | 7.4 | 2.2 |
| **7 symptoms: highest AUROC** | *loss of taste or loss of smell or fever or* **fatigue/weakness** *or* **muscle ache/myalgia** *or* **shortness of breath** *or* **nausea/vomiting** | 79 | 71 | 0.75 | 4.5 | 1.2 |
| **7 symptoms: lowest TPC** | *loss of taste or loss of smell or fever or* **shortness of breath** *or* **abdominal pain** *or* **diarrhoea** *or* **nausea/vomiting** | 69 | 77 | 0.729 | 4.1 | 1 |
| **7 symptoms: lowest inflation factor** | *loss of taste or loss of smell or fever or* **shortness of breath** *or* **abdominal pain** *or* **diarrhoea** *or* **nausea/vomiting** | 69 | 77 | 0.729 | 4.1 | 1 |
| ***65+*** | | | | | | |
| **Any of the 12 elicited symptoms** | Any one of the 12 elicited symptoms | 88 | 32 | 0.597 | 14.5 | 2.6 |
| **4 classic symptoms** | *Fever or cough or loss of taste or loss of smell* | 69 | 76 | 0.725 | 7.1 | 1 |
| **4 classic symptoms + fatigue/weakness**** | *Fever or cough or loss of taste or loss of smell or* **fatigue/weakness** | 79 | 65 | 0.718 | 8.7 | 1.4 |
| **4 classic symptoms + fatigue/weakness + muscle ache/mylagia**** | *Fever or cough or loss of taste or loss of smell or* **fatigue/weakness** *or* **muscle ache/myalgia** | 80 | 60 | 0.701 | 9.6 | 1.6 |
| **4 symptoms: highest sensitivity** | *loss of smell or cough or* **fatigue/weakness** *or* **headache** | 82 | 56 | 0.689 | 10.3 | 1.7 |
| **4 symptoms: highest AUROC** | *loss of taste or loss of smell or fever or cough* | 69 | 76 | 0.725 | 7.1 | 1 |
| **4 symptoms: lowest TPC** | *loss of taste or loss of smell or fever or* **nausea/vomiting** | 47 | 90 | 0.684 | 4.7 | 0.5 |
| **4 symptoms: lowest inflation factor** | *loss of taste or loss of smell or fever or* **nausea/vomiting** | 47 | 90 | 0.684 | 4.7 | 0.5 |
| **1 symptom: highest sensitivity** | *cough* | 56 | 80 | 0.677 | 7.3 | 0.8 |
| **1 symptom: highest AUROC** | **fatigue/weakness** | 53 | 84 | 0.685 | 6.2 | 0.7 |
| **1 symptom: lowest TPC** | *loss of taste* | 26 | 98 | 0.618 | 2.3 | 0.1 |
| **1 symptom: lowest inflation factor** | *loss of smell* | 21 | 98 | 0.594 | 2.6 | 0.1 |
| **2 symptoms: highest sensitivity** | *cough or fatigue/weakness* | 73 | 68 | 0.706 | 8.5 | 1.3 |
| **2 symptoms: highest AUROC** | *loss of taste or cough* | 64 | 79 | 0.712 | 6.8 | 0.9 |
| **2 symptoms: lowest TPC** | *loss of taste or loss of smell* | 29 | 97 | 0.631 | 2.8 | 0.2 |
| **2 symptoms: lowest inflation factor** | *loss of taste or loss of smell* | 29 | 97 | 0.631 | 2.8 | 0.2 |
| **3 symptoms: highest sensitivity** | *cough or* **fatigue/weakness** *or* **headache** | 79 | 57 | 0.681 | 10.3 | 1.7 |
| **3 symptoms: highest AUROC** | *loss of taste or fever or cough* | 68 | 76 | 0.723 | 7 | 1 |
| **3 symptoms: lowest TPC** | *loss of taste or loss of smell or fever* | 42 | 94 | 0.68 | 3.5 | 0.3 |
| **3 symptoms: lowest inflation factor** | *loss of taste or loss of smell or fever* | 42 | 94 | 0.68 | 3.5 | 0.3 |
| **5 symptoms: highest sensitivity** | *loss of smell or cough or* **fatigue/weakness** *or* **headache** *or* **sore throat** | 84 | 48 | 0.658 | 11.7 | 2 |
| **5 symptoms: highest AUROC** | *loss of taste or loss of smell or fever or cough or* **fatigue/weakness** | 79 | 65 | 0.718 | 8.7 | 1.4 |
| **5 symptoms: lowest TPC** | *loss of taste or loss of smell or fever or* **abdominal pain** *or* **nausea/vomiting** | 49 | 85 | 0.67 | 6.2 | 0.6 |
| **5 symptoms: lowest inflation factor** | *loss of taste or loss of smell or fever or* **abdominal pain** *or* **nausea/vomiting** | 49 | 85 | 0.67 | 6.2 | 0.6 |
| **6 symptoms: highest sensitivity** | *loss of taste or cough or* **fatigue/weakness** *or* **muscle ache/myalgia** *or* **headache** *or* **sore throat** | 85 | 44 | 0.643 | 12.4 | 2.1 |
| **6 symptoms: highest AUROC** | *loss of taste or loss of smell or fever or cough or* **fatigue/weakness** *or* **nausea/vomiting** | 79 | 62 | 0.708 | 9.2 | 1.5 |
| **6 symptoms: lowest TPC** | *loss of taste or loss of smell or fever or* **abdominal pain** *or* **diarrhoea** *or* **nausea/vomiting** | 51 | 80 | 0.658 | 7.6 | 0.8 |
| **6 symptoms: lowest inflation factor** | *loss of taste or loss of smell or fever or* **abdominal pain** *or* **diarrhoea** *or* **nausea/vomiting** | 51 | 80 | 0.658 | 7.6 | 0.8 |
| **7 symptoms: highest sensitivity** | *loss of taste or cough or* **fatigue/weakness** *or* **muscle ache/myalgia** *or* **headache** *or* **sore throat** *or* **diarrhoea** | 86 | 39 | 0.625 | 13.2 | 2.3 |
| **7 symptoms: highest AUROC** | *loss of taste or loss of smell or fever or cough or* **fatigue/weakness** *or* **shortness of breath** *or* **nausea/vomiting** | 81 | 58 | 0.695 | 9.9 | 1.6 |
| **7 symptoms: lowest TPC** | *loss of taste or loss of smell or fever or* **fatigue/weakness** *or* **abdominal pain** *or* **diarrhoea** *or* **nausea/vomiting** | 67 | 70 | 0.685 | 8.7 | 1.2 |
| **7 symptoms: lowest inflation factor** | *loss of taste or loss of smell or fever or* **shortness of breath** *or* **abdominal pain** *or* **diarrhoea** *or* **nausea/vomiting** | 58 | 73 | 0.654 | 9.1 | 1.1 |

Note: question about fatigue/weakness asked as “weakness/tiredness” to participants. Classic symptoms italicised, other symptoms in bold.

*TPC=1/PPV

**See **Table S8**

Table S8 Sensitivity, test per case (TPC), AUROC and inflation factor for various symptom combinations in different groups of positive episodes vs test-negative visits

A

| **Subgroup** | classic (4) | classic or fatigue/weakness (5) | classic or muscle ache/myalgia (5) | classic or headache (5) | classic or diarrhoea (5) | any of the 12 named symptoms (12) |
| --- | --- | --- | --- | --- | --- | --- |
| **All positives** | **74%/4.6/0.734/1** | **81%/5.3/0.727/1.3** | 78%/5.1/0.726/1.2 | **82%/6.3/0.686/1.5** | 75%/5.1/0.717/1.1 | 90%/8.7/0.593/2.3 |
| **Ct<30** | **78%/5.5/0.756/1** | **85%/6.5/0.744/1.3** | 82%/6.2/0.747/1.2 | **86%/7.8/0.704/1.6** | 79%/6.2/0.738/1.1 | 92%/11/0.604/2.4 |
| **Ct≥30** | 60%/18.5/0.667/1 | **71%/20.9/0.675/1.3** | 66%/20.6/0.663/1.2 | 71%/25.7/0.63/1.6 | 62%/21/0.653/1.2 | 83%/34.6/0.559/2.6 |
| **Wild type, Ct<30** | 74%/6.1/0.759/1 | **80%/6.9/0.76/1.2** | 78%/6.5/0.763/1.1 | 80%/7.3/0.75/1.3 | 75%/6.5/0.753/1.1 | 85%/10.3/0.691/1.9 |
| **Alpha variant compatible, Ct<30** | **79%/6.6/0.761/1** | **87%/8.2/0.749/1.4** | 84%/7.7/0.75/1.2 | 87%/10.6/0.684/1.8 | 80%/7.7/0.741/1.2 | 95%/15/0.578/2.7 |
| **Delta variant compatible, Ct<30** | **83%/6.1/0.738/1** | **88%/7.3/0.705/1.3** | 86%/6.9/0.717/1.2 | **90%/8.1/0.679/1.4** | 84%/7/0.705/1.2 | 95%/12.1/0.529/2.3 |
| **Not yet vaccinated** | 72%/4.2/0.731/1 | **80%/4.7/0.731/1.3** | 77%/4.5/0.729/1.2 | **81%/5.7/0.691/1.5** | 74%/4.6/0.719/1.1 | 89%/7.6/0.613/2.2 |
| ***≥*21 days from 1st vaccine, <0 days from 2nd vaccine** | **78%/14.2/0.757/1** | **88%/18.5/0.741/1.5** | 83%/18.1/0.73/1.3 | 85%/24/0.672/1.8 | 81%/17.2/0.735/1.2 | 96%/35.2/0.555/3 |
| ***≥*14 days from 2nd vaccine** | **82%/7.3/0.753/1** | **88%/9.3/0.722/1.4** | 86%/8.7/0.731/1.3 | 88%/10.4/0.692/1.5 | 83%/8.9/0.716/1.2 | 95%/16.3/0.537/2.6 |
| **2-5y** | 68%/19.6/0.516/1 | **73%/18.8/0.531/1** | 68%/19.6/0.516/1 | 72%/18.9/0.529/1 | 72%/19.3/0.523/1 | 83%/19.5/0.521/1.2 |
| **6-10y** | 60%/9.5/0.593/1 | 67%/9.3/0.61/1.1 | 62%/9.6/0.593/1 | **74%/9.7/0.611/1.3** | 62%/10/0.583/1.1 | 82%/12.7/0.523/1.8 |
| **11-15y** | 68%/4.2/0.678/1 | 76%/4.4/0.685/1.2 | **72%/4.3/0.679/1.1** | **80%/5.3/0.639/1.5** | 69%/4.4/0.668/1.1 | 89%/6.8/0.556/2.1 |
| **16-44y** | **76%/3.7/0.746/1** | **83%/4.3/0.735/1.3** | **80%/4/0.743/1.2** | **84%/5.3/0.681/1.6** | 77%/4/0.732/1.1 | 90%/7.2/0.586/2.3 |
| **45-64y** | **76%/3.9/0.762/1** | **84%/4.7/0.754/1.3** | 81%/4.6/0.752/1.2 | 83%/5.9/0.702/1.6 | 77%/4.5/0.744/1.2 | 91%/8.2/0.611/2.5 |
| **65y+** | **69%/7.1/0.725/1** | **79%/8.7/0.718/1.4** | 74%/8.6/0.707/1.3 | 76%/9.5/0.694/1.5 | 71%/8.5/0.703/1.2 | 88%/14.5/0.597/2.6 |
| **26Apr2020-30Aug2020** | 44%/37.9/0.629/1 | **53%/45.2/0.635/1.5** | 48%/43.3/0.628/1.3 | 49%/51.7/0.609/1.5 | 44%/44.4/0.613/1.2 | 66%/70/0.578/2.8 |
| **1Sep2020-16Nov2020** | 68%/4.7/0.724/1 | **74%/5.1/0.731/1.2** | 72%/4.9/0.73/1.1 | **75%/5.4/0.719/1.3** | 69%/4.9/0.72/1.1 | 81%/7.2/0.671/1.8 |
| **17Nov2020-28Feb2021** | **73%/3.2/0.739/1** | **82%/3.7/0.737/1.3** | 78%/3.6/0.731/1.2 | **83%/4.7/0.671/1.7** | 75%/3.6/0.72/1.2 | 92%/6.2/0.577/2.5 |
| **1Mar2021-16May2021** | **76%/16.5/0.733/1** | **83%/21.4/0.705/1.4** | 79%/20.1/0.709/1.3 | 82%/27.1/0.646/1.8 | 77%/19.4/0.71/1.2 | 92%/38.4/0.534/2.8 |
| **17May2021-17Jul2021** | **82%/5/0.733/1** | **88%/6/0.702/1.3** | 85%/5.6/0.712/1.2 | **89%/6.6/0.673/1.4** | 83%/5.8/0.701/1.2 | 94%/9.7/0.526/2.2 |

Note: For any number of symptoms, the combinations achieving the highest sensitivity and highest AUROC are shown in bold, and optimizing both in grey shading and bold, excluding the combination of any of the 12 named symptoms, given they achieve the highest sensitivity by definition

B

| **Subgroup** | **classic or shortness of breath (5)** | **classic or sore throat (5)** | **classic or abdominal pain (5)** | **classic or nausea/vomiting (5)** |
| --- | --- | --- | --- | --- |
| **All positives** | 76%/4.9/0.729/1.1 | 79%/6.1/0.687/1.4 | 75%/5.1/0.718/1.1 | 75%/5/0.722/1.1 |
| **Ct<30** | 80%/5.9/0.749/1.1 | 83%/7.5/0.708/1.4 | 79%/6.1/0.74/1.1 | 80%/6/0.744/1.1 |
| **Ct≥30** | 64%/19.3/0.672/1.1 | 66%/25.5/0.624/1.5 | 62%/21/0.651/1.2 | 62%/20.6/0.655/1.1 |
| **Wild type, Ct<30** | 75%/6.4/0.757/1.1 | 78%/8.5/0.715/1.5 | 75%/6.5/0.754/1.1 | 76%/6.4/0.757/1.1 |
| **Alpha variant compatible, Ct<30** | 81%/7.2/0.754/1.1 | 84%/9.3/0.712/1.5 | 80%/7.7/0.74/1.2 | 81%/7.5/0.747/1.2 |
| **Delta variant compatible, Ct<30** | 84%/6.6/0.719/1.1 | 89%/7.9/0.683/1.4 | 84%/6.8/0.714/1.1 | 85%/6.8/0.716/1.1 |
| **Not yet vaccinated** | 75%/4.4/0.731/1.1 | 77%/5.6/0.684/1.4 | 74%/4.6/0.717/1.1 | 74%/4.5/0.722/1.1 |
| **≥21 days from 1st vaccine, <0 days from 2nd vaccine** | 80%/16.5/0.741/1.2 | 84%/19.5/0.718/1.5 | 79%/16.9/0.733/1.2 | 79%/16.5/0.735/1.2 |
| **≥14 days from 2nd vaccine** | 83%/8.4/0.729/1.2 | 87%/9.7/0.707/1.4 | 83%/8.4/0.73/1.2 | 83%/8.3/0.732/1.1 |
| **2-5y** | 68%/19.6/0.515/1 | 71%/20.2/0.505/1.1 | 68%/20.2/0.505/1 | 70%/19.9/0.51/1.1 |
| **6-10y** | 60%/9.6/0.59/1 | 65%/11.8/0.54/1.4 | 63%/10.4/0.575/1.1 | 63%/10.2/0.58/1.1 |
| **11-15y** | 70%/4.2/0.681/1 | 76%/5.4/0.63/1.4 | 70%/4.6/0.658/1.1 | 71%/4.6/0.663/1.1 |
| **16-44y** | 78%/3.8/0.745/1.1 | 81%/5.2/0.685/1.5 | 77%/4.1/0.73/1.1 | 77%/4.1/0.731/1.1 |
| **45-64y** | 79%/4.2/0.76/1.1 | 81%/5.4/0.714/1.5 | 77%/4.4/0.747/1.1 | 78%/4.3/0.752/1.1 |
| **65y+** | 73%/8.2/0.711/1.2 | 74%/9.2/0.693/1.4 | 71%/8.1/0.707/1.2 | 71%/7.8/0.715/1.1 |
| **26Apr2020-30Aug2020** | 48%/39.8/0.638/1.2 | 53%/52.2/0.615/1.7 | 45%/43.1/0.62/1.2 | 46%/41.4/0.627/1.2 |
| **1Sep2020-16Nov2020** | 70%/4.8/0.725/1.1 | 72%/6.3/0.683/1.4 | 69%/4.9/0.72/1.1 | 70%/4.9/0.723/1.1 |
| **17Nov2020-28Feb2021** | 76%/3.4/0.737/1.1 | 78%/4.2/0.69/1.4 | 74%/3.6/0.718/1.2 | 75%/3.5/0.725/1.1 |
| **1Mar2021-16May2021** | 78%/18.2/0.724/1.1 | 81%/23.1/0.683/1.5 | 78%/19.1/0.714/1.2 | 77%/19/0.714/1.2 |
| **17May2021-17Jul2021** | 83%/5.5/0.716/1.1 | 88%/6.5/0.677/1.4 | 83%/5.6/0.71/1.1 | 84%/5.6/0.711/1.1 |

Table S9 Number of visits and number of positive tests contributing to the analysis of symptoms reported in positive episodes (N=27,869)

|  | **Number within 35 days, including visit of index positive** | | | | | |
| --- | --- | --- | --- | --- | --- | --- |
|  | **1** | **2** | **3** | **4** | **5** | **6+** |
| **Number of visits** | 8124  (29%) | 13929  (50%) | 2672  (10%) | 1856  (7%) | 1192  (4%) | 96  (0.3%) |
| **Number of positives** | 23303  (84%) | 3429  (12%) | 843  (3%) | 224  (0.8%) | 43  (0.2%) |  |

Table S10 Summary of number of symptoms among positive episodes present at index positive and ever present at any future visits within [0,35] days of the index positive, including at visits with negative or missing results (both absent, both present, absent then present, present then absent for each symptom).

| **Symptom**  **n(%)** | **Absent at both**  **index positive and all future visits within 35 days** | **Absent at**  **index positive,**  **present at at least one subsequent visit** | **Present at index positive,**  **absent at all future visits within 35 days** | **Present at both index positive and at least one subsequent visit** |
| --- | --- | --- | --- | --- |
| **All episodes** | | | | |
| any evidence of symptoms | 9476 (49%) | **2880 (15%)** | 4602 (24%) | 2283 (12%) |
| fatigue weakness | 14369 (73%) | **1667 (8%)** | 3027 (15%) | 642 (3%) |
| headache | 14731 (75%) | **1347 (7%)** | 3084 (16%) | 543 (3%) |
| cough | 14570 (74%) | **1262 (6%)** | 3143 (16%) | 730 (4%) |
| loss of taste | 16474 (84%) | **1113 (6%)** | 1802 (9%) | 316 (2%) |
| loss of smell | 16579 (84%) | **1080 (5%)** | 1710 (9%) | 336 (2%) |
| muscle ache myalgia | 15875 (81%) | **1077 (5%)** | 2395 (12%) | 358 (2%) |
| sore throat | 16370 (83%) | **887 (5%)** | 2138 (11%) | 310 (2%) |
| shortness of breath | 17318 (88%) | **887 (5%)** | 1248 (6%) | 252 (1%) |
| fever | 16690 (85%) | **678 (3%)** | 2100 (11%) | 237 (1%) |
| nausea vomiting | 18135 (92%) | **496 (3%)** | 966 (5%) | 108 (1%) |
| diarrhoea | 18464 (94%) | **412 (2%)** | 764 (4%) | 65 (0%) |
| abdominal pain | 18552 (94%) | **376 (2%)** | 718 (4%) | 59 (0%) |
| **Restricting to episodes with at least 3 visits within 35 days, including the index positive** | | | | |
| any evidence of symptoms | 2132 (39%) | **1759 (32%)** | 486 (9%) | 1131 (21%) |
| fatigue weakness | 4009 (69%) | **1044 (18%)** | 395 (7%) | 341 (6%) |
| headache | 4120 (71%) | **914 (16%)** | 425 (7%) | 330 (6%) |
| cough | 4160 (72%) | **819 (14%)** | 392 (7%) | 418 (7%) |
| loss of taste | 4591 (79%) | **760 (13%)** | 246 (4%) | 192 (3%) |
| muscle ache myalgia | 4488 (78%) | **731 (13%)** | 348 (6%) | 222 (4%) |
| loss of smell | 4660 (80%) | **714 (12%)** | 228 (4%) | 187 (3%) |
| sore throat | 4641 (80%) | **621 (11%)** | 316 (5%) | 211 (4%) |
| shortness of breath | 4950 (86%) | **554 (10%)** | 158 (3%) | 127 (2%) |
| fever | 4826 (83%) | **488 (8%)** | 305 (5%) | 170 (3%) |
| nausea vomiting | 5247 (91%) | **335 (6%)** | 131 (2%) | 76 (1%) |
| diarrhoea | 5371 (93%) | **261 (5%)** | 112 (2%) | 45 (1%) |
| abdominal pain | 5416 (94%) | **227 (4%)** | 106 (2%) | 40 (1%) |

Note: symptoms are ordered from highest to lowest percentage of positive episodes where the symptom was absent at index positive, but present at at least one subsequent visit in the next 35 days
